# Stereo- and Chemodivergent NHC-Promoted Functionalisation of Arylalkylketenes with Chloral**

**DOI:** 10.1002/chem.201503308

**Published:** 2015-09-25

**Authors:** James J Douglas, Gwydion Churchill, Alexandra M Z Slawin, David J Fox, Andrew D Smith

**Affiliations:** [a] EaStCHEM, School of Chemistry, University of St Andrews North Haugh, St Andrews, Fife, KY16 9ST (UK) E-mail: ads10@st-andrews.ac.uk Homepage: http://ch-www.st-andrews.ac.uk/staff/ads/group/; [b] AstraZeneca, Pharmaceutical Development Macclesfield, Cheshire, SK10 2NA (UK); [c] Department of Chemistry, University of Warwick Gibbet Hill Road, Coventry, CV7 4AL (UK) E-mail: d.j.fox@warwick.ac.uk

**Keywords:** asymmetric catalysis, chlorination reactions, ketenes, lactones, stereodivergent reactions

## Abstract

Stereo- and chemodivergent enantioselective reaction pathways are observed upon treatment of alkylarylketenes and trichloroacetaldehyde (chloral) with N-heterocyclic carbenes, giving selectively either β-lactones (up to 88:12 dr, up to 94 % *ee*) or α-chloroesters (up to 94 % *ee*). Either 2-arylsubstitution or an α-branched *i*Pr alkyl substituent within the ketene favours the chlorination pathway, allowing chloral to be used as an electrophilic chlorinating reagent in asymmetric catalysis.

The ability of N-heterocyclic carbenes (NHCs) to catalyse an array of organocatalytic reaction sequences is widely recognised,[1] with recent investigations demonstrating their unique versatility in processes that proceed via reactive acyl anion,[2] azolium enolate,[3] azolium homoenolate,[4] acyl azolium,[5] or α,β-unsaturated acyl azolium species.[6] Typically, α-functionalised aldehydes,[7] enals,[8] activated esters[9] or recently carboxylic acids[10] can be used as mono-substituted azolium enolate precursors, with disubstituted azolium enolates generated using isolable alkylarylketenes. In the latter area, a number of asymmetric formal [2+2],[11] [3+2][12] and [4+2][13] cycloaddition reactions have been developed. Within these processes, limited variation within the alkylarylketene unit is typically tolerated, with 2-substitution of the aryl unit or α-branching in the alkyl substituent usually leading to either no reaction or markedly reduced product yields and stereoselectivity.[13], [14] In only a single isolated [2+2] reaction process that employs 2-oxoaldehydes as the cycloaddition partner is an alkylarylketene bearing either of these structural features vital for high diastereo- and enantiocontrol.[15] Despite these observations, no rationale has been reported, nor a systematic study undertaken to explore the observed product distributions with variation in ketene substitution. In this context, we demonstrate herein that chemo- and stereodivergent reaction pathways are observed in the NHC-mediated asymmetric functionalisation of alkylarylketenes with chloral, generating selectively either β-lactones (up to 88:12 dr, up to 94 % *ee*) or α-chloroesters (up to 94 % *ee*).[16] Notably, 2-arylsubstitution or α-branching within the alkyl chain of the ketene leads to the chlorination pathway (Figure 1), demonstrating, to the best of our knowledge, the ability of chloral to act as an electrophilic chlorine source in asymmetric catalysis for the first time.

**Figure 1:**
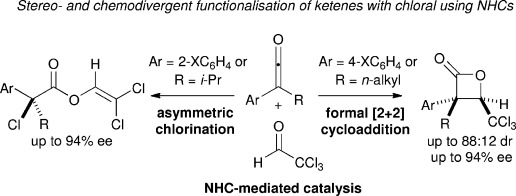
Stereo-and chemodivergent functionalization of ketenes with chloral using NHCs.

Initial investigations focused upon the functionalisation of ethylphenylketene **1** (1.0 eq) with chloral (1.0 eq) using NHC precatalyst **7** (Scheme 1). KHMDS was required for optimal reactivity and selectivity,[17] while decreasing the reaction temperature to lower than 0 °C gave increased enantioselectivity at the expense of product yield.[18] Following optimization this reaction process was suitable for preparative scale reactions (15 mmol of ketene) using 2.5 mol % of precatalyst **7**, giving a 74:26 *anti*:*syn* mixture of separable diastereoisomers *anti*-**2** (2.40 g, 94 % *ee*) and *syn*-**3** (0.96 g, 92 % *ee*) in 80 % overall yield. Both *anti*-**2** and *syn*-**3** were crystallized to enantiopurity,[19] and their relative and absolute configurations unambiguously confirmed by single crystal X-ray diffraction, consistent with high stereocontrol at C(3) imparted by the NHC derived from precatalyst **7**.[20] Remarkably, employing ethyl-1-naphthylketene **4** with precatalyst **7** and chloral, resulted in a chemodivergent reaction process, giving exclusively the tertiary chlorinated vinyl ester **5**. Performing this reaction on a 3.5 mmol scale gave exclusively **5** (1.10 g) in 86 % yield and 94 % *ee*. Derivatisation of **5** with (*S*)-phenylethylamine gave amide **6** as a single diastereoisomer whose absolute configuration was unambiguously confirmed by single crystal X-ray diffraction.[20] To the best of our knowledge, this represents a unique example of chloral acting as an electrophilic chlorinating reagent in asymmetric catalysis and also a rare chemodivergent NHC-promoted process employing ketenes.

**Scheme 1:**
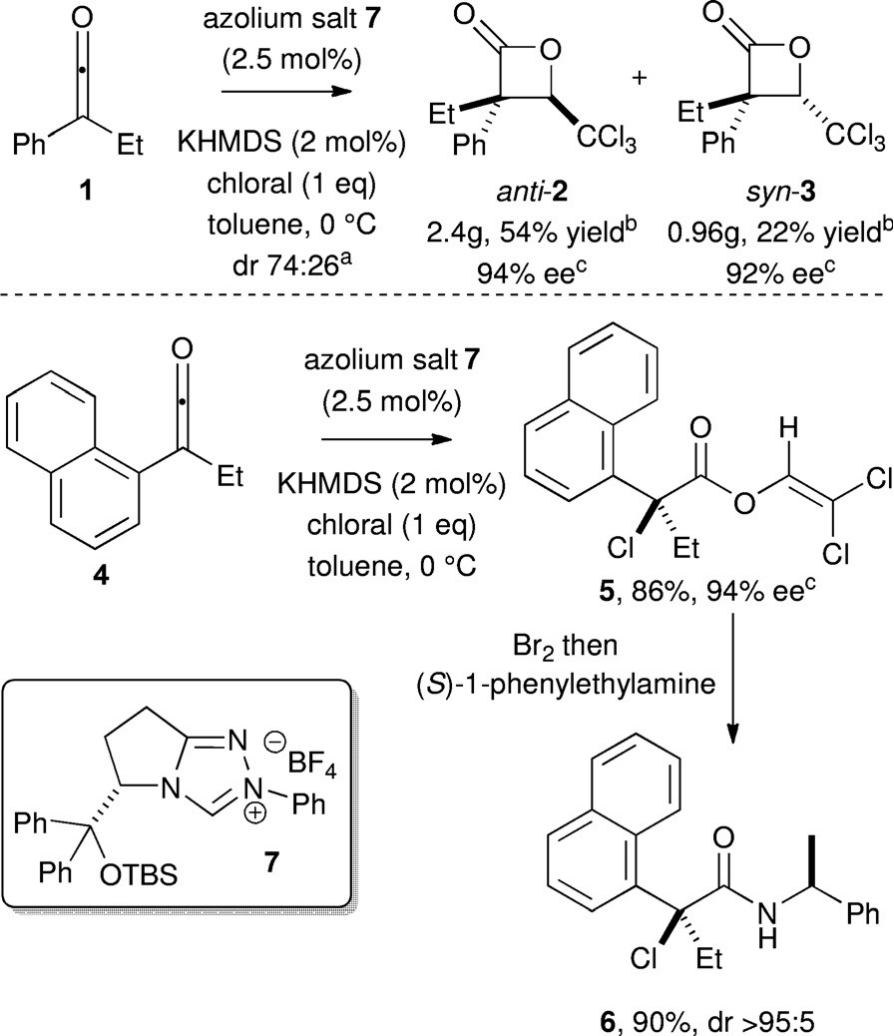
[a] Determined by ^1^H NMR spectroscopic analysis of the crude reaction product. [b] Isolated yield of separable diastereoisomers (dr≥95:5). [c] Determined by chiral HPLC analysis.

The structural parameters within the ketene that govern the outcome of these chemodivergent reaction processes were next examined systematically. 4-Substitution of the aryl unit with either electron-donating or electron-withdrawing substituents is tolerated, leading to exclusive β-lactone formation, in moderate dr (up to 75:25 *anti*:*syn*) and good *ee* (up to 94 % *ee* for the major diastereoisomer) (Scheme 2, **8**–**11**).

**Scheme 2:**
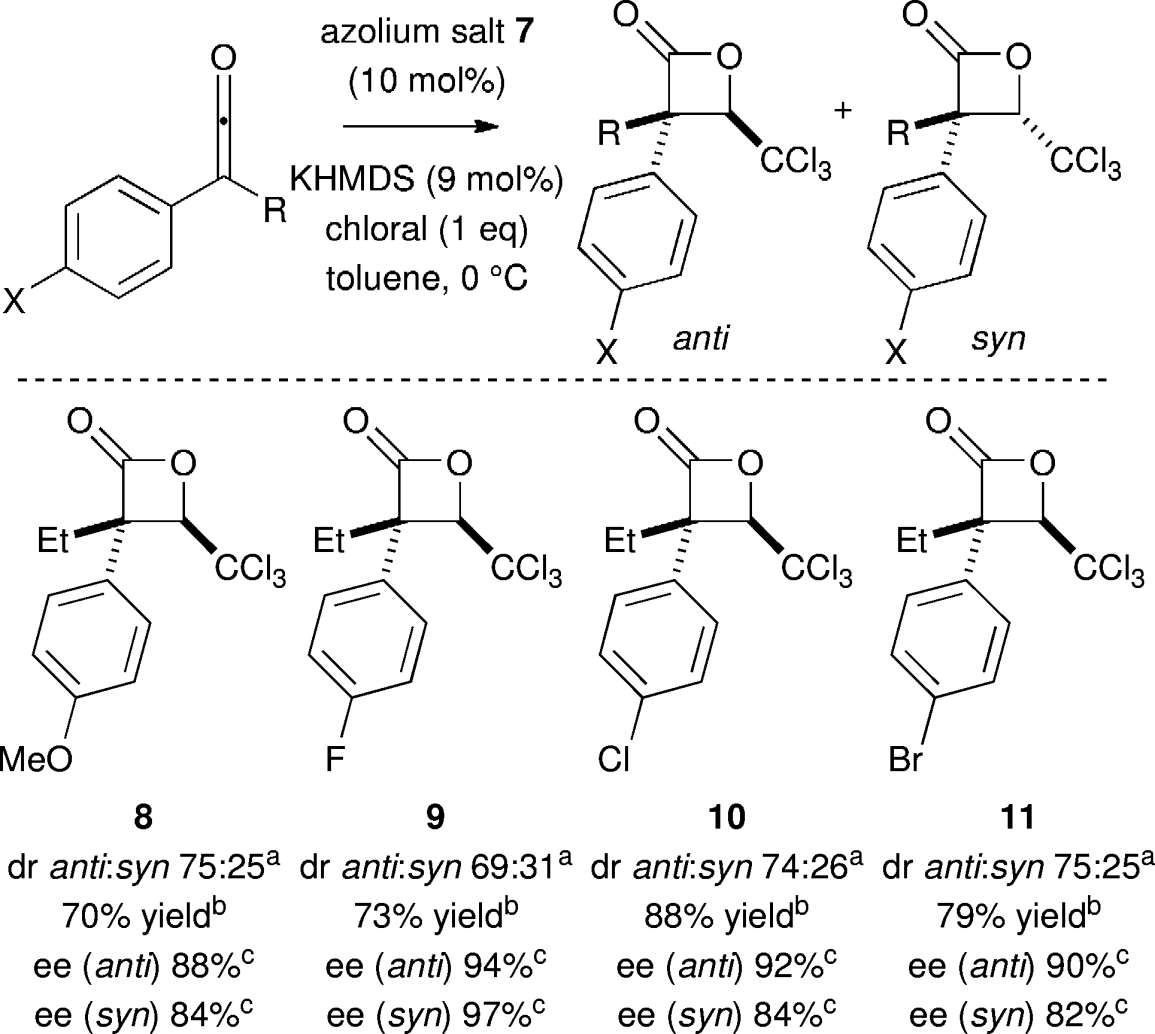
[a] Determined by ^1^H NMR spectroscopic analysis of the crude reaction product. [b] Overall combined isolated yield of separable diastereoisomers. [c] Determined by chiral HPLC analysis.

Subsequent variation of the alkyl unit within a series of alkylphenylketenes showed that highest diastereoselectivity was observed with methylphenylketene (**15**, 95 % yield, 88:12 dr, 82 % *ee*). Further variation of the *n*-alkyl substituent (to give **2**, Et and **13**, *n*Bu) gave slightly reduced *anti*-selectivity with increasing chain length (up to 74:26 dr and 94 % *ee*). *i*Bu Substitution gave **14** with essentially no diastereoselectivity (42:58 dr *anti*:*syn*) with the major *syn*-diastereoisomer isolated in 84 % *ee*. Interestingly, using the α-branched *iso*-propylphenylketene **15** in this protocol gave exclusively α-chloroester **16** in preference to β-lactone formation, giving **16** in 86 % yield and 88 % *ee* (Scheme 3). These trends indicate that both diastereoselectivity in β-lactone formation, and the dichotomy between β-lactone formation and α-chlorination pathways, are sensitive to steric effects of the alkyl ketene substituent.

**Scheme 3:**
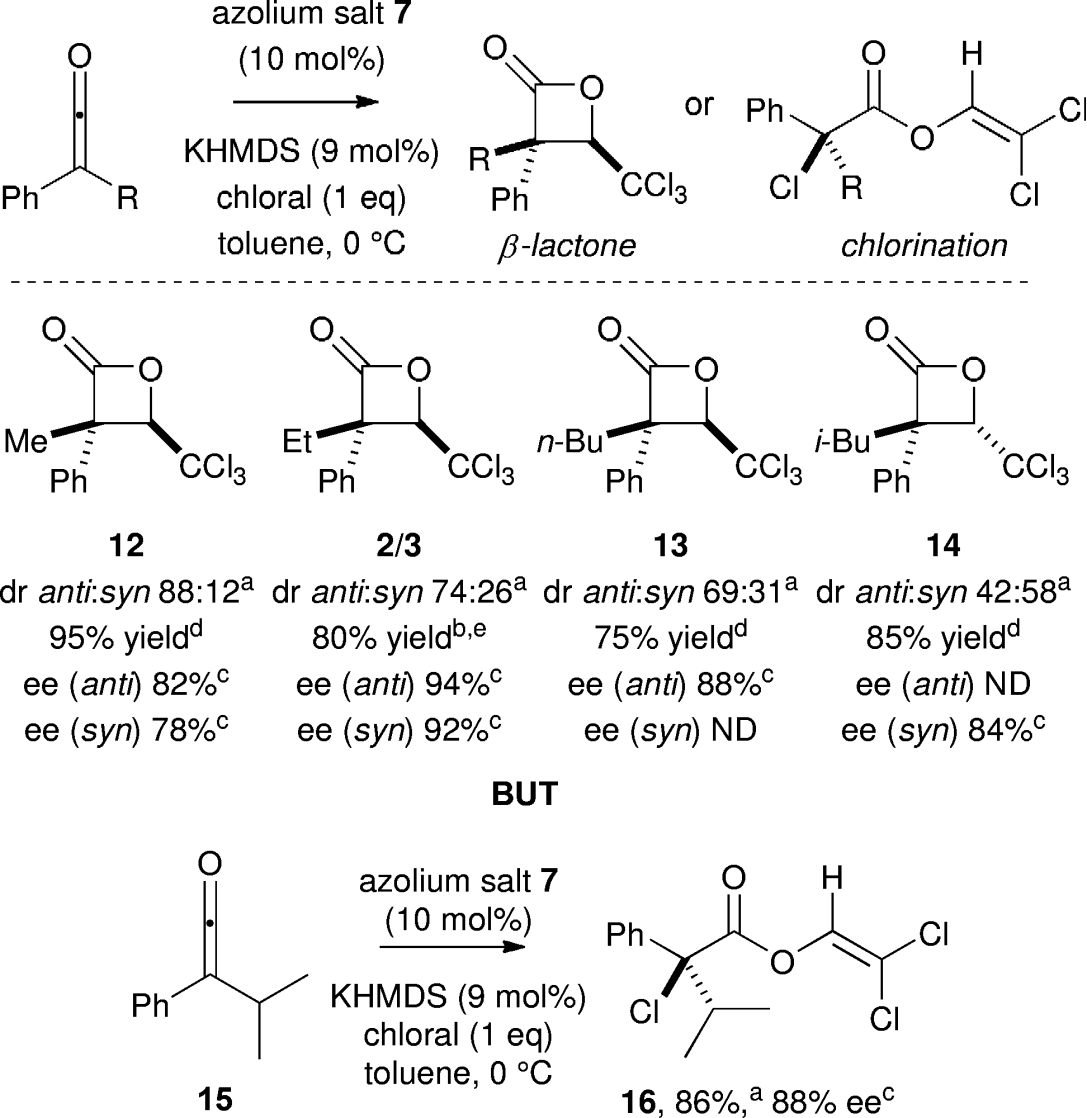
[a] Determined by ^1^H NMR spectroscopic analysis of the crude reaction product. [b] Isolated yield of separable diastereoisomers. [c] Determined by chiral HPLC. [d] Isolated yield of partially separable diastereoisomers.

To further establish the generality of the chlorination pathway, alternative 2-substituted aryl units within the alkylarylketene were evaluated (Scheme 4). Notably, 1-naphthyl-, 2-tolyl- or 2-chlorophenyl-substituted alkylarylketenes led to exclusive formation of the corresponding α-chloroesters **5**, **17**-**21** in good to excellent yield and enantioselectivities (up to 86 % yield and 94 % *ee*).

**Scheme 4:**
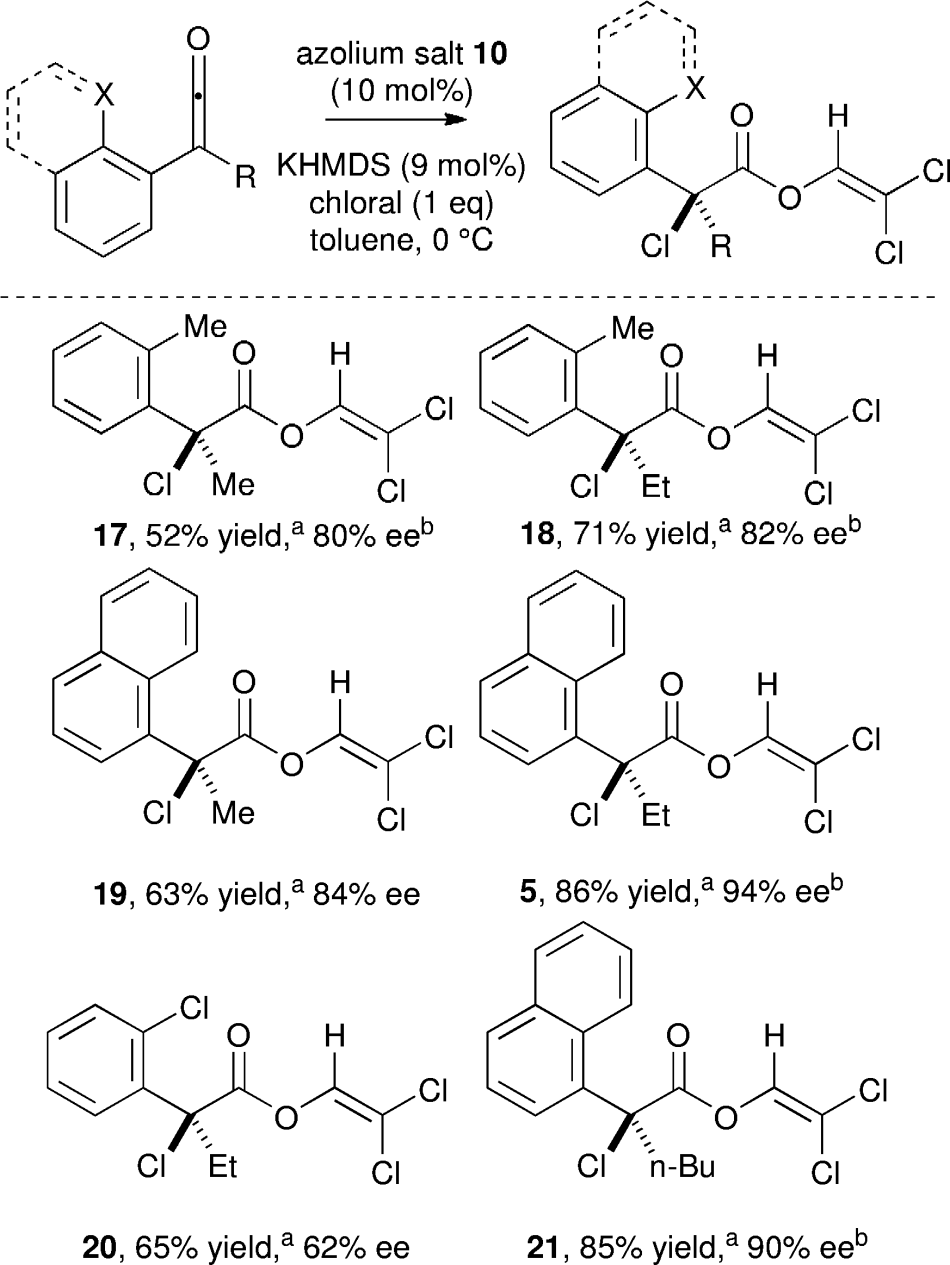
[a] Yield of isolated product. [b] Determined by chiral HPLC analysis.

The observed change in reactivity from formal [2+2] cycloaddition to chlorination with variation in the ketene structure was also investigated computationally using 1,4-dimethyltriazol-5-ylidene as a model NHC catalyst with methyl-2-methylphenylketene and isopropylphenylketene (Figure 2). Grimme’s B3LYP-D3(BJ) functional[21] and the 6-31G(d, p) basis set[22] were used for geometry optimisation and ZPE calculation, with final energies calculated using the TZVPP basis set.[23] Using these constraints, transition structures for both the formal [2+2] cycloaddition and α-chlorination reactions from methyl-2-methylphenylketene and *iso*-propylphenylketene were located (Figure 2). In accordance with the results of Zhang et al.[24] the transition states for reactions of the (*E*)-enolates were significantly lower in energy than those of the (*Z*)-enolates (see SI for all calculated transition state structures and energies). Using both of these ketenes, transition states for α-chlorination over the formal [2+2] cycloaddition process leading to the β-lactones were favoured significantly as observed experimentally. For β-lactone formation, the transition state leading to the *syn*-product was favoured over the *anti*-.[25] In the calculated transition states, the forming C–C bonds in the formal [2+2] cycloaddition are significantly shorter (**22**, 1.88 Å; **23**, 1.87 Å) than the developing C–Cl bonds (**24**, 2.24 Å; **25**, 2.37 Å). This is consistent with the electrophilic chlorine in the S_N_2-type chlorination transition state being less sterically demanding than the sp^2^-hybridised carbonyl carbon in the formal [2+2] cycloaddition reaction. With either a 2-substituent within the aromatic substituent of the alkylarylketene, or a branched *iso*-propyl group, the additional steric encumbrance of these substituents disfavours the formal [2+2] addition, resulting in the chlorination process being preferred.

**Figure 2:**
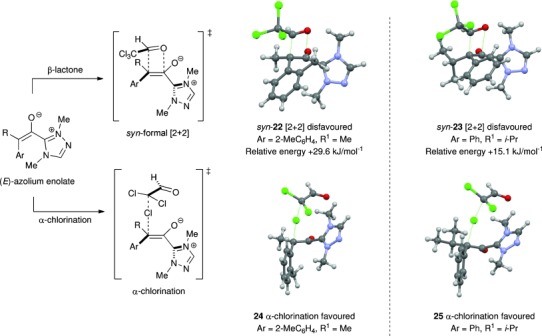
Computed transition states for β-lactone formation and α-chlorination using methyl-2-methylphenylketene and *iso*-propylphenylketene.

Building upon this model, the observed chemodivergent reaction pathways are proposed to arise from initial NHC addition to the ketene, with preferential onwards reaction arising from the (*E*)-azolium enolate **26**. Subsequent stereoselective formal [2+2] cycloaddition with chloral generates **28**, with elimination of the NHC giving the β-lactone and completing the catalytic cycle. Alternatively, the use of chloral as an electrophilic chlorinating agent results in the formation of an acyl azolium and enolate ion pair **27** that combined to give the observed α-chloroester. Notably, assuming these mechanistic extremes, stereodivergent reaction pathways are observed from the (*E*)-azolium enolate intermediate **26**. *Re*-face functionalisation of the enolate derived from sterically non-demanding ketenes (such as **1**) leads to the observed β-lactone configuration. Conversely, *Si*-face functionalisation with ketenes bearing either a 2-substituted aryl unit or an α-branched *iso*-propyl substituent provides the configuration observed for the chlorinated esters (Figure 3).

**Figure 3:**
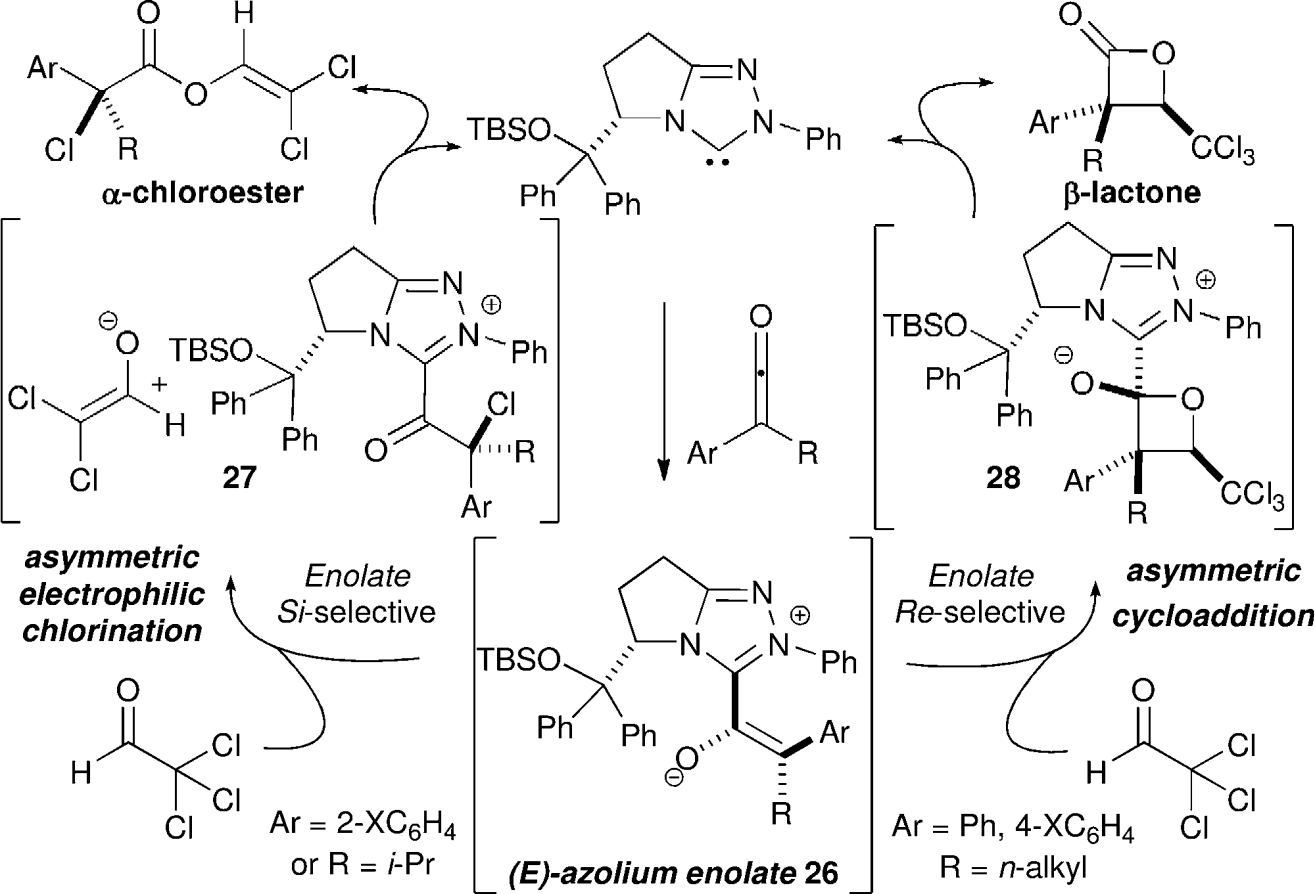
Proposed catalytic cycle.

## Conclusion

To conclude, stereo- and chemodivergent asymmetric reaction pathways are observed upon treatment of alkylarylketenes and chloral with chiral NHCs, giving selectively either β-lactones (up to 88:12 dr, up to 94 % *ee*) or α-chloroesters (up to 94 % *ee*), with 2-arylsubstitution or α-branching within the alkyl chain of the ketene unit leading to the α-chlorination pathway. Computational studies on a model system have allowed the structural parameters that lead to selectivity in these reaction processes to be analysed. Current research from this laboratory is directed toward developing alternative uses of NHCs and other Lewis bases in asymmetric catalysis.

## Experimental Section

For general experimental details, full characterisation data, NMR spectra and HPLC traces, see the Supporting Information.

### General procedure (1): Lactonisation and chlorination at 0 °C

To a flame dried Schlenk flask under an argon atmosphere was added NHC precatalyst (0.10 mmol), base (0.09 mmol) and toluene (6 mL) and the mixture stirred for 15 min. The mixture was then cooled to 0 °C in an ice/H_2_O bath followed by addition of a 0 °C solution of the requisite ketene (1.00 mmol) in toluene (12 mL), immediately followed by chloral (1.00 mmol). Toluene (2 mL) was added to wash residual reactants into solution and the reaction was stirred for the stated time at 0 °C before opening the flask to the air for 30 min and concentration in vacuo. The resulting crude residue was purified by flash silica chromatography (ether:petrol) to provide either the isolated lactone or chlorinated ester as stated.

### General procedure (2): Lactonisation and chlorination at 0 °C with dropwise ketene addition

In instances where ketene dimerization was competitive with lactonisation or chlorination the ketene was added dropwise. To a flame dried Schlenk flask under an argon atmosphere was added NHC precatalyst (0.10 mmol), base (0.09 mmol) and toluene (6 mL) and the mixture stirred for 15 min. The mixture was then cooled to 0 °C in an ice/H_2_O bath followed by addition of chloral (1.00 mmol). A 0 °C solution of the requisite ketene (1.00 mmol) in toluene (12 mL) was subsequently added over 0.5 h. The reaction was stirred for an additional 3 h at 0 °C before opening the flask to the air for 0.5 h and concentration in vacuo. The resulting crude residue with the stated diastereomeric ratio was purified by flash silica chromatography (ether:petrol) to provide either the isolated lactone or chlorinated ester.

## Supporting Information

As a service to our authors and readers, this journal provides supporting information supplied by the authors. Such materials are peer reviewed and may be re-organized for online delivery, but are not copy-edited or typeset. Technical support issues arising from supporting information (other than missing files) should be addressed to the authors.

miscellaneous_information
