# Supplementary material for: Stereo- and Chemodivergent NHC-Promoted Functionalisation of Arylalkylketenes with Chloral
Source: Chemistry. 2015 Sep 25;21(46):16354–8. doi: 10.1002/chem.201503308 (PMC4648049; doi:10.1002/chem.201503308)
Supplement: Supplementary file 1 — miscellaneous_information [file chem0021-16354-sd1.pdf]

CHEMISTRY
A European Journal

Supporting Information

Stereo- and Chemodivergent NHC-Promoted Functionalisation of Arylalkylketenes with Chloral**

James J. Douglas,[a, d] Gwydion Churchill,[b] Alexandra M. Z. Slawin,[a] David J. Fox,*[c] and Andrew D. Smith*[a]

chem_201503308_sm_miscellaneous_information.pdf

Stereo- and chemodivergent NHC-promoted functionalisation of arylalkylketenes with chloral

James J. Douglas, Gwydion Churchill, Alexandra M. Z. Slawin, David J. Fox and Andrew D. Smith[a]

Supporting Information
Contents

General Information......2
Optimization......3
Synthesis of Acid Chlorides......4
Synthesis of Ketenes......5
Synthesis of Precatalyst 7......10
Synthesis of Lactones and Chlorinated Products.......13
Additional Computational Information......25
HPLC Spectrum......31
NMR Spectrum......54
References......83

General Information

Reactions involving moisture sensitive reagents were carried out under an argon atmosphere using standard vacuum line techniques in addition to dry solvents. All glassware used was flame dried and cooled under vacuum. Solvents (THF, CH_2Cl_2, toluene, hexane and ether) were obtained anhydrous and purified by an alumina column (Mbraun SPS-800). Petrol is defined as petroleum ether 40-60 °C. All other solvents were used as supplied without further purification unless stated otherwise. Unless stated chemicals were purchased from Acros-Uk, Sigma-Aldrich, Alfa Aeasar or Fisher. Potassium bis(trimethylsilyl)amide (KHMDS) was used as a 0.5 M solution in toluene as supplied (Aldrich). Chloral was distilled from CaH_2 before use. The required aldehydes where purified by kugelrohl distillation under reduced pressure prior to use. 2,2,6,6-tetrachlorocyclohexanone (Aldrich) was recrystallised from CH_2Cl_2/pentane. Room temperature (rt) refers to 20-25 °C. Temperatures of 0 °C and −78 °C were obtained using ice/H_2O and CO_2(s)/acetone baths respectively. Temperatures of 0 °C to −50 °C were obtained using an immersion cooler (HAAKE EK 90). Reflux conditions were obtained using an oil bath equipped with a contact thermometer. *In vacuo* refers to the use of a Büchi Rotavapor R-2000 rotary evaporator with a Vacubrand CVC_2 vacuum controller or a Heidolph Laborota 4001 rotary evaporator with a vacuum controller. Analytical thin layer chromatography was performed on pre-coated aluminium plates (Kieselgel 60 F_{254} silica). TLC visualisation was carried out with ultraviolet light (254 nm), followed by staining with a 1% aqueous $KMnO_4$ solution. Flash silica chromatography was performed on Kieselgel 60 silica in the solvent system stated. ^{1}H, ^{13}C and ^{19}F nuclear magnetic resonance (NMR) spectra were acquired on either a Bruker Avance 300 (300 MHz, ^{1}H, 75 MHz ^{13}C), a Bruker Avance II 400 (400 MHz, ^{1}H, 376 MHz 19F, 100 MHz ^{13}C) or a Bruker Ultrashield 500 (500 MHz, ^{1}H, 125 MHz ^{13}C) spectrometer at ambient temperature in the deuterated solvent stated. All chemical shifts are quoted in parts per million (ppm) relative to the residual solvent as the internal standard. All coupling constants, *J*, are quoted in Hz and reported low to high. Multiplicities are indicated by: s (singlet), d (doublet), t (triplet), q (quartet), sept (septet), oct (octet), m (multiplet), dd (doublet of doublets), ddd (doublet of doublet of doublets, dt (doublet of triplets), dq (doublet of quartets) and td (triplet of doublets). The abbreviation Ar is used to denote aromatic, br to denote broad, Bn to denote benzyl and *app* to denote apparent. Infrared spectra (ν_{max}) were recorded on either a Perkin-Elmer Spectrum GX FT-IR spectrometer using either thin films on NaCl plates or KBr discs or a Shimadzu IRAffinity-1 using a Pike attenuated total reflectance (ATR) accessory.

Melting points were recorded on an Electrothermal 9100 melting point apparatus and are uncorrected. *Decomp* refers to decomposition. HPLC analyses were obtained on either a Gilson HPLC consisting of a Gilson 305 pump, Gilson 306 pump, Gilson 811C dynamic mixer, Gilson 805 manometric module, Gilson 401C dilutor, Gilson 213XL sample injector and sample detection was performed with a Gilson 118 UV/vis detector. Or a Shimadzu HPLC consisting of a DGU-20A5 degasser, LC-20AT liquid chromatograph, SIL-20AHT autosampler, CMB-20A communications bus module, SPD-M20A diode array detector and a CTO-20A column oven which allowed the temperature to be set from 25-40 °C. Separation was achieved using the DACIEL CHIRALPAK column stated. All chiral HPLC traces were compared to the authentic racemic spectrum prepared in analogous fashion.

Mass spectrometry (*m/z*) data were acquired by electrospray ionisation (ESI), electron impact (EI), atmostpheric solids analysis probe (ASAP) or nanospray ionisation (NSI) at the EPSRC National Mass Spectrometry Service Centre, Swansea. Optical rotations were measured on a Pertin Elmer Precisely/Model-341 polarimeter operating at the sodium D line with a 100 mm path cell at rt.

Optimization

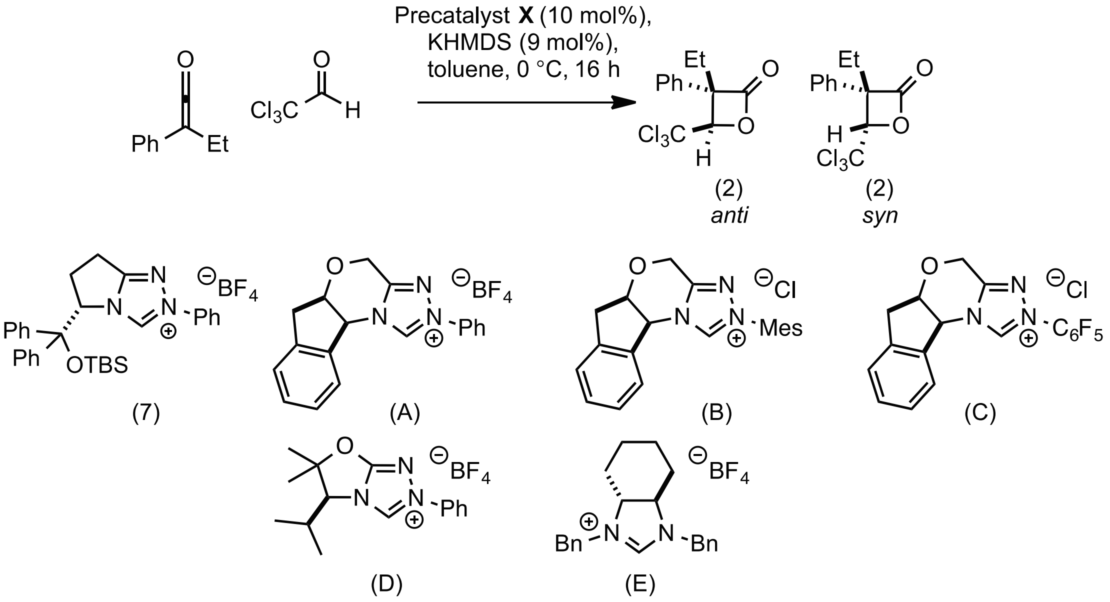

Entry	Precatalyst	*dr*[a] (*anti*:*syn*)	Yield (%)[b] (*anti*:*syn*)	*ee* (%)[c] (*anti*:*syn*)
1	66	72:28	61, 28	92, 94
2	A	83:17	59, 17	91 (*ent*), 84 (*ent*)
3	B	70:30	31, 12	10, 7
4	C	–,–	NR	–,–
5	D	77:23	12, 22	10, 39 (*ent*)
6	E	63:37	34, 20	17, <5

[a] Calculated by inspection of the crude reaction mixture using ^{1}H NMR spectroscopy [b] Combined isolated yield of single diastereomer. [c] % *ee* determined by HLPC analysis on a chiral stationary phase.

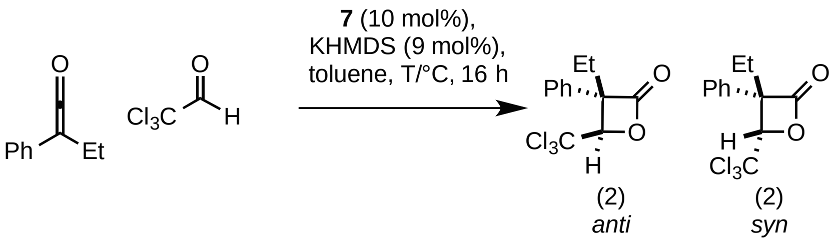

Entry	Temp (°C)	Conversion (%)[a]	*dr*[a] (*syn*:*anti*)	Yield (%)[b] (*syn*:*anti*)	*ee* (%)[c] (*syn*:*anti*)
1[d]	rt	50	69:31	16, –	94 , –
2	–25	50	74:26	–,–	–,–
3	–78 to rt	50	85:15	–,–	–,–
4[e]	rt	>75	53:46		

[a] Calculated by inspection of the crude reaction mixture using ^{1}H NMR [b] Isolated yield of single diastereomer. [c] % *ee* determined by HLPC analysis on a chiral stationary phase. [d] 9 mol% Cs_2CO_3 used as the base. [e] Reaction performed in the absence of precatalyst **7**.

Synthesis of Acid Chlorides

All acid chlorides previously reported in the literature were prepared *via* the general procedure of Smith[1]

Thionyl chloride (3 equiv) was added to a solution of the corresponding acid (1 equiv) in toluene at room temperature and the reaction mixture was warmed to 80 °C and stirred 12 hours. Solvents were evaporated under reduced pressure and the crude oil purified by Kugelrohr distillation to give the corresponding acid chloride.

(S1)-2-(naphthalen-2-yl)propanoyl chloride

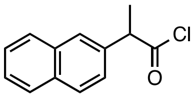

Following the procedure reported by Fu:[2] *n*-Buli 2.5M (47.3 mL, 118 mmol moles, 2.2 equiv) was added dropwise at –78 °C over 15 mins to a solution of 1-napthylacetic acid (10.0g, 57.7 mmoles, 1.0 equiv) in THF (200mL). The solution was then warmed to rt and stirred for a further 45 mins before methyliodide (5.01 mL, 80.6 mmol, 1.5 equiv) was added and the solution stirred overnight at rt. The reaction was quenched *via* carful addition of 2M HCl (75 mL) before extraction with Et_2O (3 × 100 mL) and drying over $MgSO_4$. Filtration and concentration *in vacuo* yielded 6.8g of crude 2-(naphthalen-2-yl)propanoic acid that was used without further purification.

Thionyl chloride (3.70 mL, 51.2 mmol, 1.5 equiv) was added dropwise to a solution of crude

2-(naphthalen-2-yl)propanoic acid (6.8g, 34.1 mmol, 1.0 equiv) in toluene 50 mL at 75 °C and stirred for 3h. Concentration and kugelrohr distillation 190 – 200 °C (5 mbar) provided the title compound as a pale orange oil; (6.0g, 47% yield over 2 steps); ν_{max} (thin film)/cm^{-1} 3062, 2985, 1834, 1787 (C=O), 1598, 1512, 918, and 778; ^{1}H NMR (400 MHz, $CDCl_3$) δ_H 1.77 (3H, d, *J* 7.0), 4.92 (1H, q, *J* 7.0), 7.45-7.53 (2H, m), 7.55-7.64 (2H, m), 7.87 (1H, d, *J* 7.9), 7.93 (1H, dd, *J* 1.2, 8.3) and 8.03 (1H, d, *J* 8.4); ^{13}C NMR (100 mHz $CDCl_3$): δ_C 18.8, 53.5, 122.8, 125.7, 125.9, 126.3, 127.2, 129.2, 129.5, 131.4, 134.2, 134.4 and 176.3.

(S2) 2-(naphthalen-2-yl)hexanoyl chloride

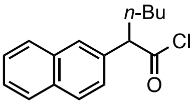

Following the procedure reported by Fu:[2] *n*-Buli 2.5M (47.3 mL, 118 mmol moles, 2.2 equiv) was added dropwise at –78 °C over 15 mins to a solution of 1-napthylacetic acid (10.0g, 57.7 mmol, 1.0 equiv) in THF (200mL). The solution was then warmed to rt and stirred for a further 45 mins before 1-bromobutane (12.4 mL, 115 mmol, 2.0 equiv) was added and the solution stirred overnight at rt. The reaction was quenched *via* carful addition of 5% HCl (75 mL) before extraction with Et_2O (3 × 100 mL) and drying over $MgSO_4$. Filtration and concentration *in vacuo* yielded 13.9g of crude 2-(naphthalen-2-yl)hexanoic acid that was used without further purification.
Thionyl chloride (6.30 mL, 86.6 mmol, 1.5 equiv) was added dropwise to a solution of crude 2-(naphthalen-2-yl)hexanoic acid (13.9g, 57.7 mmol, 1.0 equiv) in toluene 50 mL at 75 °C and stirred for 3h. Concentration and kugelrohr distillation 200 – 220 °C (5 mbar) provided the title compound as a pale colourless oil; (11.9g, 79% yield over 2 steps); ν_{max} (thin film)/cm^{-1} 3051, 2958, 1790 (C=O), 1598, 1513, 1466 and 779; ^{1}H NMR (400 MHz, $CDCl_3$) δ_H 0.88 (3H, t, *J* 6.6), 1.26-1.44 (4H, m) 1.94-2.03 (1H, m), 2.31-2.4 (1H, m, *n*-Bu), 4.78 (1H, t, *J* 7.3), 7.45-7.55 (3H, m), 7.57-7.61 (1H, m), 7.86 (1H, d, *J* 7.7) 7.91 (1H, d, *J* 8.0) and 8.06 (1H, d, *J* 8.5); ^{13}C NMR (100 mHz $CDCl_3$): δ_C 14.0, 22.7, 29.8, 33.3, 58.9, 122.8, 125.8, 126.0, 126.2, 127.1, 129.0, 129.4, 131.7, 132.7, 134.3 and 175.5.

Synthesis of Ketenes

Representative procedure: ethylphenylketene 1

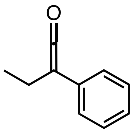

A flame dried two-neck round bottom flask separated by a sintered adaptor to a second two-neck round bottom flask under an argon atmosphere is charged with strictly anhydrous ether (45 mL), 2-phenylbutanoyl chloride (3.00 g, 16.4 mmol, 1.0 equiv) and cooled to 0 °C. Dropwise addition over 30 min of Et_3N, (2.52 mL, 18.1 mmol, 1.1 equiv) formed a bright yellow solution and a white precipitate that was stirred overnight at 0 °C. The solution was then warmed to rt after which filtration under argon and concentration gave a bright yellow oil; Transfer *via* cannula to a flame dried kugelrohl

flask and distillation 80-90 °C (5 mbar) {literature 70 °C (0.5 torr)}[3] gave ethylphenylketene as a light yellow oil; (1.4 g, 60% yield); ^{1}H NMR (300 MHz, $CDCl_3$) δ_H 1.24 (3H, t, *J* 7.4), 2.44 (2H, q, *J* 7.4), 7.02-7.10 (3H, m) and 7.28-7.35 (2H, m).

Methylphenylketene

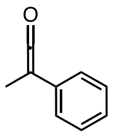

Following the representative procedure: 2-Phenylpropanoyl chloride (4.05g, 24.0 mmol, 1.0 equiv), Et_3N (3.34 mL, 24.0 mmol, 1.0 equiv) in ether (50 mL) at 0 °C overnight gave, after kugelrohl distillation 60-80 °C (5 mbar) {literature 50 °C, (4 torr)}[3] a yellow/orange oil; (1.44 g, 45% yield); ^{1}H NMR (400 MHz, $CDCl_3$) δ_H 1.92 (3H, s), 6.93-6.95 (2H, m), 6.97-7.01 (1H, m) and 7.21-7.25 (2H, m).

Butylphenylketene

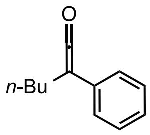

Following the representative procedure: 2-Phenylhexanoyl chloride (3.00 g, 14.2 mmol, 1.0 equiv), Me_2EtN (1.70 mL, 15.7 mmol, 1.1 equiv) in ether (45 mL) at 0 °C overnight gave, after kugelrohl distillation 110-120 °C (5 mbar) {literature 88 °C (1.5 torr)}[4] a yellow/orange oil; (1.26 g, 51% yield); ^{1}H NMR (300 MHz, $CDCl_3$) δ_H 0.87 (3H, t, *J* 7.2), 1.30-1.53 (4H, m), 2.32 (2H, t, *J* 7.4), 6.93-7.01 (3H, m) and 7.19-7.25 (2H, m).

***i*-Butylphenylketene**

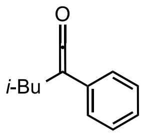

Following the representative procedure: 4-methyl-2-phenylpentanoyl chloride (3.80 g, 18.0 mmol, 1.0 equiv), Me_2EtN (2.15 mL, 20.0 mmol, 1.1 equiv) in ether (45 mL) at 0 °C overnight gave, after kugelrohl distillation 110-117 °C (5 mbar) {literature 37-46 °C, (0.8 torr)}[5] a yellow/orange oil; (1.75g, 56% yield); ^{1}H NMR (300 MHz, $CDCl_3$) 0.92 (6H, d, *J* 6.6), 1.66-1.83 (1H, m), 2.19 (2H, d, *J* 7.0), 6.95-7.01, (3H, m) and 7.17-7.25 (2H, m).

Ethyl-4-tolylketene

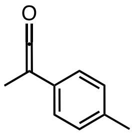

Following the representative procedure: 2-(p-tolyl)butanoyl chloride (2.16 g, 11.0 mmol, 1.0 equiv), Et_3N (1.53 mL, 11.0 mmol, 1.0 equiv) in ether (40 mL) at 0 °C overnight gave, after kugelrohl

distillation 110-120 °C (7 mbar) {literature 68-72 °C, (0.2 torr)}[6] a yellow/orange oil; (0.92 g, 52% yield); ^{1}H NMR (300 MHz, $CDCl_3$) δ_H 1.29 (3H, t, *J* 7.4), 2.40 (3H, s), 2.50 (2H, q, *J* 7.4), 7.02 (2H, d, *J* 8.1) and 7.21 (2H, d, *J* 8.1).

Ethyl-4-chlorophenylketene

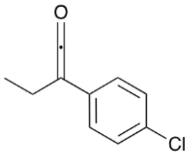

Following the representative procedure: 2-(4-chlorophenyl)butanoyl chloride (763 mg, 3.51 mmol, 1.0 equiv), Et_3N (0.49 mL, 3.51 mmol, 1.0 equiv) in ether (20 mL) at 0 °C overnight gave, after kugelrohl distillation 125-135 °C (7 mbar); a yellow/orange oil; (368 mg, 56% yield); ν_{max} (thin film)/cm^{-1} 2097; ^{1}H NMR (300 MHz, $CDCl_3$) δ_H 1.34 (3H, t, *J* 7.4), 2.53 (2H, q, *J* 7.4), 7.05-7.09 (2H, m) and 7.37-7.42 (2H, m).

Ethyl-4-flourophenylketene

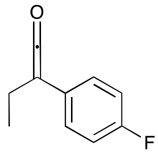

Following the representative procedure: 2-(4-fluorophenyl)butanoyl chloride (2.70 g, 13.5 mmol, 1.0 equiv), $EtMe_2N$ (1.60 mL, 14.8 mmol, 1.1 equiv) in ether (40 mL) at 0 °C overnight gave, after kugelrohl distillation 104-110 °C (7 mbar); a yellow/orange oil; (1.21 g, 55% yield); ν_{max} (thin film)/cm^{-1} 2100; ^{1}H NMR (300 MHz, $CDCl_3$) δ_H 1.13 (3H, t, *J* 7.4), 2.33 (2H, q, *J* 7.4) and 6.87-7.97 (4H, m); ^{13}C NMR (75 MHz $CDCl_3$): δ_C 12.9, 17.5, 41.1, 116.1 (d, *J* 21.7), 125.5 (d, *J* 7.6), 128.5 (d, *J* 3.1), 160.5 (d, *J* 243.4) and 205.7.

Ethyl-4-bromophenylketene

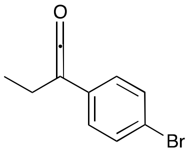

Following the representative procedure: 2-(4-bromophenyl)butanoyl chloride (5.00g, 19.1 mmol, 1.0 equiv), Et_2MeN (2.30 mL, 21.0 mmol, 1.1 equiv) in ether (45 mL) at 0 °C overnight gave, after kugelrohl distillation 180 °C (7 mbar); a yellow/orange oil; (1.21 g, 28% yield); ν_{max} (thin film)/cm^{-1} 2100; ^{1}H NMR (300 MHz, $CDCl_3$) δ_H 1.13 (3H, t, *J* 7.4), 2.33 (2H, q, *J* 7.4), 6.87-7.97 and (4H, m); ^{13}C NMR (75 mHz $CDCl_3$): δ_C 11.7, 15.9, 40.7, 116.2, 124.4, 130.9, and 203.2, 1 x ArC-1 could not be resolved.

Ethyl-4-methoxyphenylketene

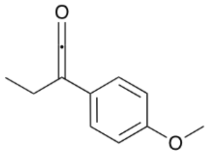

Following the representative procedure: 2-(4-methoxyphenyl)butanoyl chloride (3.00 g, 14.1 mmol, 1.0 equiv), Et_3N (3.93 mL, 28.2 mmol, 2.0 equiv) in ether (45 mL) at 0 °C overnight gave, after kugelrohl distillation 140-150 °C (3 mbar); a yellow/orange oil; (1.19 g, 48% yield); ν_{max} (thin film)/cm^{-1} 2096; ^{1}H NMR (300 MHz, $CDCl_3$) δ_H 1.21 (3H, t, *J* 7.4), 2.41 (2H, q, *J* 7.4), 3.79 (3H, s), 6.86-6.91 (2H, m) and 6.95-6.99 (2H, m).

Methyl-2-tolyl ketene

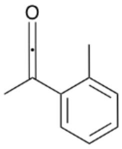

Following the literature procedure of Fu:[8] 2-(o-tolyl)propanoyl chloride (3.55 g, 19.4 mmol, 1.0 equiv), Me_2EtN, added at 0 °C over 5 mins (10.5 mL, 97.2 mmol, 5.0 equiv) in THF (45 mL) then rt overnight gave, after kugelrohl distillation 69 °C (3 mbar) {literature 43 °C, (0.4 torr)}[8] (997 mg, 35% yield); ^{1}H NMR (400 MHz, $CDCl_3$) δ_H 1.97 (3H, s), 2.23 (3H, s), 6.95-7.01 (2H, m), 7.05-7.07 (1H, m) and 7.10-7.15 (1H, m).

Ethyl-2-tolyl ketene

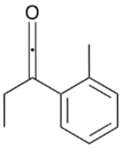

Following the literature procedure of Fu:[5] 2-(o-tolyl)butanoyl chloride (3.45 g, 17.5 mmol, 1.0 equiv), Me_2EtN, added in a single charge (7.60 mL, 70.2 mmol, 4.0 equiv) in THF (40 mL) at 0 °C overnight gave, after kugelrohl distillation 97 °C (3 mbar) {literature 32-33 °C, (0.2 torr)}[5] (997 mg, 35% yield); ^{1}H NMR (300 MHz, $CDCl_3$) δ_H 1.18 (3H, t, *J* 7.4), 2.32 (3H, s), 2.48 (2H, q, *J* 7.4) and 7.07-7.23 (4H, m).

(S3) Butyll-2-tolyl ketene

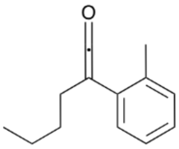

Following the representative procedure: 2-(o-tolyl)hexanoyl chloride (3.39 g, 15.1 mmol, 1.0 equiv), Me_2EtN, added in a single charge (7.40 mL, 68.0 mmol, 4.5 equiv) in THF (45 mL) at 0 °C overnight gave, after kugelrohl distillation 147 °C (3 mbar); (1.56 g, 55% yield); ν_{max} (thin film)/cm^{-1} 2095; ^{1}H

NMR (400 MHz, $CDCl_3$) δ_H 0.94 (3H, t, *J* 7.2), 1.38-1.55 (4H, m), 2.33 (3H, s), 2.46 (2H, t, *J* 7.3) and 7.09-7.24 (4H, m); ^{13}C NMR (75 mHz $CDCl_3$): δ_C 13.9, 20.9, 22.4, 26.4, 30.5, 35.7, 125.7, 126.5, 126.7, 130.6, 131.0, 135.7 and 200.1.

Ethyl-(2-chlorophenyl) ketene

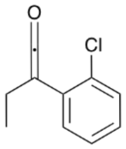

Following the literature procedure of Ye[7]: 2-(2-chlorophenyl)butanoyl chloride (3.00 g, 13.8 mmol, 1.0 equiv), Me_2EtN (5.99 mL, 55.3 mmol, 4.0 equiv) in Et_2O (45 mL) at 0 °C overnight gave, after kugelrohl distillation 137 °C (3 mbar) {literature 65-67 °C, (0.2 torr)}[6] (1.32g, 52% yield); ^{1}H NMR (400 MHz, $CDCl_3$) δ_H 1.14 (3H, t, *J* 7.4), 2.42 (2H, q, *J* 7.4), 6.94-6.98 (2H, m), 7.14-7.18 (1H, m) and 7.24 (1H, dd, *J* 1.3, 8.3).

(S4)-Methyl-2-napthyl ketene

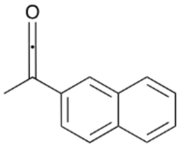

Following the representative procedure: 2-(naphthalen-2-yl)propanoyl chloride (2.79 g, 12.8 mmol, 1.0 equiv), Me_2EtN, added in a single charge (5.50 mL, 51.0 mmol, 4.0 equiv) in THF (25 mL) at 0 °C overnight gave, after kugelrohl distillation 180 °C (3 mbar); (960 mg, 42% yield); ν_{max} (thin film)/cm^{-1} 2099; ^{1}H NMR (400 MHz, $CDCl_3$) δ_H 2.22 (3H, s), 7.35 (1H, dd, *J* 7.3 1.1), 7.46-7.58 (3H, m), 7.73 (1H, d, *J* 8.2), 7.89 (1H, dd, *J* 2.6, 6.8) and 8.04-8.07 (1H, m); ^{13}C NMR (100 mHz $CDCl_3$): δ_C 12.4, 29.3, 124.2, 124.7, 125.9, 126.0, 126.1, 126.8, 129.0, 130.0, 131.1, 134.2 and 200.3.

(4)-Ethyl-2-napthyl ketene

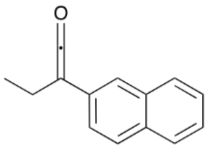

Following the literature procedure of Fu:[9] 2-(naphthalen-2-yl)butanoyl chloride (8.80 g, 37.8 mmol, 1.0 equiv), Me_2EtN, (16.0 mL, 148 mmol, 4.0 equiv) in THF (50 mL) at 0 °C overnight gave, after kugelrohl distillation 175 °C (3 mbar) {literature 80 °C, (0.2 torr)}[9] (3.04 g, 41% yield); ^{1}H NMR (300 MHz, $CDCl_3$) δ_H 1.25 (3H, t, *J* 7.4), 2.63 (2H, q, *J* 7.4), 7.41-7.60 (4H, m), 7.77 (1H, d, *J* 8.0) 7.88-7.91 (1H, m) and 8.08-8.12 (1H, m).

(S5)-Butyl-2-napthyl ketene

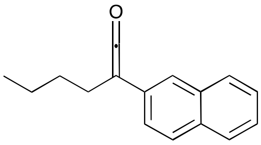

Following the representative procedure: 2-(naphthalen-2-yl)hexanoyl chloride (6.04 g, 23.0 mmol, 1.0 equiv), Me_2EtN, added in a single charge (10.0 mL, 92.6 mmol, 4.0 equiv) in THF (30 mL) at 0 °C overnight gave, after kugelrohl distillation 198 °C (3 mbar); (2.78 g, 54% yield); ν_{max} (thin film)/cm^{-1} 2098; ^1H NMR (300 MHz, $CDCl_3$) δ_H 0.96 (3H, t, *J* 7.2), 1.44-1.60 (4H, m), 2.61 (2H, t, *J* 7.3), 7.42-7.61 (4H, m), 7.78 (1H, d, *J* 7.9), 7.90 (1H, dd, *J* 2.1, 7.4) and 8.11-8.14 (1H, m) ^{13}C NMR (100 mHz $CDCl_3$): δ_C 13.9, 22.4, 27.1, 30.7, 34.2, 124.6, 125.8, 126.0, 126.2, 126.3, 127.1, 128.9, 131.9, 134.3 and 199.4.

***i*-propylphenyl ketene**

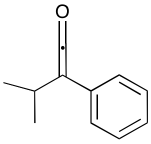

Following the representative procedure: 3-methyl-2-phenylbutanoyl chloride (3.65 g, 18.6 mmol) and Me_2EtN (9.02 mL, 83.5 mmol, 4.5 equiv) in THF at rt overnight gave after Kugelrohr distillation 87-92 °C (5 mbar) {literature 90-94 °C (2 mbar)} [10] (1.48 g, 50%); δ_H (300 MHz, $CDCl_3$) 1.23 (6H, d, *J* 6.7), 2.83 (1H, *app* sept, *J* 6.7), 7.05-7.10 (3H, m) and 7.28-7.34 (2H, m)

Ethyl-2-methoxyphenylketene

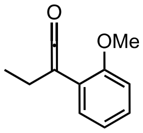

Following the representative procedure: 2-(2-methoxyphenyl)butanoyl chloride (3.81 g, 17.9 mmol, 1.0 equiv), Me_2EtN (7.6 mL, 70.2 mmol, 4.0 equiv) in THF (30 mL) at 0 °C overnight gave, after kugelrohl distillation 140 °C (3 mbar) {literature 60 °C, 0.3 mbar}; a yellow/orange oil; (1.19 g, 48% yield); ^1H NMR (300 MHz, $CDCl_3$) δ_H 1.15 (3H, t, *J* 7.4), 2.34 (2H, q, *J* 7.4), 3.79 (3H, s), 6.69-6.71 (1H, m), 6.81-6.83 (1H, m), 6.89 (1H, td, *J* 1.2, 7.5) and 6.95-6.99 (1H, m)

Synthesis of Precatalyst 7

The following series of reactions were carried out at AstraZeneca Process Research and Development facility in Macclesfield. All anhydrous solvents and chemicals were purchased from Aldrich and used without further purification. ^1H nuclear magnetic resonance (NMR) spectra were acquired on a Bruker Avance II 400 (400 MHz, ^1H) and Room temperature (rt) refers to 20-25 °C. Temperatures of 0 °C and −78 °C were obtained using ice/H_2O and CO_2(s)/acetone baths respectively.

(S6)-(*S*)-methyl 5-oxopyrrolidine-2-carboxylate

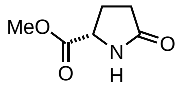

Following the procedure of O'Leary;[11] Amberlyst 15 wet resin (40g, 40% w/w) was added to a solution of (*S*)-pyroglutamic acid (100 g, 7.74 mol) in methanol (400 ml). The mixture was refluxed for 24 h, cooled, filtered and concentrated *in vacuo* to provide the product as a yellow oil with no purification necessary; (108 g, 97%); ^{1}H NMR (400 MHz, $CDCl_3$) δ_H 2.24-2.48 (4H, m), 3.77 (3H, s), 4.20-4.28 (1H, m) and 6.86 (1H, br s).

(S7)-(*S*)-5-(hydroxydiphenylmethyl)pyrrolidin-2-one

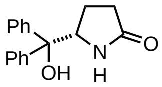

A solution of **S6** (46.0 g, 0.321, 1.0 equiv) in anhydrous THF (500 mL) in a 2 L four neck round bottom flask equipped with an overhead stirrer under a nitrogen atmosphere was cooled to −78 °C. Phenyl Lithium as a 1.8 M in Bu_2O (625 mL, 1.23 mol, 3.5 equiv) was added *via* cannula over 1 h as to maintain the reaction temperature below −50 °C. The solution was then allowed to warm to rt over 12 h before cooling to 0 °C. Saturated NH_4Cl(aq) (250 mL) was added dropwise, maintaining the reaction temperature <10 °C before a more rapid second addition of saturated NH_4Cl(aq) (250 mL). Following the second addition a thick white slurry formed that was directly filtered and dried in a vacuum oven to provide crude **S7**. Partial concentration of the organic filtrate and precipitation on standing for 12 h provided a second crop of crude **S7**. *Partial concentration must be carried out in a fume cupboard due to the large quantities of benzene generated. Full concentration was not attempted due to the high bp of Bu_2O.* The combined crude was heated to near reflux in EtOAc (500 mL) before the addition of the minimum quantity of methanol to allow dissolution (50 mL). The solution was cooled to 50 °C and held for 0.5 h at which point **S7** crystallised. The slurry was further cooled to 25 °C before filtration provided **S7**, *A second small crop was obtained via further cooling of the filtrate to 0 °C for 12 h*. (41.2g, 57% yield); mp 174-176 °C; ^{1}H NMR (400 MHz, $CDCl_3$) δ_H Data in accordance with the literature.[14] 1.82-1.91 (1H, m), 1.98-2.05 (1H, m), 2.12-2.20 (1H, m), 2.22-2.31 (1H, m), 4.61-4.62, (1H, m), 7.19-7.24 (2H, m), 7.26-7.30 (2H, m), 7.35-7.38 (2H, m), 7.45-7.48 (2H, m) and 7.48-7.51 (2H, m)

(S8)-(*S*)-5-(((tert-butyldimethylsilyl)oxy)diphenylmethyl)pyrrolidin-2-one

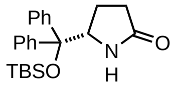

Following the procedure of Enders;[12] Tert-butyldimethylsilyl triflate (84.8 mL, 0.369 mol, 2.5 equiv) and 2,6-lutidine (51.3 mL, 0.443 mol, 3.0 equiv) were added dropwise to a solution of **S7** (39.5 g, 0.148 mol, 1.0 equiv) in CH_2Cl_2 (1 L) at 0 °C. After being stirred overnight at room temperature, the reaction mixture was quenched with 5% aqueous HCl (200 mL). The aqueous layer was extracted (CH_2Cl_2, 3 × 100 mL) and the organics combined, dried (Na_2SO_4) before concentration *in vacuo* to yield a crude **S3** as a white solid. The solid was then washed with hexane, dissolved in EtOAc and loaded onto a 10cm diameter sinter filter packed with 5cm of silica. This was eluted with a 1:1 mixture of petrol:EtOAc collecting the first 1 L, concentration in vacuo yielded pure **S8** as a colourless solid; (32.4 g, 57%); mp 150-152 °C; $[a]_D^{20}$ –46 (c 1.00, $CHCl_3$) {literature value[14] –65 (c 1.01, $CHCl_3$)}; ^{1}H NMR (400 MHz, $CDCl_3$) δ_H –0.39 (3H, s), –0.34 (3H, s), 0.95 (9H, s), 0.97-1.09 (1H, m), 1.82-1.90 (1H, m), 2.06-2.21 (2H, m), 4.64 (1H, dd, *J* 3.1, 8.5), 5.80 (1H, br s) and 7.30-7.36 (10H, m). Data in accordance with the literature.[14]

(7)-(*S*)-5-(((*t*-Butyldimethylsilyl)oxy)diphenylmethyl)-2-phenyl-6,7-dihydro-5H-pyrrolo[2,1-c][1,2,4]triazol-2-ium tetrafluoroborate

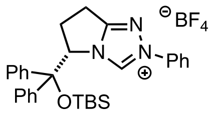

To an oven dried 250 mL four neck round bottom flask under nitrogen was added **S8** (20 g, 52.4 mmol, 1.0 equiv) and anhydrous CH_2Cl_2 (50 mL). To this solution was added trimethyloxonium tetrafluoroborate (8.49 g, 57.7 mmol, 1.1 equiv) and the solution stirred for 6 h (*the trimethyloxonium tetrafluoroborate does not instantly dissolve at this reaction concentraion*) after which NMR indicated the absence of starting material. Phenylhydrazine (5.70 mL, 57.7 mmol, 1.1 equiv) was added and the reaction mixture was stirred for 12 h. The solution was concentrated *in vacuo* to a pale yellow foam that was used in the next step without further purification. The crude was suspended in triethylorthoformate (50 mL) at 80 °C and the minimum quantity of methanol added to facilitate dissolution. The solution was heated for 6 h *(after 4 h the precipitation of* ***7*** *was seen)* before cooling to rt overnight. The precipitated precatalyst **7** was filtered and washed with Et_2O and cold EtOAc to provide crude **7**. This was then recrystallised in methanol (~150 mL) to provide (*S*)-**7** as a dark yellow/brown solid; (18.3 g, 61%); mp 182-186 °C; $[a]_D^{20}$–123 (c 1.00, MeCN) {literature value[13] –112 (c 0.5, MeCN)}; ^{1}H NMR (400 MHz, $CDCl_3$) δ_H –0.35 (3H, s), –0.32 (3H, s), 0.94 (9H, s), 1.61-1.70 (1H, m), 2.74-2.81 (1H, m), 2.86-2.93 (1H, m), 3.15-3.26 (1H, m), 6.12 (1H, dd, *J* 1.2, 8.9), 7.14 (2H, br s), 7.34 (2H, t, *J* 7.4), 7.40-7.47 (4H, m), 7.51, 7.57 (5H, m), 7.67-7.71 (2H, m), and 9.08 (1H, s). Data in accordance with the literature.[12]

Synthesis of Lactones and Chlorinated Products.

General procedure (1): Lactonisation and chlorination at 0 °C.

To a flame dried Schlenk flask under an argon atmosphere was added NHC precatalyst (0.10 mmol), base (0.09 mmol) and toluene (6 mL) and the mixture stirred for 15 min. The mixture was then cooled to 0 °C in an ice/H_2O bath followed by addition of a 0 °C solution of the requisite ketene (1.00 mmol) in toluene (12 mL), immediately followed by chloral (1.00 mmol). Toluene (2 mL) was added to wash residual reactants into solution and the reaction was stirred for the stated time at 0 °C before opening the flask to the air for 30 min and concentration *in vacuo*. The resulting crude residue with the stated diastereomeric ratio was purified by flash silica chromatography (ether:petrol) to provide either the isolated *anti* or *syn* diastereomer as stated.

General procedure (2): Lactonisation and chlorination at 0 °C with dropwise ketene addition.

In instances where ketene dimerization was competitive with lactonization or chlorination the ketene was added dropwise. To a flame dried Schlenk flask under an argon atmosphere was added NHC precatalyst (0.10 mmol), base (0.09 mmol) and toluene (6 mL) and the mixture stirred for 15 min. The mixture was then cooled to 0 °C in an ice/H_2O bath followed by addition of chloral (1.00 mmol). A 0 °C solution of the requisite ketene (1.00 mmol) in toluene (12 mL) was subsequently added over 0.5 h. The reaction was stirred for an additional 3 h at 0 °C before opening the flask to the air for 0.5 h and concentration *in vacuo*. The resulting crude residue with the stated diastereomeric ratio was purified by flash silica chromatography (ether:petrol) to provide either the isolated *anti* or *syn* diastereomer as stated.

(2)-*Anti*-(3*S*,4*R*)-3-ethyl-3-phenyl-4-(trichloromethyl)oxetan-2-one and (3)-*Syn*-(3*S*,4*R*)-3-ethyl-3-phenyl-4-(trichloromethyl)oxetan-2-one

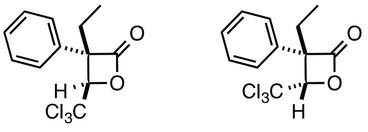

(2)-*anti* (3)-*syn*

Following general procedure **1**: NHC precatalyst **7** (28.5 mg, 0.05 mmol, 0.10 equiv), KHMDS (0.09 mL, 0.045 mmol, 0.09 equiv), ethylphenylketene (73.0 mg, 0.50 mmol, 1.00 equiv) and chloral (0.05 mL, 0.50 mmol, 1.0 equiv) in toluene (10 mL) at 0 °C for 3 h gave crude product (*dr* 72:28, *anti*:*syn*) that was purified by flash silica chromatography (2% ether:petrol) to give:

Anti (eluted first) as a colourless solid; (90 mg, 61% yield); mp 90-92 °C; $[a]_D^{20}$ +38.0 (c 0.65, MeOH); Chiral HPLC analysis; Chiralpak AS-H (3% IPA:hexane, flow rate 1 mL min^{-1}, 220 nm) t_R major (3*S*,4*R*): 4.7 min, t_R minor (3*R*,4*S*): 8.6 min, 92% *ee*; ν_{max} (KBr)/cm^{-1} 2986, 1829 (C=O), 1493, 1450, 1097, 832, 808 and 758; ^1H NMR (400 MHz, $CDCl_3$) δ_H 0.86 (3H, t, *J* 7.4), 2.04 (1H, dq, *J* 7.2, 14.0), 2.85 (1H, dq, *J* 7.2, 14.0), 5.06 (1H, s), 7.34-7.41 (1H, m) and 7.42-7.44 (4H, m); ^{13}C NMR (75

MHz $CDCl_3$): δ_C 8.9, 24.6, 69.9, 88.1, 95.6, 126.4, 128.3, 129.3, 137.0 and 168.9; *m/z*: (ESI+) 292.0 ($M(^{35}Cl))^+$ HRMS [M+]: found 291.9817; calcd 291.9819.

Syn (eluted second) as a colourless solid; (41 mg, 28% yield); mp 88-90 °C; $[a]_D^{20}$ +43 (c 1.1, MeOH); Chiral HPLC analysis; Chiralcel OJ-H (3% IPA:hexane, flow rate 1 mL min^{-1}, 220 nm) t_R major (3*S*,4*S*): 9.9 min, t_R minor (3*R*,4*R*): 12.0 min, 88% *ee*; ν_{max} (KBr)/cm^{-1} 2985, 1840 (C=O), 1497, 1450, 1099, 830 (CCl_3), 803 (CCl_3), 754 (CCl_3); ^1H NMR (400 MHz $CDCl_3$): δ_H 0.95 (3H, t, *J* 7.4), 2.32-2.47 (2H, m), 4.94 (1H, s), 7.33-7.35 (3H, m) and 7.46-7.48 (2H, m); ^{13}C NMR (100 MHz $CDCl_3$): δ_C 8.8, 33.2, 69.6, 86.9, 95.5, 128.2, 128.4, 128.3, 131.4, and 169.2; *m/z*: (ESI+) 292.0 ($M(^{35}Cl))^+$ HRMS [M+]: found 291.9819; calcd 291.9819.

(2)-*Anti*-(3*S*,4*R*)-3-ethyl-3-phenyl-4-(trichloromethyl)oxetan-2-one and (3)-*Syn*-(3*S*,4*S*)-3-ethyl-3-phenyl-4-(trichloromethyl)oxetan-2-one: Preparative scale

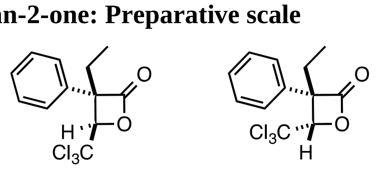

(2)-*anti* (3)-*syn*

Following general procedure **2**: NHC precatalyst **7** (214 mg, 0.38 mmol, 0.025 equiv), KHMDS (0.72 mL, 0.36 mmol, 0.024 equiv), ethylphenylketene (2.19 g, 15.0 mmol, 1.00 equiv) and chloral (1.46 mL, 15.0 mmol, 1.0 equiv) in toluene 300 mL at 0 °C for 3 h gave crude product (*dr* 74:26, *anti*:*syn*) that was purified by flash silica chromatography (2% ether:petrol)

Anti (eluted first) as a colourless solid; (2.40 g, 54% yield); mp 88-86 °C; $[a]_D^{20}$+54 (c 0.71, $CHCl_3$); Chiral HPLC analysis; Chiralpak AS-H (3% IPA:hexane, flow rate 1 mL min^{-1}, 220 nm) t_R major (3*S*,4*R*): 4.7 min, t_R minor (3*R*,4*S*): 9.7 min, 94% *ee*;

Syn (eluted second) as a colourless solid; (0.956 g, 22% yield); mp 74-78 °C; $[a]_D^{20}$+81 (c 0.51, $CHCl_3$); Chiral HPLC analysis; Chiralcel OJ-H (3% IPA:hexane, flow rate 1 mL min^{-1}, 220 nm) t_R major (3*S*,4*S*): 8.1 min, t_R minor (3*R*,4*R*): 11.6 min, 92% *ee*;

A portion of *anti* **2** and *syn* **3** were recrystallised from petrol/EtOAc to provide material of >99% *ee* for X-ray crystallographic analysis.

Anti $[a]_D^{20}$+54 (c 0.12, $CHCl_3$); Chiral HPLC analysis; Chiralpak AS-H (3% IPA:hexane, flow rate 1 mL min^{-1}, 220 nm) t_R major (3*S*,4*R*): 4.7 min, t_R minor (3*R*,4*S*): 8.6 min, >99% *ee*; *Syn* $[a]_D^{20}$+118 (c 0.17, $CHCl_3$); Chiral HPLC analysis; Chiralcel OJ-H (3% IPA:hexane, flow rate 1 mL min^{-1}, 220 nm) t_R major (3*S*,4*S*): 10.0 min, t_R minor (3*R*,4*R*): 13.5 min, >99% *ee*;

(5)-(*S*)-2,2-Dichlorovinyl 2-chloro-2-(naphthalene-1-yl)butanoate

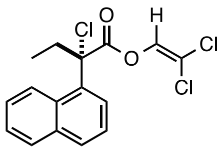

(5)

Following general procedure **1**: NHC precatalyst **7** (57.0 mg, 0.10 mmol, 0.10 equiv), KHMDS (0.18 mL, 0.09 mmol, 0.09 equiv), ethyl-1-napthylketene **4** (196 mg, 1.00 mmol, 1.0 equiv) and chloral (0.10 mL, 1.00 mmol, 1.0 equiv) in toluene (20 mL) at 0 °C for 12 h gave crude product that was purified by flash silica chromatography (0.5% ether:petrol) to give **5** as a colorless oil; (273 mg, 80% yield); $[a]_D^{20}$+ 96 (c 0.75, $CHCl_3$); Chiral HPLC analysis; Chiralcel OJ-H (0.5% IPA:hexane, flow rate 1 mL min^{-1}, 220 nm) t_R minor (*R*): 9.2 min, t_R major (*S*): 10.5 min, 92% *ee*; ν_{max} (thin film)/cm^{-1} 3093, 2981, 1762 (C=O), 1650, 1601, 1512, 1459, 1400, 1345, 1286, 1203, 1124, 986, 804, 775, 652 and 619; ^{1}H NMR (300 MHz, $CDCl_3$) δ_H 1.06 (3H, t, *J* 7.3), 2.73-2.85 (2H, m), 7.47-7.56 (3H, m), overlapping 7.49 (1H, s) 7.87-7.92 (3H, m) and (1H, dd, *J* 3.1, 6.4); ^{13}C NMR (75 MHz $CDCl_3$): δ_C 9.0, 33.5, 74.1, 114.9, 123.5, 124.8, 125.3, 125.8, 126.9, 129.6, 130.1, 130.4, 133.1, 133.5, 134.3 and 167.6; *m/z*: (CI+) (M(^{35}Cl))$^+$ HRMS $C_{15}H_{11}O_2Cl_3$ [M+NH4]$^+$: found 360.0325; calcd 360.0319.

(5)-(*S*)-2,2-Dichlorovinyl 2-chloro-2-(naphthalene-1-yl)butanoate: Preparative scale

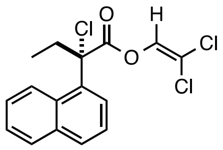

(5)

Following general procedure **1**: NHC precatalyst **7** (51.0 mg, 0.09 mmol, 0.025 equiv), KHMDS (0.17 mL, 0.09 mmol, 0.024 equiv), ethyl-1-napthylketene **5** (702 mg, 3.58 mmol, 1.0 equiv) and chloral (0.35 mL, 3.58 mmol, 1.0 equiv) in toluene (90 mL) at 0 °C for 3 h gave crude product that was purified by flash silica chromatography (0.5% ether:petrol) to give **5** as a pale yellow oil; (1.05 g, 86% yield); $[a]_D^{20}$ + 114 (c 0.55, $CHCl_3$); Chiral HPLC analysis; Chiralcel OJ-H (0.5% IPA:hexane, flow rate 1 mL min^{-1}, 220 nm) t_R minor (*R*): 10.2 min, t_R major (*S*): 12.0 min, 94% *ee*.

Assignment of the absolute configuration of 5: (*S*)-2-chloro-2-(naphthalen-1-yl)-*N*-((*S*)-1-phenylethyl)butanamide

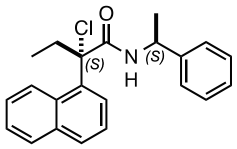

(6)

To a solution of **5** (60.0 mg, 0.17 mmol, 1.0 equiv, 94% *ee*) in CH_2Cl_2 (3 mL) at 0 °C, was added Br_2 (9 μL, 0.17 mmol, 1.0 equiv) and the solution warmed to rt and stirred for 1.5 h. The volatiles were removed *in vacuo* and the crude material dissolved in in THF (3 mL). To this solution was added

(*S*)-(−)-α-methylbenzylamine (0.11 mL, 0.87 mmol, 5.0 equiv) and the resulting light blue solution stirred for 16 h at rt. The solution was diluted with EtOAc and washed with 2 × 1M HCl, dried Na_2SO_4 and concentrated *in vacuo* to give crude material that was purified by flash silica chromatography (5% to 10% ether:petrol) to give a **6** as a pale yellow solid; (55 mg, 90% yield); mp 98-100 °C; $[a]_D^{20}$ + 110 (c 0.15, $CHCl_3$); ν_{max} (ATR)/cm^{-1} 3298, 2926, 1645 (C=O), 1510, 1450, 1234, 1132, 1022 and 910 ^{1}H NMR (300 MHz, $CDCl_3$) δ_H 0.94 (3H, t, *J* 7.2), 1.38 (3H, d, *J* 6.9), 2.67 (1H, dq, *J* 7.2, 14.3), 2.76 (1H, dq, *J* 7.2, 14.3), 5.16 (1H, *app* quintet, *J* 7.2), 6.44 (1H, d, *J* 7.8), 7.23-7.27 (3H,m), 7.29-7.33 (2H, m), 7.47-7.52 (3H, m), 7.85-7.91 (3H, m) and 8.14-8.17 (1H, m); ^{13}C NMR (100 MHz $CDCl_3$): δ_C 9.4, 34.3, 49.9, 77.2, 125.1, 125.2, 126.1, 126.2, 126.5, 126.6, 126.7, 128.9, 129.6, 130.5, 130.8, 134.8, 136.1, 143.1 and 169.9; *m/z*: (ESI+) (M(^{35}Cl))$^+$ HRMS $C_{22}H_{23}ONCl$ [M+H]$^+$: found 352.1467; calcd 352.1463.

(8)-*Anti*-(3*S*,4*R*)-3-(4-methoxyphenyl)-3-ethyl-4-(trichloromethyl)oxetan-2-one –and (8)-*Syn*-(3*S*,4*S*)-3-(4-methoxyphenyl)-3-ethyl-4-(trichloromethyl)oxetan-2-one

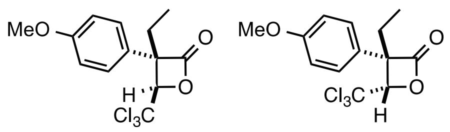

(8)-*anti* (8)-*syn*

Following general procedure **1**: NHC precatalyst **7** (57 mg, 0.10 mmol, 0.10 equiv), KHMDS (0.18 mL, 0.09 mmol, 0.09 equiv), ethyl-4-methoxyphenylketene (176 mg, 1.00 mmol, 1.0 equiv) and chloral (0.10 mL, 1.00 mmol, 1.0 equiv) in toluene (20 mL) at 0 °C for 3 h gave crude product (*dr* 75:25, *anti*:*syn*) that was purified by flash silica chromatography (5% ether:petrol)

Anti (eluted first) as a colourless solid; (172 mg, 53% yield); mp 62-64 °C; $[a]_D^{20}$ +48 (c 0.50, $CHCl_3$); Chiral HPLC analysis; Chiralpak AS-H (3% IPA:hexane, flow rate 1 mL min^{-1}, 220 nm) t_R major (3*S*,4*R*): 4.6 min, t_R minor (3*R*,4*S*): 9.8 min, 88% *ee*; ν_{max} (KBr)/cm^{-1} 3055, 2987, 1836 (C=O), 1514, 1185, 1095, 1033, 951, 929, 896 and 834; ^{1}H NMR (300 MHz, $CDCl_3$) δ_H 0.86 (3H, t, *J* 7.4, CH_2CH_3), 2.00 (1H, dq, *J* 7.5, 13.8, $CH_AH_BCH_3$), 2.82 (1H, dq, *J* 7.1, 13.8, $CH_AH_BCH_3$), 3.83 (3H, s, OCH_3), 5.02 (1H, s, $CHCCl_3$), 6.93-6.96 (2H, m, Ar*H*-3,5) and 7.32-7.35 (2H, m, Ar*H*-2,6); ^{13}C NMR (75 MHz $CDCl_3$): δ_C 8.9 (CH_3), 24.6 (CH_2), 55.4, ($ArOCH_3$) 69.4 (*C*(Ar)Et), 88.4 (*C*H(CCl_3)), 95.7 (CCl_3), 114.6 (Ar*C*-1), 127.6 (Ar*C*-3,5), 129.1 (Ar*C*-2,6), 159.5 (Ar*C*-4) and 169.2 (*C*=O); *m/z*: (CI+) (M(^{35}Cl))$^+$ HRMS $C_{13}H_{17}O_3N_1Cl_3$ [M+NH_4]$^+$: found 340.0269; calcd 340.0269.

Syn (eluted second) as a colourless solid; (54 mg, 17% yield); mp 82-84 °C; $[a]_D^{20}$ +90 (c 0.50, $CHCl_3$); Chiral HPLC analysis; Chiralcel OJ-H (1% IPA:hexane, flow rate 1 mL min^{-1}, 220 nm) t_R major (3*S*,4*S*): 16.1 min, t_R minor (3*R*,4*R*): 20.8 min, 84% *ee*; ν_{max} (KBr)/cm^{-1} 2925, 1836 (C=O), 1612, 1516, 1254, 1186, 1093, 1035, 924 and 832; ^{1}H NMR (300 MHz $CDCl_3$): δ_H 0.94 (3H, t, *J* 7.4), 2.28-2.43 (2H, m), 3.81 (3H, s), 4.90 (1H, s), 6.86 (2H, d, *J* 9.0) and 7.36 (2H, d, *J* 9.0); ^{13}C NMR (75 MHz $CDCl_3$): δ_C 8.8, 33.1, 55.3, 69.2, 86.9, 95.7, 113.6, 123.0, 129.9, 159.5 and 169.5; *m/z*: (EI+) (M(^{35}Cl))$^+$ HRMS $C_{13}H_{13}O_3Cl_3$ [M+]: found 321.9923; calcd 321.9925.

(9)-*Anti*-(3*S*,4*R*)-3-(4-fluorophenyl)-3-ethyl-4-(trichloromethyl)oxetan-2-one and (9)-*Syn*-(3*S*,4*S*)-3-(4-fluorophenyl)-3-ethyl-4-(trichloromethyl)oxetan-2-one and

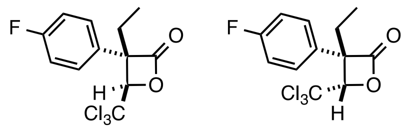

(9)-*anti* (9)-*syn*

Following general procedure **2**: NHC precatalyst **7** (28.0 mg, 0.05 mmol, 0.10 equiv), KHMDS (0.09 mL, 0.05 mmol, 0.09 equiv), ethyl-4-fluorophenylketene (82.1 mg, 0.50 mmol, 1.0 equiv) and chloral (0.05 mL, 0.05 mmol, 1.0 equiv) in toluene (10 mL) at 0 °C for 3 h gave crude product (*dr* 69:31, *anti*:*syn*) that was purified by flash silica chromatography (2% ether:petrol)

Anti (eluted first) as a colourless solid; (80 mg, 51% yield); mp 85-86 °C; $[\alpha]_D^{20}$ +58 (c 0.5, $CHCl_3$); Chiral HPLC analysis; Chiralpak AS-H (0.5 % IPA:hexane, flow rate 1 mL min^{-1}, 220 nm) t_R major (3*S*,4*R*): 5.6 min, t_R minor (3*R*,4*S*): 13.4 min, 94% *ee*. ν_{max} (KBr)/cm^{-1} 2979, 2938, 1840 (C=O), 1606, 1514, 1229, 1166, 1098, 912, 840, 829, 820, 798, 759, 660, 643 and 535; ^1H NMR (300 MHz, $CDCl_3$) δ_H 0.86 (3H, t, *J* 7.4), 2.01 (1H, dq, *J* 7.5, 13.8), 2.86 (1H, dq, *J* 7.1, 13.8), 5.03 (1H, s), 7.10-7.18 (2H, m) and 7.39-7.44 (2H, m); ^{13}C NMR (75 MHz $CDCl_3$): δ_C 8.9, 24.6, 69.3, 88.1, 95.4, 116.3 (d, *J* 22.1), 128.2 (d, *J* 8.2), 132.8 (d, *J* 3.3) 162.5 (d, *J* 248.2) and 168.7; *m/z*: (EI+) $(M(^{35}Cl))^+$ HRMS, $C_{12}H_{10}O_2Cl_3F_1$ [M+]: found 309.9724; calcd 309.9725.

Syn (eluted second) as a colourless solid; (35 mg, 22% yield); mp 90-92 °C; $[\alpha]_D^{20}$ +128 (c 0.05, $CHCl_3$); Chiral HPLC analysis; Chiralpak AS-H (1% IPA:hexane, flow rate 1.0 mL min^{-1}, 220 nm) t_R minor (3*R*,4*R*): 6.7 min, t_R major (3*S*,4*S*): 8.0 min, 97% *ee*; ν_{max} (KBr)/cm^{-1} 2979, 2937, 1840 (C=O), 1606, 1514, 1465, 1229, 1166, 1143, 1098, 1015, 912, 930, 840, 829, 819, 798, 759, 727, 760, 660, 643 and 535; ^1H NMR (500 MHz $CDCl_3$): δ_H 0.96 (3H, t, *J* 7.4), 2.32-2.44 (2H, m), 4.93 (1H, s), 7.02-7.08 (2H, m) and 7.43-7.48 (2H, m); ^{13}C NMR (100 MHz $CDCl_3$): δ_C 8.7, 33.2, 68.9, 86.8, 95.4, 115.3 (d, *J* 22.1), 127.2 (d, *J* 3.3), 130.4 (d, *J* 8.2), 162.5 (d, *J* 248.6) and 169.0; *m/z*: (EI+) $(M(^{35}Cl))^+$ HRMS $C_{12}H_{10}O_2Cl_3F_1$ [M+]: found 309.9723; calcd 309.9725.

(10)-*Anti*-(3*S*,4*R*)-3-(4-chlorophenyl)-3-ethyl-4-(trichloromethyl)oxetan-2-one and (10)-*Syn*-(3*S*,4*S*)-3-(4-chlorophenyl)-3-ethyl-4-(trichloromethyl)oxetan-2-one

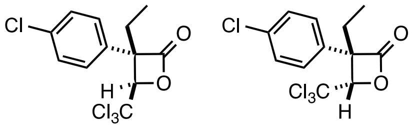

(10)-*anti* (10)-*syn*

Following general procedure **2**: NHC precatalyst **7** (57.0 mg, 0.10 mmol, 0.10 equiv), KHMDS (0.18 mL, 0.09 mmol, 0.09 equiv), ethyl-4-chlorophenylketene (181 mg, 1.00 mmol, 1.0 equiv) and chloral (0.10 mL, 1.00 mmol, 1.0 equiv) in toluene (20 mL) at 0 °C for 3 h gave crude product (*dr* 74:26, *anti*:*syn*) that was purified by flash silica chromatography (2% ether:petrol)

Anti (eluted first) as a colourless solid; (220 mg, 67% yield); mp 78-81 °C; $[\alpha]_D^{20}$ +50 (c 0.50, $CHCl_3$); Chiral HPLC analysis; Chiralpak AS-H (3% IPA:hexane, flow rate 1 mL min^{-1}, 220 nm) t_R

major (3*S*,4*R*): 5.6 min, t_R minor (3*R*,4*S*): 9.3 min, 92% *ee*; ν_{max} (KBr)/cm^{-1} 2919, 1837 (C=O), 1713, 1494, 1261, 1095, 1016, 831, 804 and 761; ^{1}H NMR (300 MHz, $CDCl_3$) δ_H 0.86 (3H, t, *J* 7.2, CH_2CH_3), 2.02 (1H, dq, *J* 7.2, 14.1, $CH_AH_BCH_3$), 2.87 (1H, dq, *J* 7.2, 14.1, $CH_AH_BCH_3$), 5.02 (1H, s, C*H*CCl_3) and 7.36-7.44 (4H, m, Ar*H*); ^{13}C NMR (100 MHz $CDCl_3$): δ_C 9.0 (*C*H_3), 24.6 (*C*H_2), 69.5 (*C*(Ar)Et), 88.0 (*C*H(CCl_3)), 95.4 (*C*Cl_3), 127.9 (Ar*C*H), 129.6 (Ar*C*H), 134.6 (Ar*C*-4), 135.5 (Ar*C*-1) and 168.6 (C=O); *m/z*: (CI+) (M(^{35}Cl))$^+$ HRMS $C_{12}H_{14}O_2N_1Cl_4$ [M+NH_4]$^+$: found 343.9771; calcd 343.9773.

Syn (eluted second) as a colourless solid; (69 mg, 21% yield); mp 118-120 °C; $[a]_D^{20}$ +94 (c 0.13, $CHCl_3$); Chiral HPLC analysis; Chiralpak AS-H (3% IPA:hexane, flow rate 0.5 mL min^{-1}, 220 nm) t_R major (3*S*,4*S*): 17.1 min, t_R minor (3*R*,4*R*): 20.7 min, 84% *ee*; ν_{max} (KBr)/cm^{-1} 3055, 2986, 1849 (C=O), 1517, 1451, 1422, 1096, 926, 896, 838 and 819; ^{1}H NMR (300 MHz $CDCl_3$): δ_H 0.96 (3H, t, *J* 7.4), 2.34-2.41 (2H, m), 4.94 (1H, s), 7.32-7.36 (2H, m) and 7.40-7.43 (2H, m); ^{13}C NMR (100 MHz $CDCl_3$): δ_C 8.7, 33.1, 69.0, 86.8, 95.4, 128.5, 129.9, 130.0, 134.6 and 168.8; *m/z*: (CI+) (M(^{35}Cl))$^+$ HRMS $C_{12}H_{14}O_2N_1Cl_4$ [M+]: found 343.9769; calcd 343.9773.

(11)-*Anti*-(3*S*,4*R*)-3-(4-bromophenyl)-3-ethyl-4-(trichloromethyl)oxetan-2-one and (11)-*Syn*-(3*S*,4*S*)-3-(4-bromophenyl)-3-ethyl-4-(trichloromethyl)oxetan-2-one and

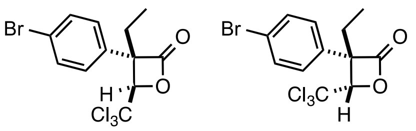

(11)-*anti* (11)-*syn*

Following general procedure **2**: NHC precatalyst **7** (28.0 mg, 0.05 mmol, 0.10 equiv), KHMDS (0.09 mL, 0.05 mmol, 0.09 equiv), ethyl-4-bromophrnylketene (113 mg, 0.50 mmol, 1.0 equiv) and chloral (0.05 mL, 0.05 mmol, 1.0 equiv) in toluene (10 mL) at 0 °C for 3 h gave crude product (*dr* 75:25, *anti*:*syn*) that was purified by flash silica chromatography (1% ether:petrol)

Anti (eluted first) as a colourless solid; (115 mg, 62% yield); mp 76-78 °C; $[a]_D^{20}$ +31 (c 0.25, $CHCl_3$); Chiral HPLC analysis; Chiralpak AS-H (1% IPA:hexane, flow rate 1 mL min^{-1}, 220 nm) t_R major (3*S*,4*R*): 6.1 min, t_R minor (3*R*,4*S*): 11.6 min, 90% *ee*; ν_{max} (KBr)/cm^{-1} 2971, 1831 (C=O), 1489, 1399, 1261, 1102, 1012, 954, 930, 834, 803, 757, 735, 716, 656 and 526; ^{1}H NMR (400 MHz, $CDCl_3$) δ_H 0.86 (3H, t, *J* 7.4), 2.01 (1H, dq, *J* 7.2, 14.1), 2.86 (1H, dq, *J* 7.1, 14.1), 5.01 (1H, s), 7.30-7.33 (2H, m) and 7.56-7.59 (2H, m); ^{13}C NMR (75 MHz $CDCl_3$): δ_C 7.8, 23.3, 68.4, 86.8, 94.2, 121.6, 127.0, 131.4, 134.9 and 167.4; *m/z*: (EI+) (M(^{35}Cl)(Br^{79}))$^+$ HRMS $C_{12}H_{10}O_2Br_1Cl_3$ [M+]: found 369.8920; calcd 369.8924.

Syn (eluted second) as a colourless solid; (37 mg, 17% yield); mp 110-112 °C; $[a]_D^{20}$ +86 (c 0.07, $CHCl_3$); Chiral HPLC analysis; Chiralpak AS-H (1% IPA:hexane, flow rate 1.0 mL min^{-1}, 220 nm) t_R major (3*S*,4*S*): 10.0 min, t_R minor (3*R*,4*R*): 11.2 min, 82% *ee*; ν_{max} (KBr)/cm^{-1} 2968, 1838 (C=O), 1493, 1452, 1399, 1254, 1146, 1099, 1016, 911, 930, 832, 820, 800, 762, 735, 721, 647 and 525; ^{1}H NMR (500 MHz $CDCl_3$): δ_H 0.95 (3H, t, *J* 7.3), 2.34-2.38 (2H, m), 4.93 (1H, s), 7.35 (2H, d, *J* 8.2) and 7.48

(2H, d, *J* 8.2); ^{13}C NMR (100 MHz $CDCl_3$): δ_C 9.1, 33.5, 69.4, 87.2, 95.7, 123.2, 130.6, 131.0, 131.9 and 169.2; *m/z*: (EI+) (M(^{35}Cl)(Br^{79}))$^+$ HRMS $C_{12}H_{10}O_2Br_1Cl_3$ [M+]: found 369.8926; calcd 369.8924.

(12)-*Anti*-(3*S*,4*R*)-3-methyl-3-phenyl-4-(trichloromethyl)oxetan-2-one and (12)-*Syn*-(3*S*,4*S*)-3-methyl-3-phenyl-4-(trichloromethyl)oxetan-2-one

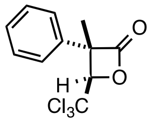

(12)-*anti*

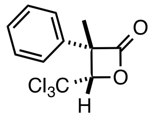

(12)-*syn*

To a flame dried Schlenk flask under an argon atmosphere was added NHC precatalyst **7** (57.0 mg, 0.10 mmol, 0.10 equiv), KHMDS (0.18 mL, 0.09 mmol, 0.09 equiv) and toluene (6 mL) and the mixture stirred for 15 min. The mixture was then cooled to 0 °C in an ice/H_2O bath followed by addition of chloral (0.10 mL, 1.00 mmol, 1.0 equiv). A 0 °C solution of the methylphenylketene (132 mg, 1.00 mmol, 1.0 equiv) in toluene (14 mL) was subsequently added dropwise over 0.5 h. The reaction was stirred for an additional 3 h at 0 °C before opening the flask to the air for 30 min. The solution was then passed through a plug of silica, washed with toluene and concentrated *in vacuo* to yield the combined *syn* and *anti* diastereomers (*dr* 88:12, *anti*:*syn*) as a colourless solid; (265 mg, 95% yield):

Anti; mp 68–72 °C; $[a]_D^{20}$ +21 (c 0.15, $CHCl_3$); Chiral HPLC analysis; Chiralpak AS-H (3% IPA:Hexane, flow rate 1 mL min^{-1}, 220 nm) t_R major (3*S*,4*R*): 5.4 min, t_R minor (3*R*,4*S*): 11.4 min, 82% *ee*; ν_{max} (KBr)/cm^{-1} 3060, 1846 (C=O), 1266, 1086, 939 and 740; ^{1}H NMR (300 MHz, $CDCl_3$) δ_H 1.89 (3H, s), 5.16 (1H, s) and 7.36-7.46 (5H, m); ^{13}C NMR (75 MHz $CDCl_3$): δ_C 19.4, 65.5, 87.2, 95.6, 125.4, 128.4, 129.5, 139.5 and 169.7; *m/z*: (CI+) (M(^{35}Cl))$^+$ HRMS $C_{11}H_{13}O_2NCl_3$ $[M+NH_4]^+$: found 296.0006; calcd 296.0003.

Syn; Chiral HPLC analysis; Chiralpak AS-H (1% IPA:hexane, flow rate 1 mL min^{-1}, 220 nm) t_R major (3*S*,4*S*): 16.6 min, t_R minor (3*R*,4*R*): 20.2 min, 78% *ee*; ^{1}H NMR (300 MHz, $CDCl_3$, Characteristic signals) δ_H 2.00 (3H, s) and 4.98 (1H, s); ^{13}C NMR (75 MHz $CDCl_3$, Characteristic signals): δ_C 67.1, 95.34, 128.2, 128.3, 128.5, 132.8 and 169.9.

(13)-*Anti*-(3*S*,4*R*)-3-butyl-3-phenyl-4-(trichloromethyl)oxetan-2-one and (13)-*Syn*-(3*S*,4*S*)-3-butyl-3-phenyl-4-(trichloromethyl)oxetan-2-one

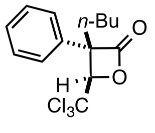

(13)-*anti*

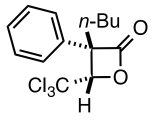

(13)-*syn*

Following general procedure **1**: NHC precatalyst **7** (57.0 mg, 0.10 mmol, 0.10 equiv), KHMDS (0.18 mL, 0.09 mmol, 0.09 equiv), butylphenylketene (174 mg, 1.00 mmol, 1.0 equiv) and chloral (0.10 mL, 1.00 mmol, 1.0 equiv) in toluene (20 mL) at rt for 3 h gave crude product (*dr* 69:31, *anti*:*syn*) that was

purified by flash silica chromatography (2% ether:petrol) to give a mixture of *anti* and *syn* (240 mg, 75% yield);

Anti (eluted first) as a colourless solid; mp 58 °C; $[a]_D^{20}$ +42 (c 0.45, $CHCl_3$); Chiral HPLC analysis; Chiralpak AS-H (3% IPA:hexane, flow rate 1 mL min^{-1}, 220 nm) t_R major (3*S*,4*R*): 4.6 min, t_R minor (3*R*,4*S*): 8.9 min, 88% *ee*. ν_{max} (thin film)/cm^{-1} 3055, 1841 (C=O), 1099, 937, 896 and 833; ^{1}H NMR (400 MHz, $CDCl_3$) δ_H 0.80 (3H, t, *J* 7.3), 0.85-0.98 (1H, m), 1.15-1.26 (2H, m), 1.43-1.52 (1H, m), 2.01 (1H, td, *J* 4.7, 13.2), 2.73 (1H, ddd, *J* 4.3, 12.2, 13.2), 5.03 (1H, s) and 7.36-7.45 (5H, m); ^{13}C NMR (75 MHz $CDCl_3$): δ_C 13.7, 22.8, 26.4, 31.0, 69.5, 88.1, 95.6, 126.2, 128.3, 129.2, 137.5 and 169.0; *m/z*: (CI+) (M(^{35}Cl))$^+$ HRMS $C_{14}H_{19}O_2NCl_3$ [M+NH_4]$^+$: found 338.0475; calcd 338.0476.

Syn ^{1}H NMR (400 MHz $CDCl_3$, Characteristic signals): δ_H 4.77 (1H, s, *CH*CCl_3) ^{13}C NMR (100 MHz $CDCl_3$, Characteristic signals): δ_C 13.9, 22.7, 26.4, 69.3, 87.2 and 169.4.

(14)-*Anti*-(3*S*,4*R*)-3-isobutyl-3-phenyl-4-(trichloromethyl)oxetan-2-one and (14)-*Syn*-(3*S*,4*R*)-3-isobutyl-3-phenyl-4-(trichloromethyl)oxetan-2-one

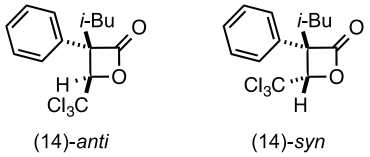

Following general procedure **1**: NHC precatalyst **7** (57.0 mg, 0.10 mmol, 0.1 equiv), KHMDS (0.18 mL, 0.09 mmol, 0.09 equiv), *i*-butylphenylketene (174 mg, 1.00 mmol, 1.0 equiv) and chloral (0.10 mL, 1.00 mmol, 1.0 equiv) in toluene (20 mL) at 0 °C for 3 h gave crude product (*dr* 42:58, *anti*:*syn*) that was purified by flash silica chromatography (1% ether:petrol) to give a partially separable mixture of *anti* and *syn* diastereomers (274 mg, 85% yield);

Anti (eluted first) as a colourless solid; mp 86 °C, $[a]_D^{20}$+13 (c 0.3, $CHCl_3$); ν_{max} (KBr)/cm^{-1} 2961, 1834 (C=O), 1492, 1449, 1095, 933, 848, 825, 782, 727 and 701; ^{1}H NMR (300 MHz, $CDCl_3$) δ_H 0.65 (3H, t, *J* 6.7), 0.96 (3H, t, *J* 6.7), 1.45-1.51 (1H, m), 2.08 (1H, dd, *J* 4.3, 14.1), 2.55 (1H, dd, *J* 7.9, 14.1), 4.95 (1H, s) and 7.34-7.47 (5H, m); ^{13}C NMR (100 MHz $CDCl_3$): δ_C 23.2, 24.6, 24.8, 39.7, 69.9, 88.2, 95.8, 126.6, 128.6, 129.5, 137.9 and 168.9; *m/z*: (ASAP) (M(^{35}Cl))$^+$ HRMS $C_{14}H_{19}O_2NCl_3$ [M+NH_4]$^+$: found 338.0476; calcd 338.0476.

Syn (eluted second) as a colourless solid: mp 78 °C; $[a]_D^{20}$ +90 (c 0.05, $CHCl_3$); Chiral HPLC analysis; Chiralcel OJ-H (3% IPA:hexane, flow rate 0.2 mL min^{-1}, 220 nm) t_R major (*S*,*S*): 30.8 min, t_R minor (*S*,*R*): 39.1 min, 84% *ee*; ν_{max} (KBr)/cm^{-1} 2956, 1840 (C=O), 1496, 1450, 1096, 930, 804, 762 and 701; ^{1}H NMR (300 MHz $CDCl_3$): δ_H 0.75 (3H, t, *J* 6.7), 0.94 (3H, t, *J* 6.7), 1.57-1.65 (1H, m), 2.13 (1H, dd, *J* 4.3, 14.1), 2.45 (1H, dd, *J* 7.9, 14.1), 4.87 (1H, s), 7.32-7.36 (3H, m) and 7.47-7.51 (2H, m); ^{13}C NMR (100 MHz $CDCl_3$): δ_C 23.3, 24.1, 25.0, 48.7, 69.2, 87.8, 95.7, 128.4, 128.6, 128.9, 131.5 and 169.4; *m/z*: (ESI+) (M(^{35}Cl))$^+$ HRMS $C_{14}H_{15}O_2Cl_3$ [M+]: found 320.0130; calcd 320.0132.

(16)-(*S*)-2,2-Dichlorovinyl 2-chloro-3-methyl-2-phenylbutanoate

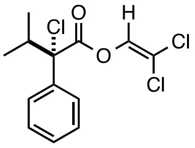

(16)

Following general procedure **1**: NHC precatalyst **7** (57.0 mg, 0.10 mmol, 0.10 equiv), KHMDS (0.18 mL, 0.09 mmol, 0.09 equiv), *i*-propoylphenylketene (160 mg, 1.00 mmol, 1.0 equiv) and chloral (0.10 mL, 1.00 mmol, 1.0 equiv) in toluene (20 mL) at 0 °C for 12 h gave crude product that was purified by flash silica chromatography (0.5% ether:petrol) to give **16** as a colorless oil; (266 mg, 86% yield); $[a]_D^{20}$ – 6 (c 1.0, $CHCl_3$); Chiral HPLC analysis; Chiralcel OJ-H (1% IPA:hexane, flow rate 1 mL min^{-1}, 220 nm) t_R major (*S*): 5.5 min, t_R minor (*R*): 8.4 min, 88% *ee*; ν_{max} (thin film)/cm^{-1}3094, 2977, 1755 (C=O), 1494, 1447, 1390, 1282, 1205, 1126, 1008, 867, 764 and 695; ^1H NMR (300 MHz, $CDCl_3$) δ_H 0.84 (3H, d, *J* 6.5), 1.15 (3H, d, *J* 6.5), 2.98-3.11 (1H, m), 7.31-7.41 (3H, m), 7.51 (1H, s) and 7.63-7.66 (2H, m); ^{13}C NMR (75 MHz $CDCl_3$): δ_C 17.0, 18.8, 37.1, 80.8, 114.3, 126.9, 128.5, 128.7, 133.2, 137.1 and 166.0; *m/z*: (ASAP) $(M(^{35}Cl))^+$ HRMS $C_{13}H_{17}O_2NCl_3$ $[M+NH_4]^+$: found 324.0323; calcd 324.0319.

(17)-(*S*)-2,2-Dichlorovinyl 2-chloro-2-(2-tolyl)propanoate

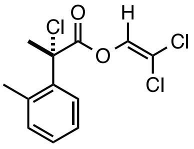

(17)

Following general procedure **1**: NHC precatalyst **7** (57.0 mg, 0.10 mmol, 0.10 equiv), KHMDS (0.18 mL, 0.09 mmol, 0.09 equiv), methyl-2-tolylketene (146 mg, 1.00 mmol, 1.0 equiv) and chloral (0.10 mL, 1.00 mmol, 1.0 equiv) in toluene (20 mL) at 0 °C for 12 h gave crude product that was purified by flash silica chromatography (1% ether:petrol) to give **17** as a colorless oil; (152 mg, 52% yield); $[a]_D^{20}$ +51 (c 0.35, $CHCl_3$); Chiral HPLC analysis; Chiralcel OJ-H (1% IPA:hexane, flow rate 1 mL min^{-1}, 220 nm) t_R minor (*R*): 7.4 min, t_R major (*S*): 8.4 min, 80% *ee*; ν_{max} (thin film)/cm^{-1} 3091, 2977, 1772 (C=O), 1601, 1587, 1489, 1464, 1437, 1283, 1247, 1195, 1135, 1125, 1089, 1026, 992, 906, 863 and 753; ^1H NMR (500 MHz, $CDCl_3$) δ_H 2.22 (3H, s), 2.30 (3H, s), 7.17-7.19 (1H, m), 7.23-7.28 (2H, m), 7.56 (1H, s), and 762-7.63 (1H, m); ^{13}C NMR (125 MHz $CDCl_3$): δ_C 20.3, 29.6, 68.9, 114.9, 125.7, 126.2, 129.1, 132.2, 133.2, 136.0, 137.5 and 167.7; *m/z*: (EI+) $(M(^{35}Cl))^+$ HRMS $C_{12}H_{14}O_2N_1Cl_4$ [M+]: found 291.9816; calcd 291.9819.

(18)-(*S*)-2,2-Dichlorovinyl 2-chloro-2-(2-tolyl)butanoate

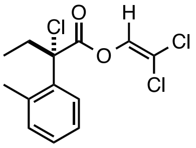

(18)

Following general procedure **1**: NHC precatalyst **7** (57.0 mg, 0.10 mmol, 0.10 equiv), KHMDS (0.18 mL, 0.09 mmol, 0.09 equiv), ethyl-2-tolylketene (160 mg, 1.00 mmol, 1.0 equiv) and chloral (0.10 mL, 1.00 mmol, 1.0 equiv) in toluene (20 mL) at 0 °C for 12 h gave crude product that was purified by flash silica chromatography (1% ether:petrol) to give **18** as a colorless oil; (219 mg, 71% yield); $[a]_D^{20}$ +130 (c 0.05, $CHCl_3$); Chiral HPLC analysis; Chiralcel OJ-H (0.5% IPA:hexane, flow rate 0.2 mL min^{-1}, 220 nm) t_R minor (*R*): 33.4 min, t_R major (*S*): 37.2 min, 82% *ee*; ν_{max} (thin film)/cm^{-1} 3093, 2980, 1768 (C=O), 1460, 1286, 1199, 1122, 991, 908, 873, 857, 751 and 737; ^1H NMR (400 MHz, $CDCl_3$) δ_H 1.02 (3H, t, *J* 7.3), 2.29 (3H, s), 2.48-2.65 (2H, m), 7.17-7.19 (1H, m), 7.24-7.27 (2H, m), 7.57 (1H, s), and 762-7.64 (1H, m); ^{13}C NMR (75 MHz $CDCl_3$): δ_C 8.9, 20.4, 33.3, 74.1, 114.8, 126.1, 126.6, 128.8, 132.2, 133.1, 135.8, 136.6 and 167.0; *m/z*: (CI+) $(M(^{35}Cl))^+$ HRMS $C_{13}H_{17}O_2N_1Cl_3$ $[M+NH_4]^+$: found 324.0324; calcd 324.0319.

(19)-(*S*)-2,2-Dichlorovinyl 2-chloro-2-(naphthalene-1-yl)propanoate

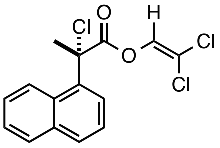

(19)

Following general procedure **1**: NHC precatalyst **7** (57.0 mg, 0.10 mmol, 0.10 equiv), KHMDS (0.18 mL, 0.09 mmol, 0.09 equiv), methyl-1-napthylketene (182 mg, 1.00 mmol, 1.0 equiv) and chloral (0.10 mL, 1.00 mmol, 1.0 equiv) in toluene (20 mL) at 0 °C for 12 h gave crude product that was purified by flash silica chromatography (1% ether:petrol) to give **19** as a colorless oil; (207 mg, 63% yield); $[a]_D^{20}$ + 108 (c 0.5, $CHCl_3$); Chiral HPLC analysis; Chiralcel OJ-H (1% IPA:hexane, flow rate 1 mL min^{-1}, 220 nm) t_R minor (*R*): 7.5 min, t_R major (*S*): 15.5 min, 84% *ee*; ν_{max} (thin film)/cm^{-1} 3094, 1768 (C=O), 1646, 1513, 1452, 1377, 1214, 1124, 971, 883, 851, 778 and 738; ^1H NMR (400 MHz, $CDCl_3$) δ_H 2.43 (3H, s), 7.47 (1H, s), 7.49-7.53 (3H, m), and 7.85-7.96 (4H, m); ^{13}C NMR (100 MHz $CDCl_3$): δ_C 29.9, 68.8, 115.0, 123.8, 124.3, 124.9, 126.0, 126.9, 129.5, 130.1, 130.6, 133.2, 134.3, 134.6 and 168.3; *m/z*: (EI+) $(M(^{35}Cl))^+$ HRMS $C_{15}H_{11}O_2Cl_3$ [M+]: found 327.9817; calcd 327.9819.

(20)-(*S*)-2,2-Dichlorovinyl 2-chloro-2-(2-chlorophenyl)butanoate

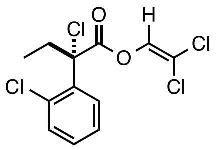

(20)

Following general procedure **1**: NHC precatalyst **7** (57.0 mg, 0.10 mmol, 0.10 equiv), KHMDS (0.18 mL, 0.09 mmol, 0.09 equiv), ethyl-2-chlorophenylketene (181 mg, 1.00 mmol, 1.0 equiv) and chloral (0.10 mL, 1.00 mmol, 1.0 equiv) in toluene (20 mL) at 0 °C for 12 h gave crude product that was purified by flash silica chromatography (2% ether:petrol) to give **20** as a colorless oil; (205 mg, 65% yield); $[a]_D^{20}$ +12 (c 0.22, $CHCl_3$); Chiral HPLC analysis; Chiralcel OJ-H (1% IPA:hexane, flow rate 1 mL min^{-1}, 220 nm) t_R major (*S*): 7.0min, t_R minor (*R*): 9.7 min, 62% *ee*; ν_{max} (thin film)/cm^{-1} 3094, 2981, 2941, 1771 (C=O), 1469, 1435, 1286, 1197, 1124, 995, 910 and 752; ^{1}H NMR (300 MHz, $CDCl_3$) δ_H 0.87 (3H, t, *J* 7.3), 2.53 (1H, dq, *J* 7.3, 14.7), 2.67 (1H, dq, *J* 7.3, 14.7), 7.30-7.40 (3H, m), 7.59 (1H, s), and 7.83-7.85 (1H, m); ^{13}C NMR (100 MHz $CDCl_3$): δ_C 8.5 (CH_3), 31.7, 73.8, 114.6, 127.1, 130.1, 130.2, 130.8, 131.7, 133.4, 135.2 and 166.1; *m/z*: (CI+) (M(^{35}Cl))$^+$ HRMS $C_{12}H_{14}O_2N_1Cl_4$ [M+NH_4]$^+$: found 343.9778; calcd 343.9773.

(21)-(*S*)-2,2-Dichlorovinyl 2-chloro-2-(naphthalene-1-yl)hexanoate

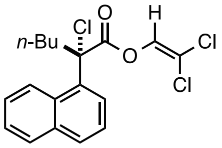

(21)

Following general procedure **1**: NHC precatalyst **7** (57.0 mg, 0.10 mmol, 0.10 equiv), KHMDS (0.18 mL, 0.09 mmol, 0.09 equiv), *n*-butyl-1-napthylketene (224 mg, 1.00 mmol, 1.0 equiv) and chloral (0.10 mL, 1.00 mmol, 1.0 equiv) in toluene (20 mL) at 0 °C for 12 h gave crude product that was purified by flash silica chromatography (1% ether:petrol) to give **21** as a colorless oil; (311 mg, 84% yield); $[a]_D^{20}$ + 88.5 (c 1.0, $CHCl_3$); Chiral HPLC analysis; analysis conducted at AstraZeneca Chiralcel OJ-H (10% ethanol:*i*-hexane, flow rate 1 mL min^{-1}, 220 nm) t_R minor (*R*): 1.02 min, t_R major (*S*): 1.22 min, 90% *ee*; ν_{max} (thin film)/cm^{-1} 3094, 2960, 2873, 1761 (C=O), 1601, 1513, 1468, 1282, 1199, 1128, 984, 908, 859, 775, 734, 653 and 619; ^{1}H NMR (400 MHz, $CDCl_3$) δ_H 0.92 (3H, t, *J* 7.0), 1.37-1.43 (4H, br m), 2.70-2.77 (2H, br m), 7.47 (1H, s), overlapping 7.51-7.54 (3H, m), 7.88-7.92 (3H, m) and 7.96-7.99 (1H, m); ^{13}C NMR (75 MHz $CDCl_3$): δ_C 13.7, 22.4, 26.3, 39.9, 73.2, 114.7, 123.3, 124.6, 125.0, 125.6, 126.6, 129.3, 129.9, 130.1, 132.9, 133.6, 134.1 and 167.4; *m/z*: (EI+) (M(^{35}Cl))$^+$ HRMS $C_{15}H_{11}O_2Cl_3$ [M+]: found 370.0292; calcd 370.0289.

(28)-3-Ethyl-3-(2-methoxyphenyl)-4-(trichloromethyl)oxetan-2-one and (S9)-2,2-Dichlorovinyl 2-chloro-2-(2-mhoxyphenyl)butanoate.

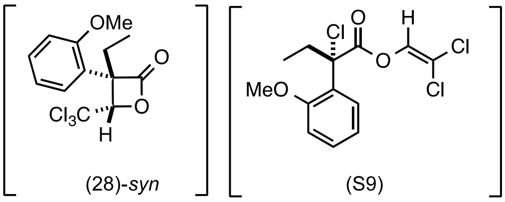

(~67:33 28:S9)

Following general procedure **1**: NHC precatalyst **7** (57.0 mg, 0.10 mmol, 0.10 equiv), KHMDS (0.18 mL, 0.09 mmol, 0.09 equiv), ethyl-2-methoxyphenylketene (176 mg, 1.00 mmol, 1.0 equiv) and chloral (0.10 mL, 1.00 mmol, 1.0 equiv) in toluene (20 mL) at 0 °C for 3 h gave crude product as a ~67:33 mixture of lactone **28** (*dr* >95:5, *syn:anti*) and **S9** that was purified by flash silica chromatography (1% ether:petrol) **S9** as a colorless oil; (37 mg, 11% yield); $[a]_D^{20}$ +5 (c 0.75, $CHCl_3$); ν_{max} (thin film)/cm^{-1} 3091, 2977, 2940, 1772 (C=O), 1601, 1587, 1489, 1464, 1437, 1247, 1195, 1135, 1089, 1026, 992, 906, 863, 752 and 616; ^{1}H NMR (400 MHz, $CDCl_3$) δ_H 0.87 (3H, t, *J* 7.3), 2.37-2.51 (2H, m), 3.77 (3H, s), 6.87 (1H, dd, *J* 7.5, 8.2), 7.02 (1H, td, *J* 1.1, 7.6), 7.34 (1H, ddd, *J* 1.6, 7.5, 8.2), 7.55 (1H, s,) and 7.65 (1H, dd, *J* 1.6, 7.5); ^{13}C NMR (100 MHz $CDCl_3$): δ_C 8.4, 31.9, 55.5, 72.3, 110.1, 113.5, 120.7, 126.7, 128.4, 130.0, 133.5, 155.2 and 167.0; *m/z*: (ESI +) (M(^{35}Cl))$^+$ HRMS $C_{13}H_{13}O_3Cl_3$ [M+]$^+$: found 321.9928; calcd 321.9925.

28 as a colorless solid; (158 mg, 48% yield); mp 76-79 °C; $[a]_D^{20}$+140 (c 0.66, $CHCl_3$); Chiral HPLC analysis; Chiralpak AD-H (1% IPA:hexane, flow rate 1 mL min^{-1}, 220 nm) t_R major (3*S*,4*S*): 6.9 min, t_R minor (3*R*,4*R*) 7.8 min >99% ee; ν_{max} (KBr film)/cm^{-1} 2983, 1832, 1601 (C=O), 1585, 1499, 1464, 1438, 1292, 1256, 1182, 1148, 1088, 1026, 905, 838 and 809; ^{1}H NMR (400 MHz, $CDCl_3$) δ_H 0.87 (3H, t, *J* 7.4), 2.27 (1H, dq, *J* 7.0, 13.9), 3.01 (1H, dq, *J* 7.0, 13.9), 3.76 (3H, s), 4.92 (1H, s) 6.85 (1H, d, *J* 8.3), 6.9 (1H, *app* t, *J* 7.5), 7.30 (1H, m) and 7.58 (1H, d, *J* 7.4); ^{13}C NMR (100 MHz $CDCl_3$): δ_C 9.1, 29.8, 54.5, 68.4, 87.5, 96.4, 110.9, 118.9, 120.4, 130.1, 131.4, 156.6 and 170.4; *m/z*: (ESI+) (M(^{35}Cl))$^+$ HRMS $C_{13}H_{13}O_3Cl_3$ [M+]$^+$: found 321.9926; calcd 321.9925.

Additional Computational Information

DFT calculations were made using Firefly software.[14] Grimme's B3LYP-D3(BJ) functional[15] and the 6-31G(d, p) basis set[16] were used for transition state geometry optimisation and ZPE calculation, with final single-point energies recalculated using the TZVPP basis set.[17]

Energies (in Hartrees or kJ/mol) of [2+2] and chlorination transition states. The final relative energy is based on the combination of the TZVPP sinlge point energy and the 6-31G(d,p) zero-point energy (ZPE).

	B3LYP-D3(BJ) 6-31G(d,p) (H)	ZPE (H)	B3LYP-D3(BJ) TZVPP (H)	TZVPP + ZPE (H)	relative (kJ/mol)
2-MePh [2+2] **22**	-2315.847294	0.322758	-2316.286528	-2315.96377	0
2-MePh chlorination E-enolate **24**	-2315.852707	0.320689	-2316.295755	-2315.975066	-29.7
2-MePh chlorination Z-enolate **24b** (not in main text)	-2315.839135	0.320339	-2316.283519	-2315.96318	1.5
iPr [2+2] **23**	-2355.171694	0.351198	-2355.623059	-2355.271861	0
iPr chlorination E-enolate **25**	-2355.17133	0.348969	-2355.62659	-2355.277621	-15.1
iPr chlorination Z-enolate **25b** (not in main text)	-2355.153544	0.349635	-2355.611382	-2355.261747	26.5

2-Methylphenyl, methylketene

[2+2] Transition state **22**

C	6.0	1.0507385129	0.8748725377	-1.0889337887
C	6.0	-0.0245756814	-0.1375925815	-1.0363143696
O	8.0	1.1193160357	1.7883620191	-1.9075396158
C	6.0	-0.4881311897	-0.3124346972	-2.4960187383
C	6.0	2.1820530153	0.9563723299	-0.0830404822
C	6.0	0.3032225445	-1.4401564531	-0.3110847555
C	6.0	0.2503711650	-2.6254651454	-1.0775492141
C	6.0	0.5945883627	-3.8749010203	-0.5758756113
C	6.0	1.0155255513	-3.9918663348	0.7455748552
C	6.0	1.0595384817	-2.8445902462	1.5274397001
C	6.0	0.7071993632	-1.5727654276	1.0456354184
N	7.0	2.4614103686	2.0572274445	0.6496706064
N	7.0	3.2216420131	0.1339725066	0.0801658783
C	6.0	3.6592445605	1.8204030957	1.2656415668
C	6.0	1.6504596997	3.2828804456	0.7199947068
N	7.0	4.1515898424	0.6539464847	0.9356060173

C	6.0	3.4701914883	-1.1701632710	-0.5285962333
C	6.0	-2.6230833712	0.2485673850	0.1843785686
C	6.0	-1.2774781997	0.9951827079	-0.2186092381
CL	17.0	-2.4471415774	-0.6932488640	1.6981842887
CL	17.0	-3.3293413477	-0.8402718615	-1.0782194549
CL	17.0	-3.8324214154	1.5754127331	0.4736960514
O	8.0	-0.7948775756	1.6582946281	0.7621048964
C	6.0	0.8084241768	-0.4581350972	2.0662802333
H	1.0	-0.8206428263	0.6456598416	-2.8936448029
H	1.0	-1.3078954939	-1.0177565587	-2.5788868603
H	1.0	0.3354097679	-0.6516200504	-3.1350825073
H	1.0	-0.0643485495	-2.5745081012	-2.1096602116
H	1.0	0.5311989259	-4.7474694763	-1.2188220010
H	1.0	1.2884107791	-4.9556944615	1.1641900634
H	1.0	1.3628745499	-2.9215576915	2.5686795685
H	1.0	4.1297774742	2.5202359808	1.9382371733
H	1.0	1.9299438259	3.8140690037	1.6296586880
H	1.0	0.5952195581	2.9759705352	0.7414660774
H	1.0	1.8598628857	3.8894144772	-0.1617802338
H	1.0	4.5008549755	-1.1780871257	-0.8817776841
H	1.0	2.7764275904	-1.3107888697	-1.3530775180
H	1.0	3.3045702994	-1.9533515124	0.2109952106
H	1.0	-1.5089849815	1.5318925173	-1.1609826754
H	1.0	0.2679010392	-0.7506747752	2.9708834790
H	1.0	0.3546425563	0.4755591361	1.7315961455
H	1.0	1.8530808003	-0.3044341872	2.3680808028

2-Methylphenyl, methylketene

Chlorination transition state (E enolate) **24**

C	6.0	1.3970340955	-0.1826462564	-1.3879771042
C	6.0	0.7267917768	1.8023675920	0.0779549541
C	6.0	1.3195751283	1.2666534805	-1.2159343927
O	8.0	1.4449698748	2.1051419361	-2.1129944541
C	6.0	2.1696553755	-0.9859639492	-0.3686380011
C	6.0	1.5617294477	-0.6241700875	-2.8252026494
C	6.0	3.5769351759	-0.8226018441	-0.2959211838
C	6.0	4.2944481915	-1.5614662454	0.6532741861
C	6.0	3.6660419049	-2.4614949380	1.5102951763
C	6.0	2.2883298164	-2.6429356100	1.4164788880
C	6.0	1.5537564864	-1.9122191344	0.4839479123
N	7.0	1.0164310634	1.6693621720	1.3763371355
C	6.0	-0.7893078578	2.7657389125	1.3277668386
N	7.0	-0.4095424844	2.5419071216	0.0357254108
N	7.0	0.0725989963	2.2657134877	2.1719519779
C	6.0	2.1613364809	1.0487358540	2.0366614083
C	6.0	-1.1377768708	2.9720868280	-1.1628403375
H	1.0	0.8831322636	-0.0708274611	-3.4760134514
H	1.0	1.3492100789	-1.6921809808	-2.9044302710
H	1.0	2.5817228278	-0.4488560247	-3.1845990355
H	1.0	5.3703637544	-1.4218459297	0.7163334869
H	1.0	4.2486941681	-3.0167110473	2.2389466412
H	1.0	1.7780818375	-3.3453329209	2.0679588311
H	1.0	0.4822242539	-2.0443279570	0.4283583005
H	1.0	-1.7050813779	3.2660845916	1.5944709555
H	1.0	2.3575237352	1.6323419989	2.9347328841
H	1.0	3.0188254455	1.0703669433	1.3705211227
H	1.0	1.9302964573	0.0156499417	2.2946885128
H	1.0	-0.6053213854	3.7987999097	-1.6314477972
H	1.0	-2.1436567875	3.2396570089	-0.8478733533
H	1.0	-1.1981218986	2.1330272669	-1.8523758242

CL	17.0	-0.7756616006	-0.4717887423	-0.9543745321
C	6.0	-2.9113539990	-0.7976385231	0.0658131382
C	6.0	-3.4384281845	0.4930112203	-0.3070513411
CL	17.0	-2.4273881066	-1.0097340052	1.7404417610
CL	17.0	-3.6692072222	-2.2079496119	-0.6672537423
O	8.0	-3.2697190741	1.5435878869	0.3171834565
H	1.0	-3.9487157350	0.4783466830	-1.2899503958
C	6.0	4.3504488908	0.1065278988	-1.2058160535
H	1.0	3.7967638224	1.0064268636	-1.4793514660
H	1.0	4.6162325023	-0.3971533160	-2.1421883096
H	1.0	5.2865426953	0.4138486040	-0.7317439559

2-Methylphenyl, methylketene

Chlorination transition state (Z enolate) **24b** (not in main text)

Cl	17.0	-1.9299337847	-7.5271862387	2.1587191030
C	6.0	-1.3071644795	-5.5465939179	2.9468569194
C	6.0	-3.8158791408	-5.2445497310	2.3750887488
C	6.0	-2.3563849040	-4.8467380607	2.2079802306
O	8.0	-2.1768195579	-4.0456824078	1.2863366716
C	6.0	0.0979609526	-5.1560728710	2.5818496240
C	6.0	-1.4846200120	-5.9385289783	4.4010227076
C	6.0	0.9884033423	-6.1151483106	2.0860445087
C	6.0	2.3165046672	-5.7954449334	1.8137193059
C	6.0	2.7747967556	-4.5010379676	2.0419954495
C	6.0	1.8978257805	-3.5428712521	2.5443673381
C	6.0	0.5592780906	-3.8412451933	2.8231200641
N	7.0	-4.6856982926	-5.1343273537	3.3818836026
C	6.0	-5.8347468245	-5.8001045840	1.7502699369
N	7.0	-4.5516304580	-5.6434588216	1.3102045479
N	7.0	-5.9517974745	-5.4795431096	3.0093578662
C	6.0	-4.4689607345	-4.7739788543	4.7757184075
C	6.0	-4.0538843642	-5.8818217479	-0.0474995805
C	6.0	-0.3326223650	-2.7436406638	3.3522600336
H	1.0	-1.3826700520	-5.0651521857	5.0578314061
H	1.0	-2.4408802090	-6.4311833243	4.5815359713
H	1.0	-0.6920099007	-6.6392897440	4.6690871990
H	1.0	0.6221830810	-7.1183113724	1.9057095479
H	1.0	2.9843868362	-6.5569516878	1.4237474687
H	1.0	3.8073029543	-4.2367259674	1.8347781693
H	1.0	2.2544632656	-2.5331434268	2.7287721052
H	1.0	-6.6399710780	-6.1445484762	1.1222752190
H	1.0	-4.4349592949	-5.6916896352	5.3642439037
H	1.0	-3.5377262380	-4.2194082558	4.8646540928
H	1.0	-5.3086372648	-4.1541179314	5.0876624132
H	1.0	-4.8805214483	-6.2666000855	-0.6421633824
H	1.0	-3.6758565404	-4.9444244213	-0.4526502557
H	1.0	-3.2578903239	-6.6236577489	-0.0006060434
H	1.0	-0.9922392691	-2.3689704284	2.5642122446
H	1.0	-0.9713968407	-3.0838183513	4.1741751107
H	1.0	0.2659122153	-1.9093556742	3.7261638836
C	6.0	-3.6411842941	-9.4943990817	2.2473764765
C	6.0	-3.8562558900	-8.9964243073	3.5719185726
CL	17.0	-4.7824718523	-9.0534692514	0.9862323542
CL	17.0	-2.9162210926	-11.0779703498	2.0539268789
O	8.0	-4.5183465045	-7.9956750973	3.8726831825
H	1.0	-3.2599444557	-9.5430451983	4.3283219959

Phenyl, isopropylketene

[2+2] Transition state **23**

C	6.0	-1.0544110611	0.8511299928	0.9052715182

C	6.0	-0.0172440752	-0.1991713102	0.8853536414
O	8.0	-1.0742673738	1.8333992362	1.6435928068
C	6.0	0.4813695070	-0.4819912809	2.3383254451
C	6.0	-2.2147706500	0.8842823679	-0.0823577771
C	6.0	-0.3669699525	-1.4233368892	0.0728489077
C	6.0	-0.2910332171	-2.7274864242	0.5910978398
C	6.0	-0.6667889111	-3.8337407727	-0.1698788431
C	6.0	-1.1238895946	-3.6735907292	-1.4774711326
C	6.0	-1.1644330837	-2.3904623527	-2.0246376931
C	6.0	-0.7856713434	-1.2857979530	-1.2657782489
N	7.0	-2.5027279756	1.9459098593	-0.8700151859
N	7.0	-3.2789504530	0.0780048221	-0.1598126213
C	6.0	-3.7270986932	1.6981003886	-1.4282888487
C	6.0	-1.6788741655	3.1503785154	-1.0453598128
N	7.0	-4.2276053871	0.5631228792	-1.0133006893
C	6.0	-3.4955616020	-1.2130387722	0.4848117945
C	6.0	2.4930209702	0.0909420203	-0.5638020959
C	6.0	1.1438897399	0.8381189733	-0.1559126349
CL	17.0	2.3113455881	-0.8263780819	-2.0891179440
CL	17.0	3.2009806875	-1.0229918516	0.6741982673
CL	17.0	3.6987610251	1.4292709756	-0.8392986148
O	8.0	0.5368236410	1.2983494716	-1.1847989995
C	6.0	1.3116653779	0.6218895797	3.0152213816
C	6.0	-0.6933125678	-0.8558420955	3.2663449004
H	1.0	1.1339808903	-1.3521587377	2.2651063265
H	1.0	0.0631000371	-2.8969790219	1.5990845577
H	1.0	-0.6003906082	-4.8259925282	0.2663943829
H	1.0	-1.4216459823	-4.5353897259	-2.0666462949
H	1.0	-1.4733167071	-2.2467203368	-3.0559845304
H	1.0	-0.7277950908	-0.3052568472	-1.7243093295
H	1.0	-4.2101102577	2.3688113295	-2.1213718342
H	1.0	-2.0609421577	3.6897214396	-1.9118937670
H	1.0	-0.6459734487	2.8087153057	-1.1969948104
H	1.0	-1.7569750575	3.7594578210	-0.1445612790
H	1.0	-4.5572203986	-1.2846121636	0.7165618542
H	1.0	-2.9022826512	-1.2545835998	1.3948394739
H	1.0	-3.1880224233	-2.0116018214	-0.1915631285
H	1.0	1.4125333535	1.5283500086	0.6626452403
H	1.0	0.7409528857	1.5445631299	3.1223810674
H	1.0	2.2345337204	0.8387219883	2.4771608689
H	1.0	1.5978235414	0.2775600393	4.0151350544
H	1.0	-1.2995919000	-1.6695799777	2.8566175621
H	1.0	-1.3410709318	0.0072833477	3.4439216833
H	1.0	-0.3164172435	-1.1879482182	4.2392005416

Phenyl, isopropylketene
Chlorination transition state (E enolate) **25**

C	6.0	1.5724606208	0.1672092884	1.1633454726
CL	17.0	-0.6671626687	0.7292048474	0.6240431558
C	6.0	-2.6845521483	1.0852967758	-0.3756715382
C	6.0	0.5073820644	-1.9126503349	0.1477385676
C	6.0	1.2965464910	-1.2521569918	1.2754150318
O	8.0	1.4100392866	-1.9632460889	2.2819581855
C	6.0	2.3676327505	0.5877226328	-0.0441426195
C	6.0	1.9370015068	0.8965917816	2.4621989398
C	6.0	3.6917913826	0.1163786974	-0.1183408881
C	6.0	4.5258313754	0.4670786763	-1.1786836267
C	6.0	4.0509542838	1.3043069924	-2.1892951545
C	6.0	2.7479831820	1.7976632193	-2.1152183425
C	6.0	1.9145966672	1.4495458922	-1.0505727797

N	7.0	0.6915216443	-2.1123342852	-1.1614565698
C	6.0	-1.2394065910	-2.8728753147	-0.7631946024
N	7.0	-0.7156140631	-2.4356636070	0.4195028215
N	7.0	-0.3975251850	-2.7081687651	-1.7468336543
C	6.0	1.8420916212	-1.8315757193	-2.0144544257
C	6.0	-1.3818276963	-2.4679953097	1.7246632888
C	6.0	0.9253528703	0.6887162184	3.5997958160
C	6.0	2.1463082331	2.3988183147	2.2245879878
H	1.0	2.8980911034	0.4675644335	2.7926486713
H	1.0	4.0631072524	-0.5351012317	0.6682561712
H	1.0	5.5418485363	0.0856703444	-1.2160451473
H	1.0	4.6932052629	1.5767906997	-3.0210092947
H	1.0	2.3724871096	2.4590636093	-2.8901434551
H	1.0	0.9037072770	1.8314102674	-1.0063171485
H	1.0	-2.2362255337	-3.2703899685	-0.8561160738
H	1.0	1.8513913533	-2.5985111354	-2.7870978068
H	1.0	2.7509631279	-1.8704726596	-1.4213110947
H	1.0	1.7450503993	-0.8420809640	-2.4601446103
H	1.0	-0.8864953175	-3.1976001922	2.3634683158
H	1.0	-2.4284610192	-2.7056785692	1.5492238215
H	1.0	-1.3156828992	-1.4789430663	2.1741556613
H	1.0	0.7809171186	-0.3682765980	3.8220192045
H	1.0	-0.0377945585	1.1325033177	3.3336544281
H	1.0	1.2887644568	1.1832660661	4.5062311494
H	1.0	1.2254079126	2.8563187573	1.8494938658
H	1.0	2.9423011795	2.5995816319	1.5041520008
H	1.0	2.4097076915	2.8886453372	3.1667538595
C	6.0	-3.4301547302	0.0753271677	0.3688908527
CL	17.0	-2.3453826090	0.7141947355	-2.0591168143
CL	17.0	-3.1675912431	2.7550786459	-0.0816747128
O	8.0	-3.5021192101	-1.1120219809	0.0681753711
H	1.0	-3.8590116157	0.4674025847	1.3111652108

Phenyl, isopropylketene
Chlorination transition state (Z enolate) **25b** (not in main text)

C	6.0	0.9177104592	0.2540086380	1.4570724879
C	6.0	0.4220119817	-2.0173460414	0.2876225830
C	6.0	1.0082513156	-1.2145237235	1.4302639389
O	8.0	1.3257295136	-1.9377096238	2.3797474549
C	6.0	1.3560278392	0.7865443300	2.7984060638
C	6.0	1.3969845752	1.1251855590	0.2587670467
C	6.0	0.5253761597	1.5676064740	3.6072494648
C	6.0	1.0032717169	2.1074281864	4.8018210588
C	6.0	2.3163869703	1.8751753411	5.2065129574
C	6.0	3.1523712888	1.0924503792	4.4087074667
C	6.0	2.6747646137	0.5514737597	3.2179382324
N	7.0	0.8245288854	-2.2276484966	-0.9684897153
C	6.0	-0.9120760417	-3.4033039987	-0.7467010972
N	7.0	-0.6639336623	-2.8045879344	0.4567886704
N	7.0	-0.0087971352	-3.0866577526	-1.6369375401
C	6.0	2.0348412796	-1.7647191095	-1.6320519990
C	6.0	-1.3807998804	-3.0531221210	1.7127312949
C	6.0	1.5143406691	2.6079478293	0.6495828133
C	6.0	0.5734760770	1.0595911619	-1.0422524558
H	1.0	2.4221367236	0.7810083366	0.0445942650
H	1.0	-0.4959627489	1.7505164102	3.2976912293
H	1.0	0.3424273387	2.7115499810	5.4159580332
H	1.0	2.6857379220	2.2962164968	6.1365666405
H	1.0	4.1756862041	0.8982567424	4.7155822943
H	1.0	3.3292427123	-0.0626194293	2.6079574775

H	1.0	-1.7699455002	-4.0313230975	-0.9238107170
H	1.0	2.6468916966	-2.6323530327	-1.8823181925
H	1.0	2.5759716265	-1.1074534783	-0.9554091029
H	1.0	1.7648830484	-1.2243614373	-2.5385949406
H	1.0	-0.8109424715	-3.7701710876	2.3042079471
H	1.0	-2.3731896047	-3.4106536418	1.4496425452
H	1.0	-1.4737182634	-2.1139318180	2.2522436300
H	1.0	2.2361893251	2.7738916643	1.4495465350
H	1.0	0.5439176928	2.9898328122	0.9791272243
H	1.0	1.8267971226	3.1865818042	-0.2250834712
H	1.0	-0.3057607270	1.6988900079	-0.9612615922
H	1.0	0.1973271749	0.0727471268	-1.3008816529
H	1.0	1.1858020533	1.4288672125	-1.8718211784
CL	17.0	-1.2548387353	0.3130423471	1.2574423450
C	6.0	-3.5497022274	0.3855526448	0.4208303236
C	6.0	-3.7976071609	-0.9929520593	0.7406069139
CL	17.0	-3.2865120302	0.7930919322	-1.2648830889
CL	17.0	-4.4296116708	1.5989402008	1.3470612661
O	8.0	-3.5093525971	-1.9634497485	0.0312425471
H	1.0	-4.1934155290	-1.1240147465	1.7679009930

HPLC Spectrum

(2)-*Anti*-(3*S*,4*R*)-3-ethyl-3-phenyl-4-(trichloromethyl)oxetan-2-one

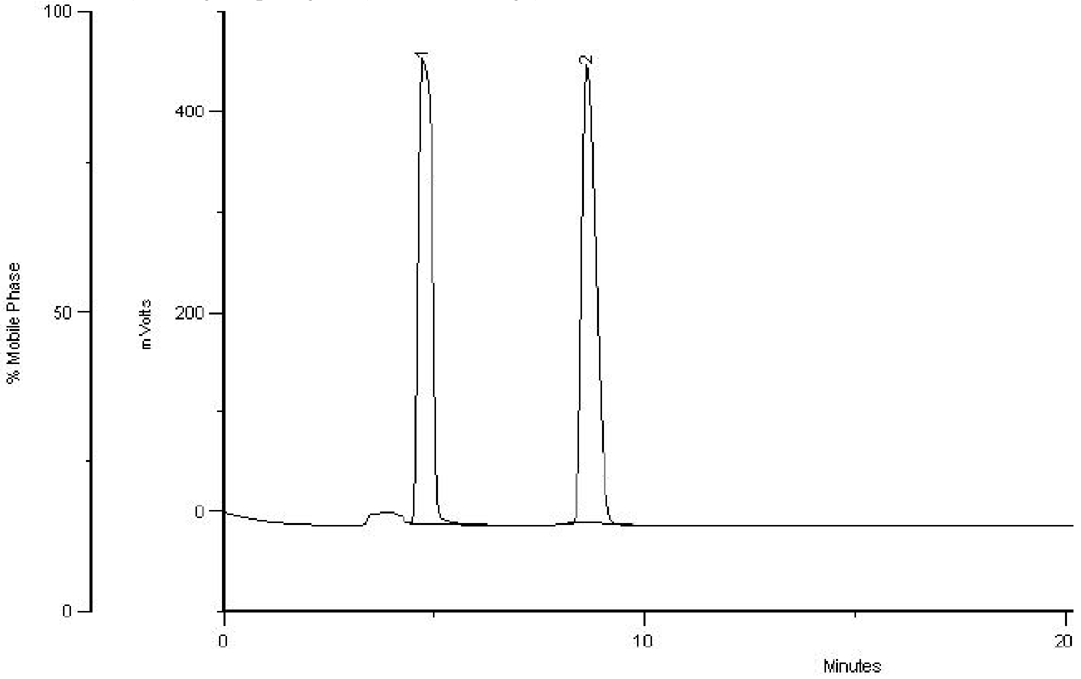

	R. Time	Area %			
1	4.74	50.19			
2	8.63	49.81			

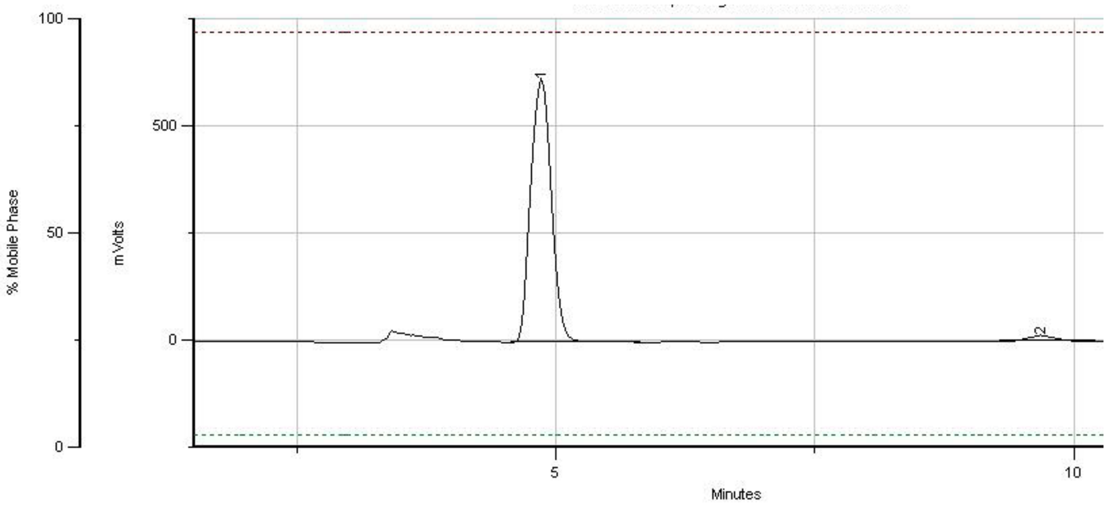

	Inj. Number	Peak Name	R. Time	Area	Area %
1	1.00	*1	4.86	14191993.00	96.98
2	1.00	*2	9.67	441868.25	3.02

(3)-*Syn*-(3*S*,4*R*)-3-ethyl-3-phenyl-4-(trichloromethyl)oxetan-2-one

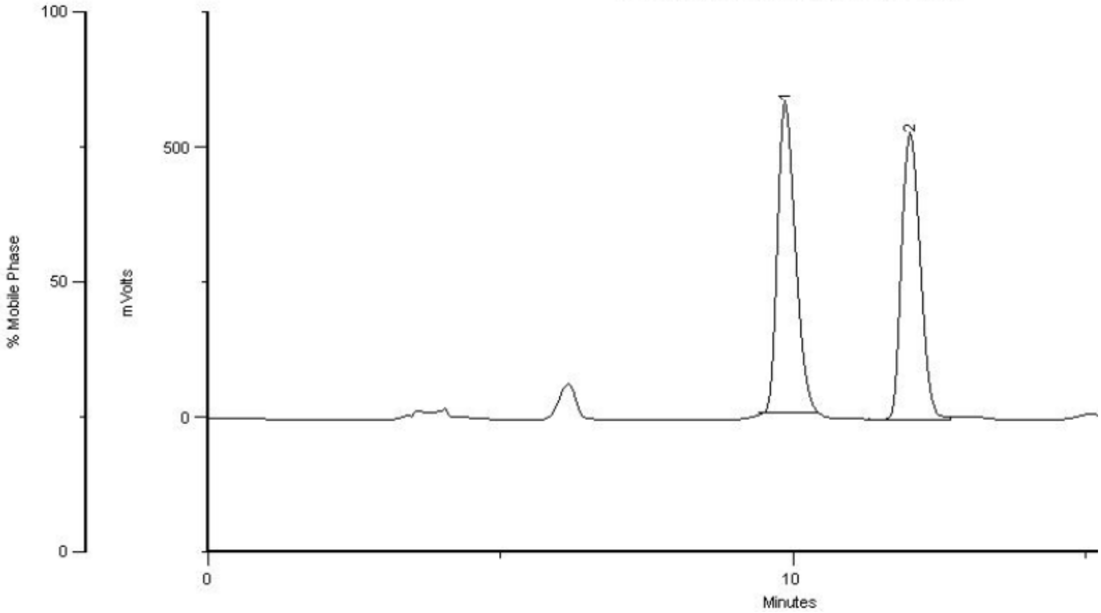

	R. Time	Area %				
1	9.87	50.60				
2	12.01	49.40				

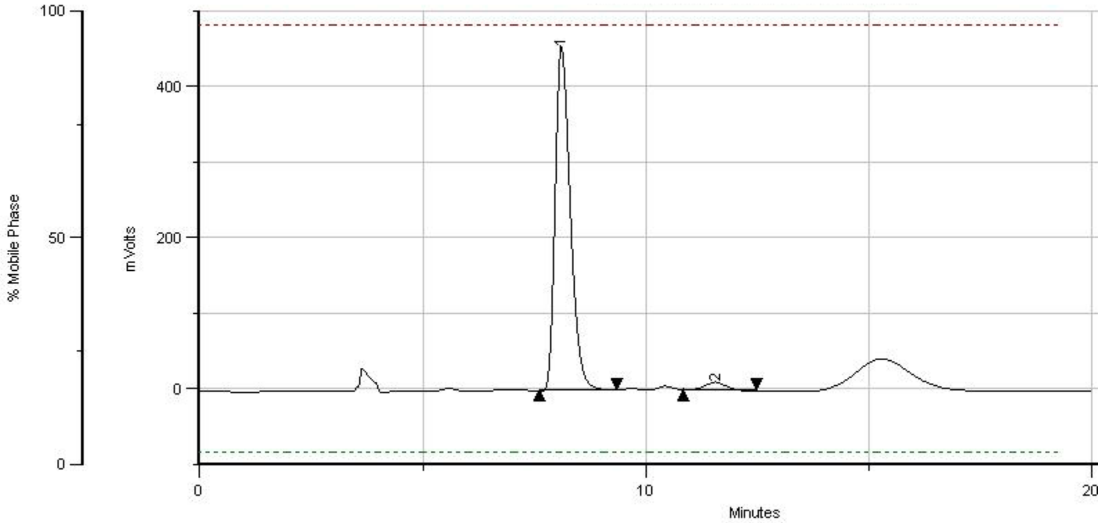

	Inj. Number	Peak Name	R. Time	Area	Area %
1	1.00	*1	8.10	18130948.00	95.88
2	1.00	*2	11.55	778876.31	4.12

(5)-(*S*)-2,2-Dichlorovinyl 2-chloro-2-(naphthalene-1-yl)butanoate **Scheme 4**

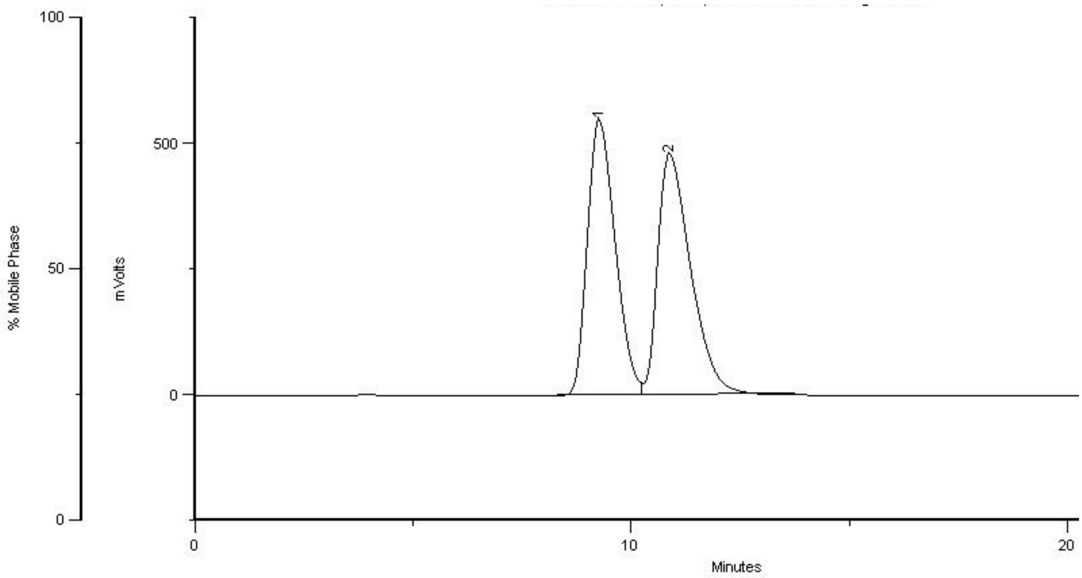

1	9.28	49.05				
2	10.90	50.95				

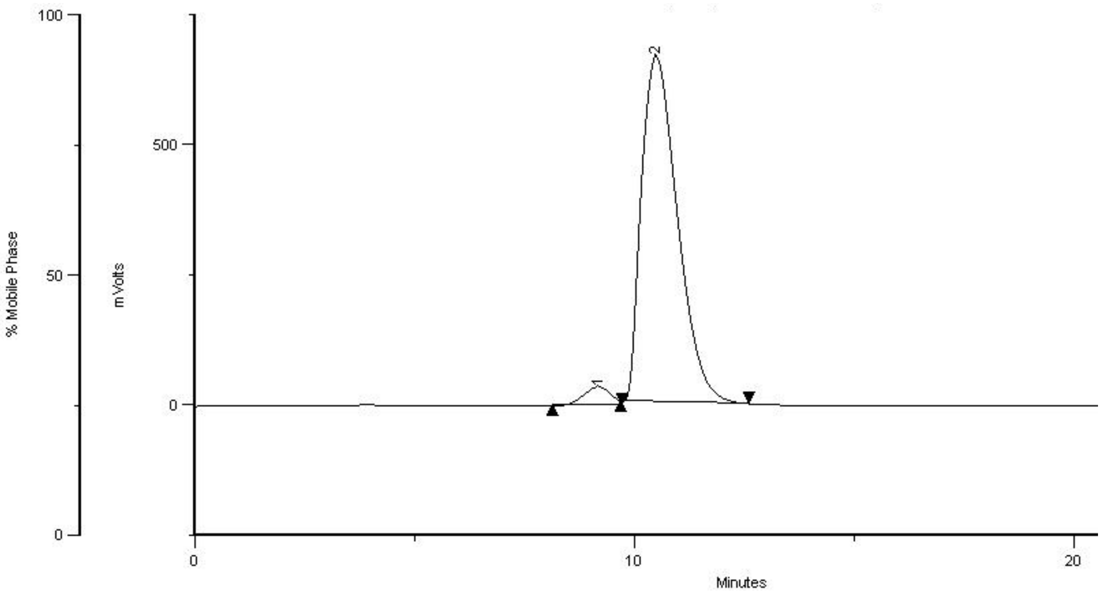

1	9.17	4.02				
2	10.49	95.98				

(5)-(*S*)-2,2-Dichlorovinyl 2-chloro-2-(naphthalene-1-yl)butanoate **Scheme 1**

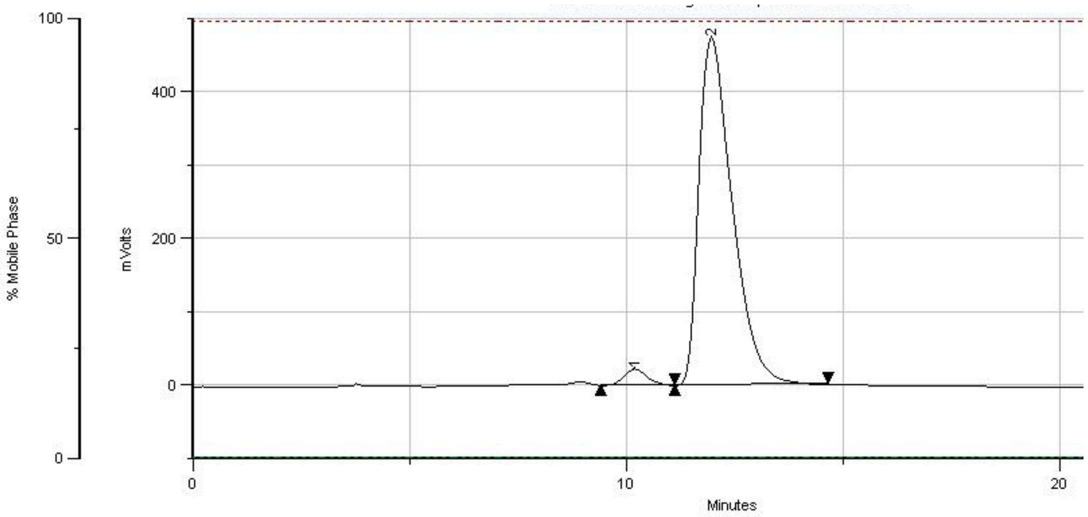

	Inj. Number	Peak Name	R. Time	Area	Area %
1	1.00	*1	10.20	1397758.75	3.18
2	1.00	2	11.96	42563616.00	96.82

(8)-*Anti*-(3*S*,4*R*)-3-(4-methoxyphenyl)-3-ethyl-4-(trichloromethyl)oxetan-2-one

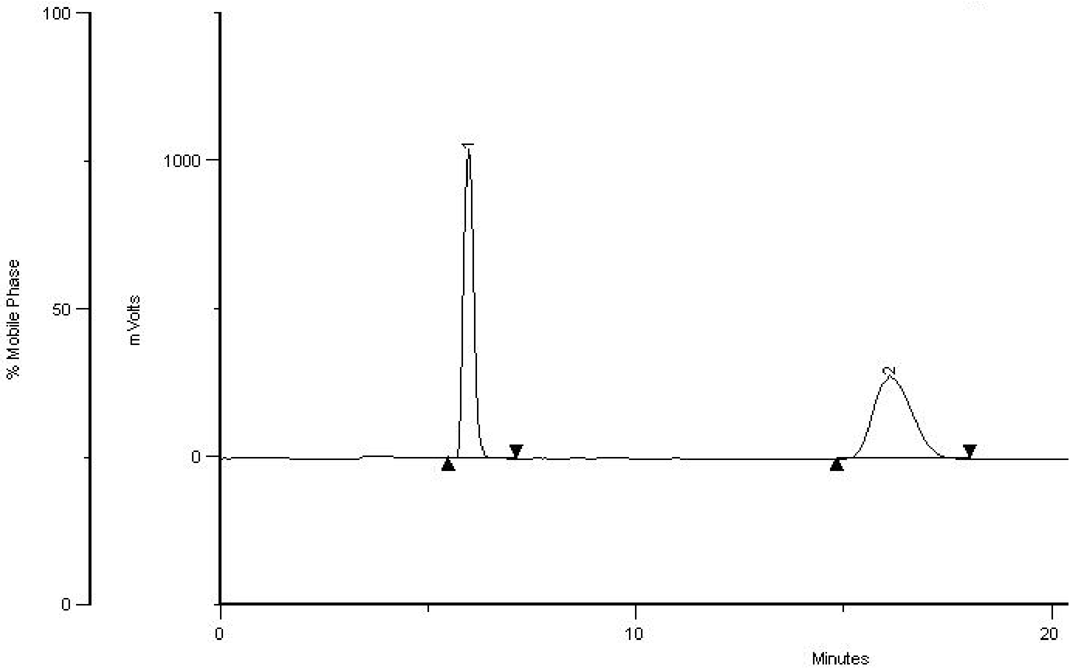

	R. Time	Area %			
1	5.97	49.68			
2	16.12	50.32			

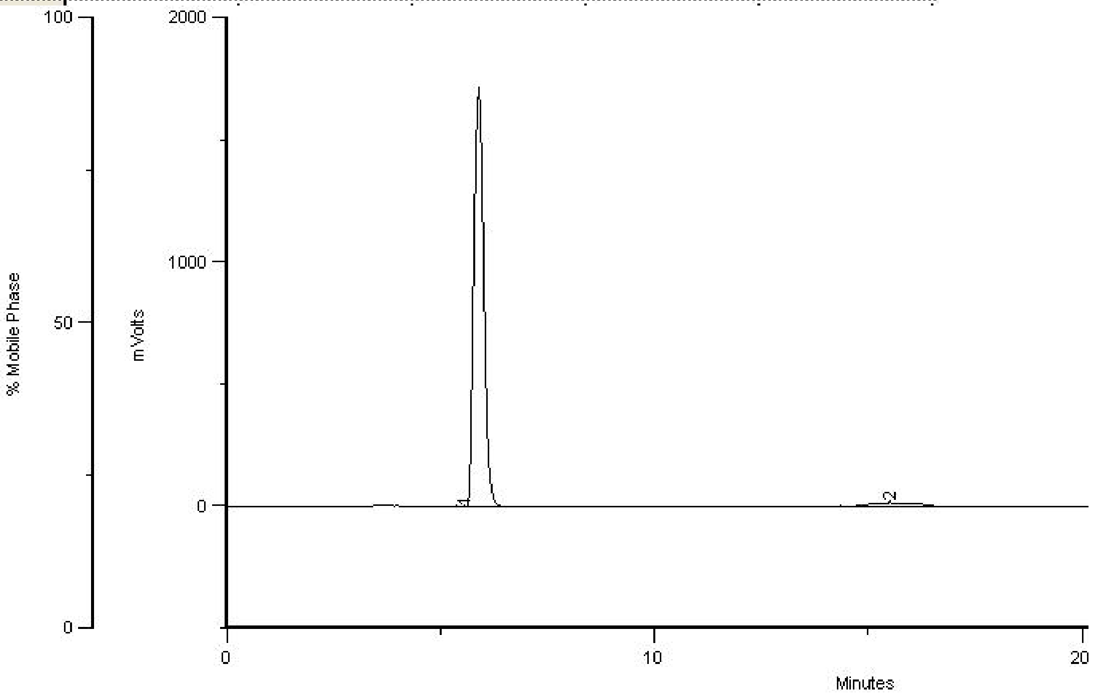

	R. Time	Area %			
1	5.89	93.84			
2	15.48	6.16			

(**8**)-*Syn*-(3*S*,4*S*)-3-(4-methoxyphenyl)-3-ethyl-4-(trichloromethyl)oxetan-2-one

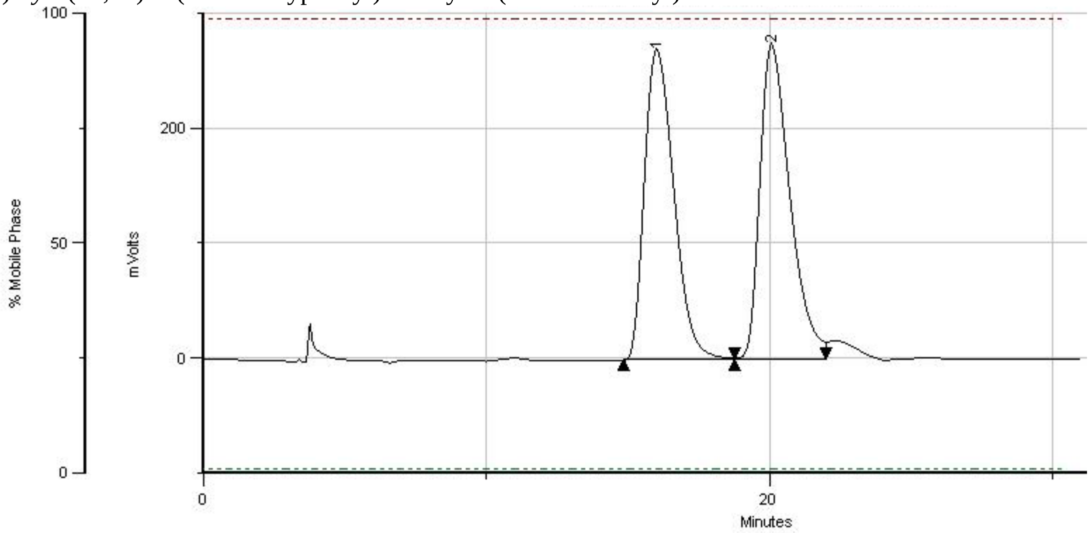

1	1.00	*1	16.02	32748912.00	48.98
2	1.00	2	20.08	34118020.00	51.02

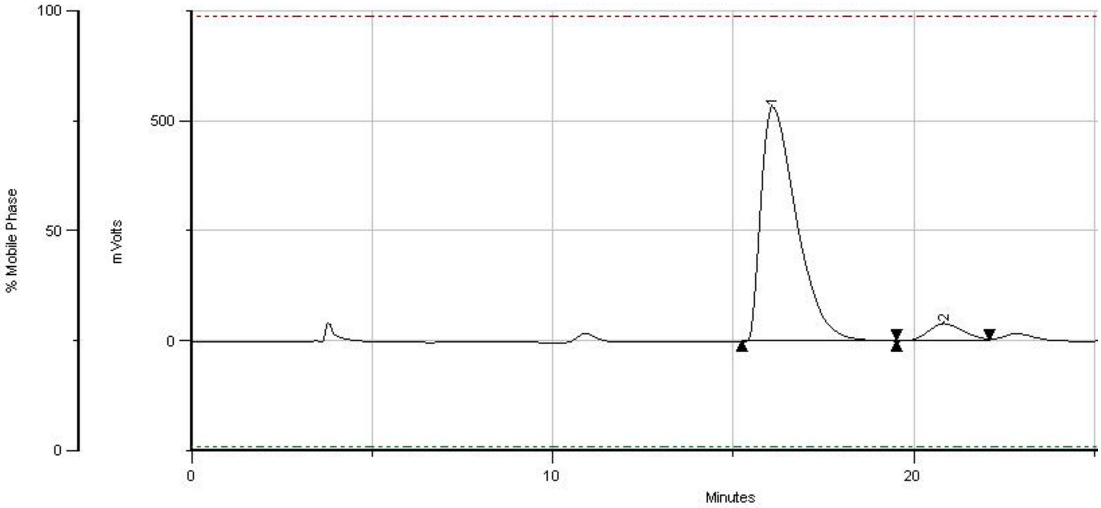

	Inj. Number	Peak Name	R. Time	Area	Area %
1	1.00	*1	16.10	61264248.00	92.43
2	1.00	2	20.84	5014589.50	7.57

(9)-*Anti*-(3*S*,4*R*)-3-(4-fluorophenyl)-3-ethyl-4-(trichloromethyl)oxetan-2-one

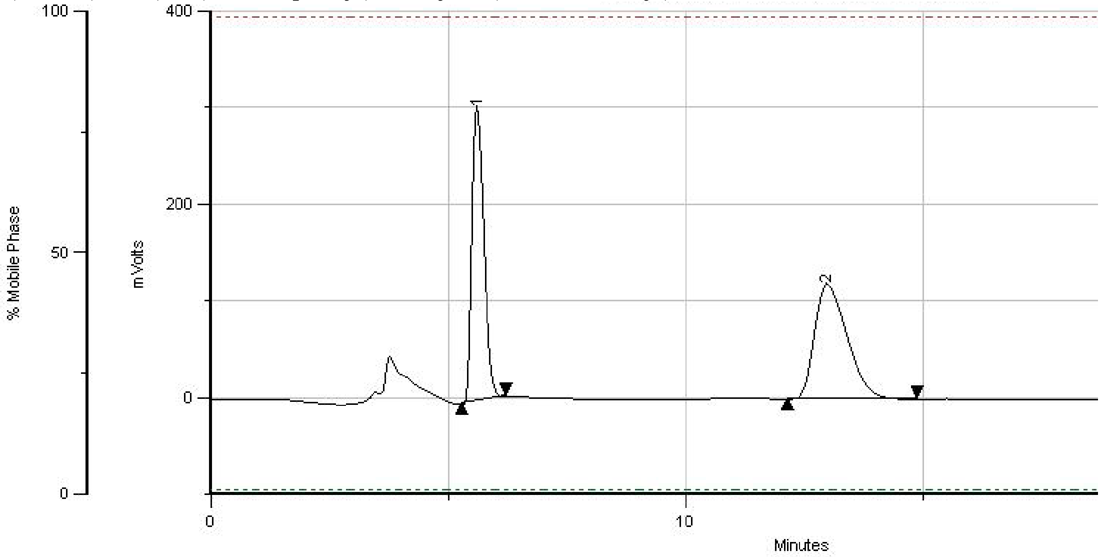

	Inj. Number	Peak Name	R. Time	Area	Area %
1	1.00	*1	5.59	8994652.00	48.80
2	1.00	*2	12.98	9438296.00	51.20

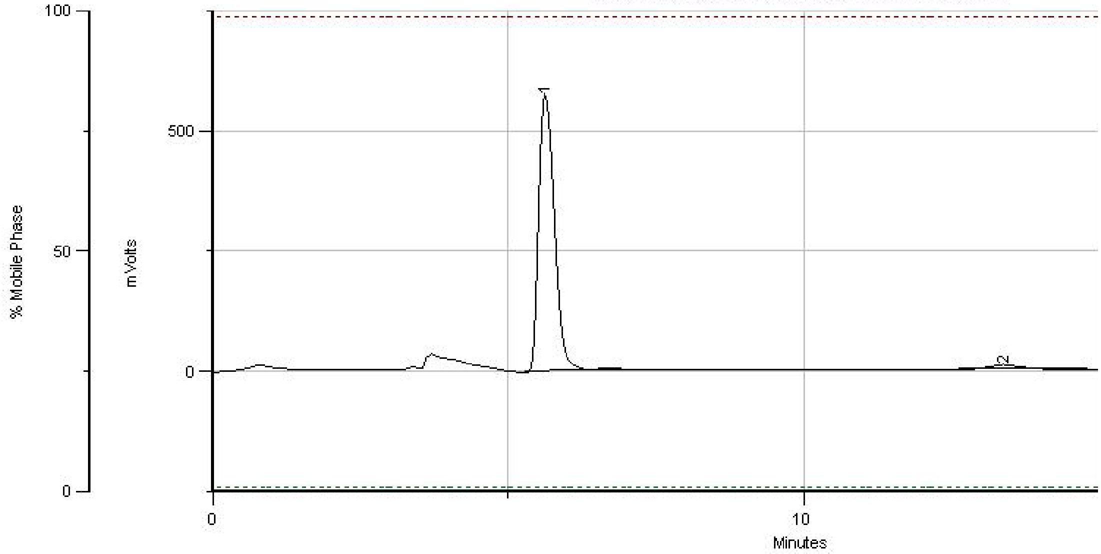

	Inj. Number	Peak Name	R. Time	Area	Area %
1	1.00	*1	5.63	18104034.00	96.50
2	1.00	*2	13.39	656941.81	3.50

(9)-*Syn*-(3*S*,4*S*)-3-(4-fluorophenyl)-3-ethyl-4-(trichloromethyl)oxetan-2-one

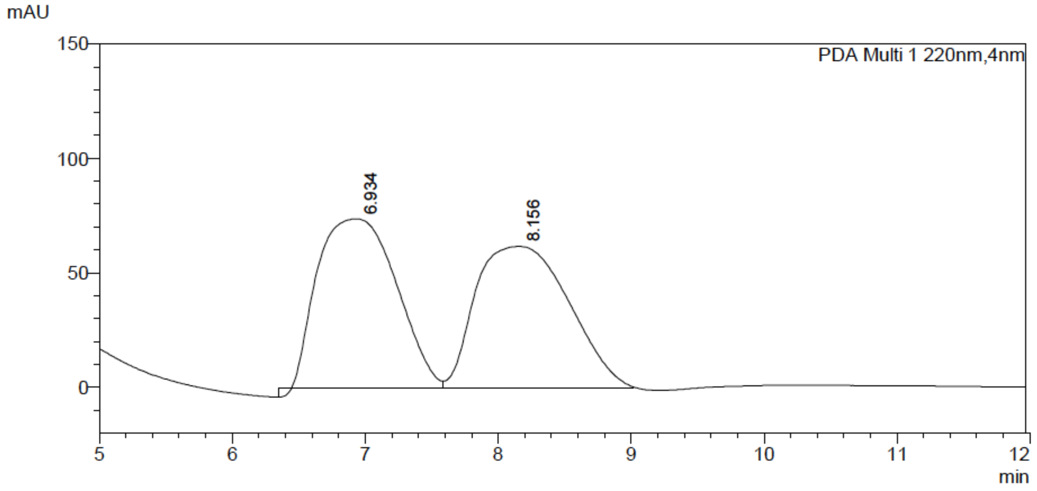

<Peak Table>

PDA Ch1 220nm

Peak#	Ret. Time	Area%
1	6.934	50.135
2	8.156	49.865
Total		100.000

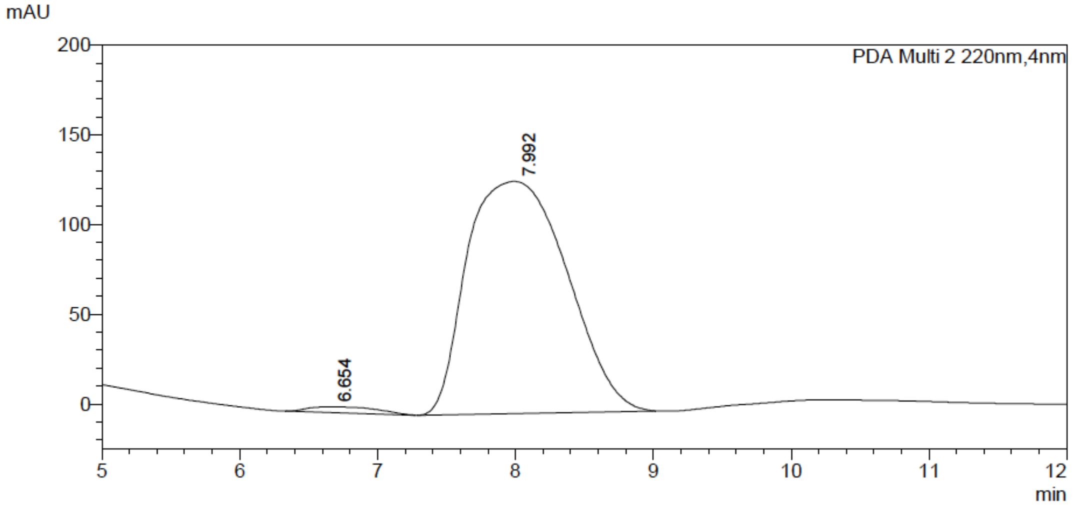

<Peak Table>

PDA Ch2 220nm

Peak#	Ret. Time	Area%
1	6.654	1.741
2	7.992	98.259
Total		100.000

(10)-*Anti*-(3*S*,4*R*)-3-(4-chlorophenyl)-3-ethyl-4-(trichloromethyl)oxetan-2-one

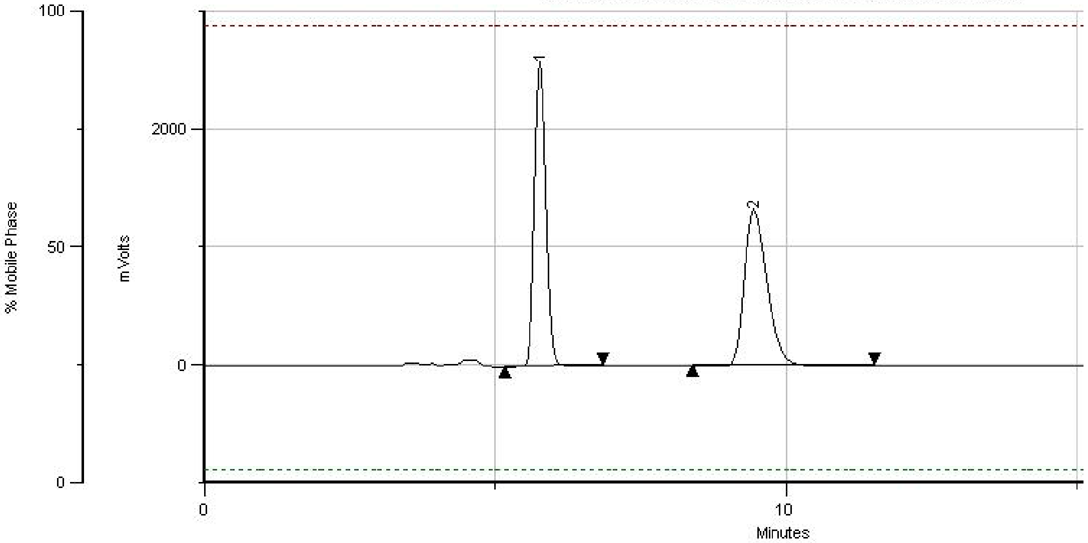

1	1.00	*1	5.76	59459520.00	49.58
2	1.00	*2	9.45	60478376.00	50.42

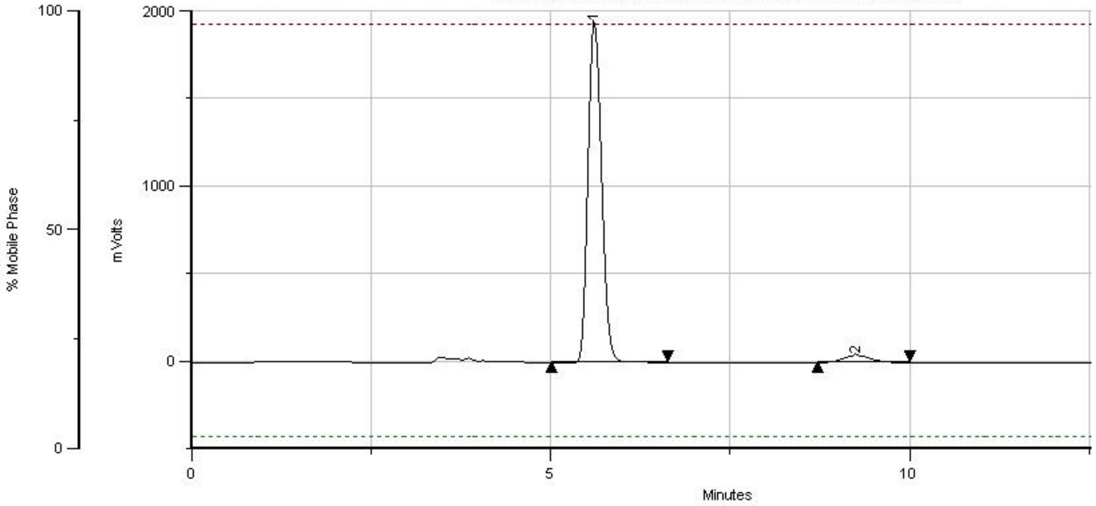

1	1.00	*1	5.61	42994060.00	95.94
2	1.00	2	9.25	1817244.38	4.06

(10)-*Syn*-(3*S*,4*S*)-3-(4-chlorophenyl)-3-ethyl-4-(trichloromethyl)oxetan-2-one

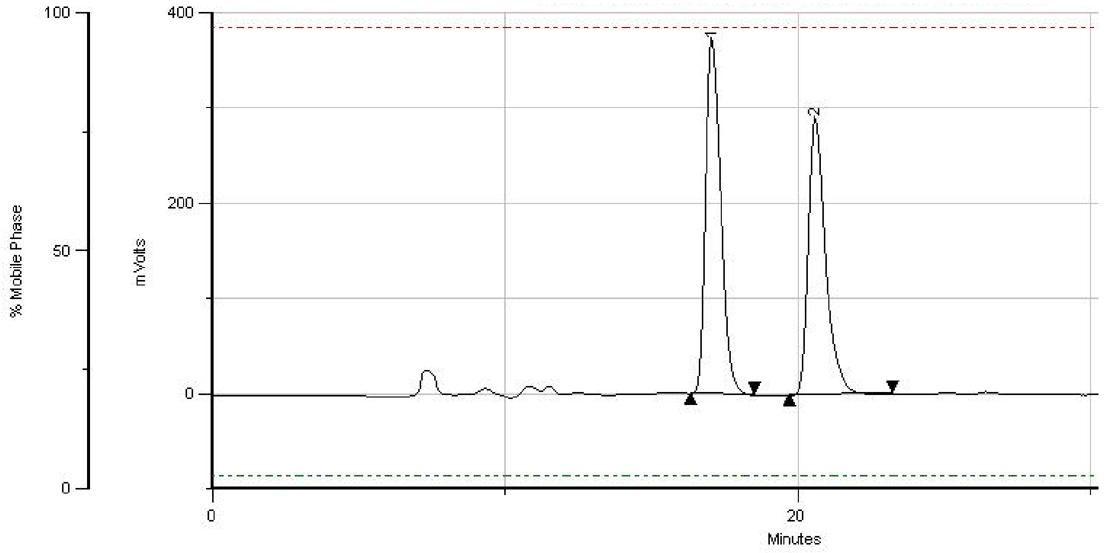

1	1.00	*1	17.07	22536728.00	52.89
2	1.00	2	20.61	20072844.00	47.11

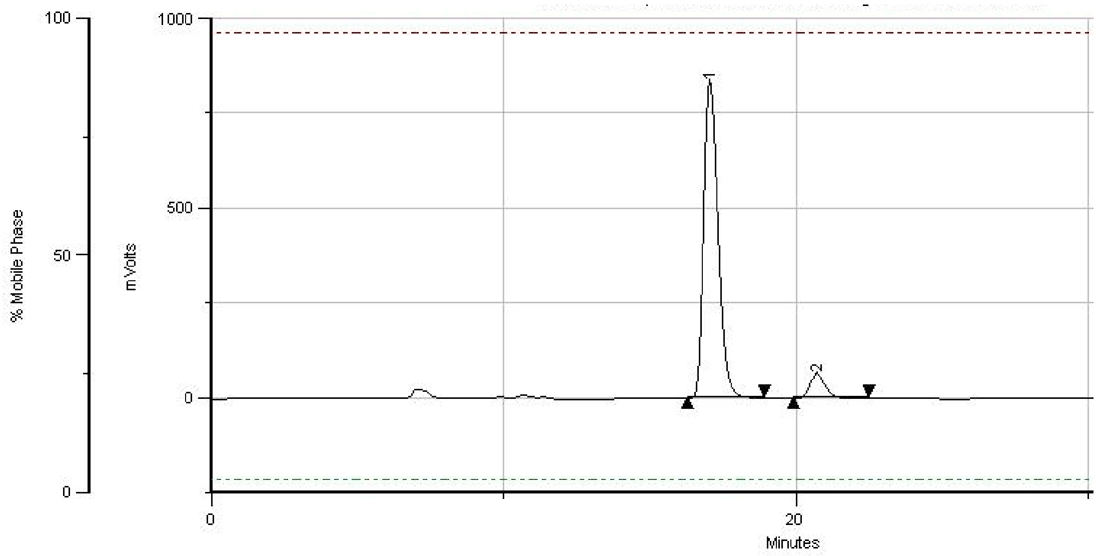

1	1.00	*1	17.06	47954592.00	92.47
2	1.00	2	20.73	3906992.25	7.53

(11)-*Anti*-(3*S*,4*R*)-3-(4-bromophenyl)-3-ethyl-4-(trichloromethyl)oxetan-2-one

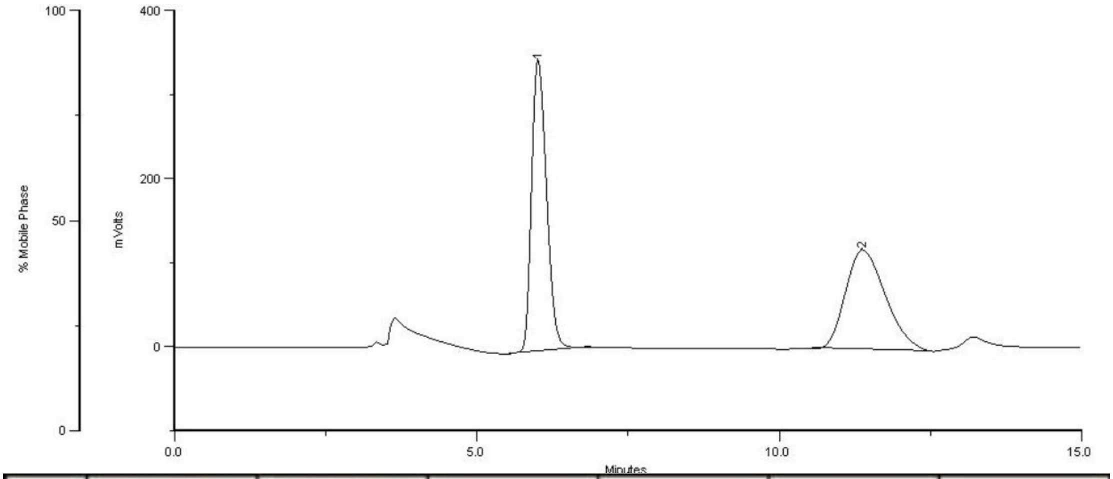

	R. Time	Area %				
1	6.01	52.36				
2	11.38	47.64				

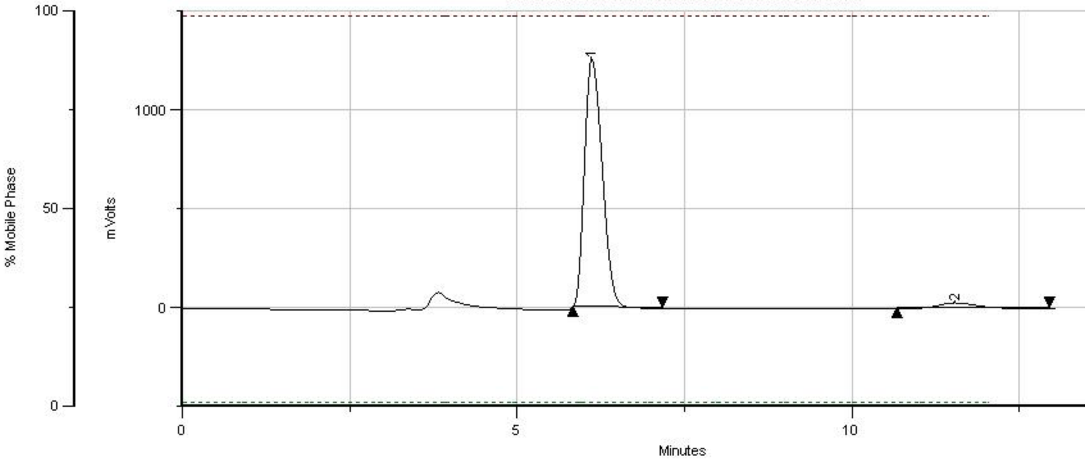

1	1.00	*1	6.12	38844756.00	94.93
2	1.00	*2	11.55	2074115.00	5.07

(11)-*Syn*-(3*S*,4*S*)-3-(4-bromophenyl)-3-ethyl-4-(trichloromethyl)oxetan-2-one

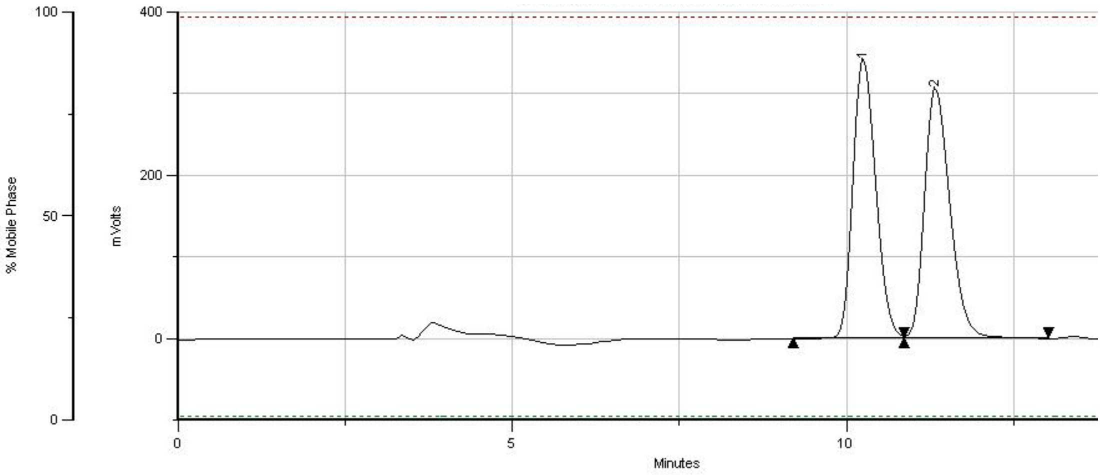

1	1.00	*1	10.25	13724133.00	49.89
2	1.00	2	11.33	13784606.00	50.11

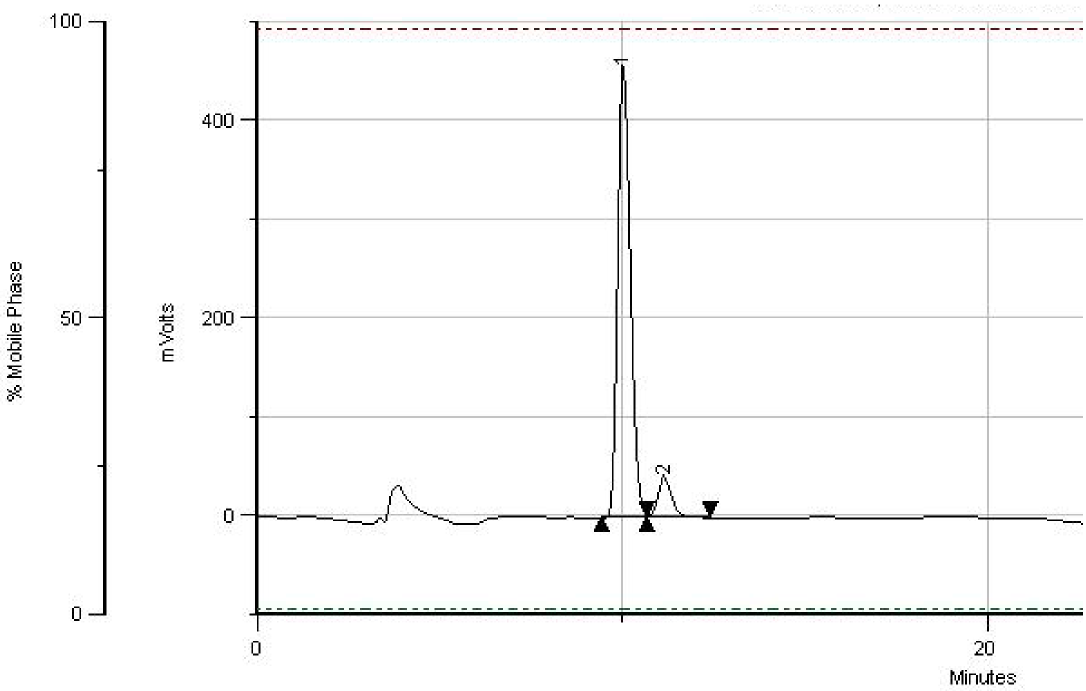

	Inj. Number	Peak Name	R. Time	Area	Area %
1	1.00	*1	10.03	18398932.00	91.10
2	1.00	2	11.16	1797697.50	8.90

(12)-*Anti*-(3*S*,4*R*)-3-methyl-3-phenyl-4-(trichloromethyl)oxetan-2-one

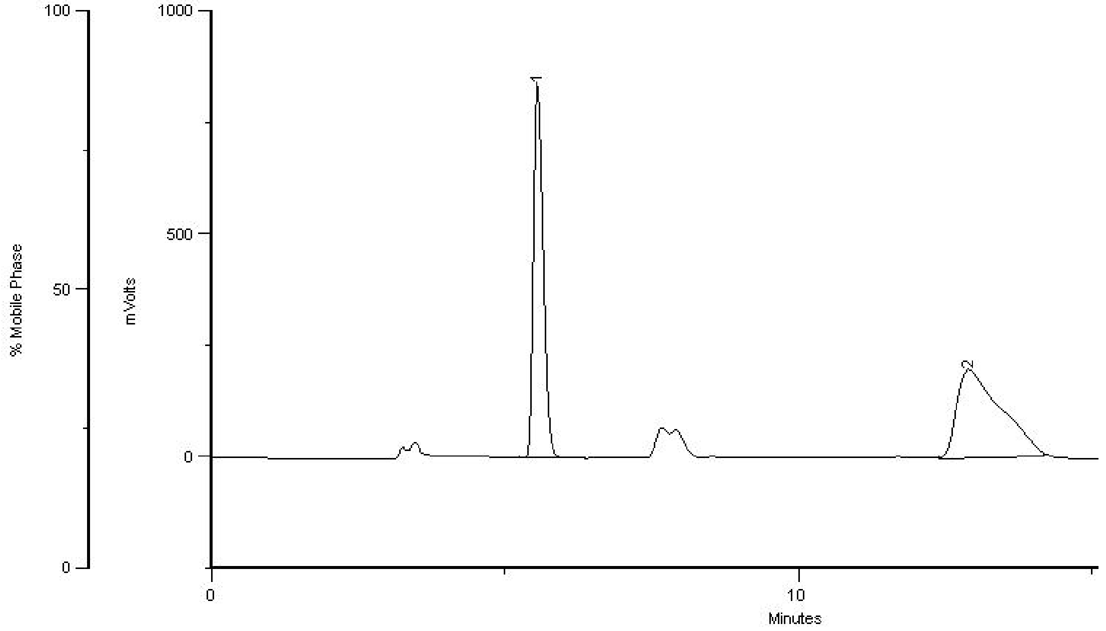

	R. Time	Area %			
1	5.55	48.02			
2	12.91	51.98			

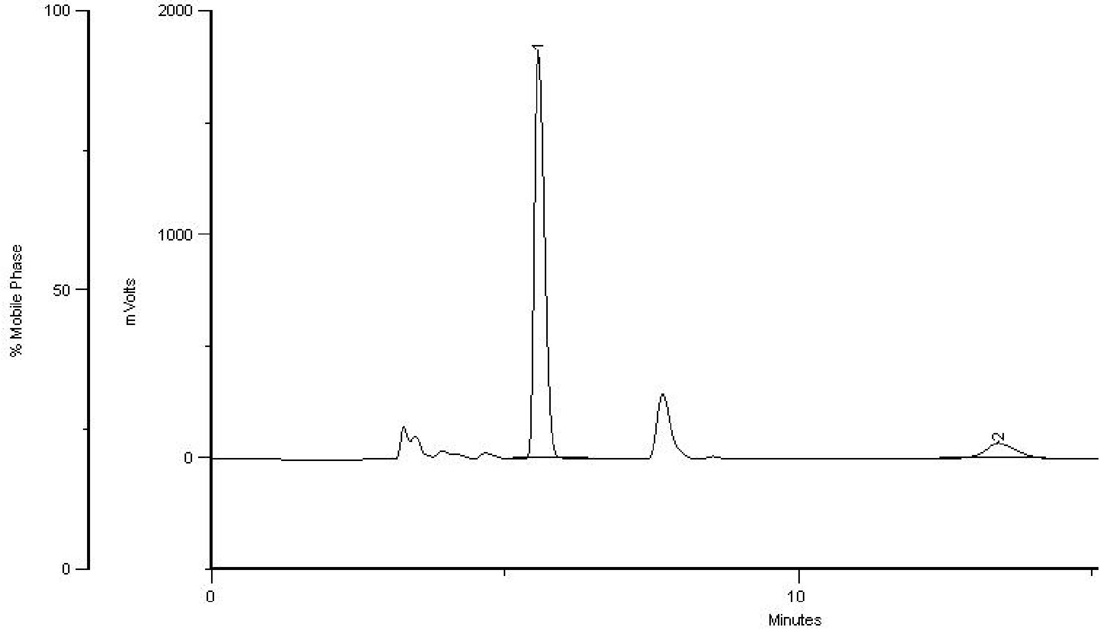

	R. Time	Area %			
1	5.57	88.76			
2	13.41	11.24			

(12)-*Syn*-(3*S*,4*S*)-3-methyl-3-phenyl-4-(trichloromethyl)oxetan-2-one

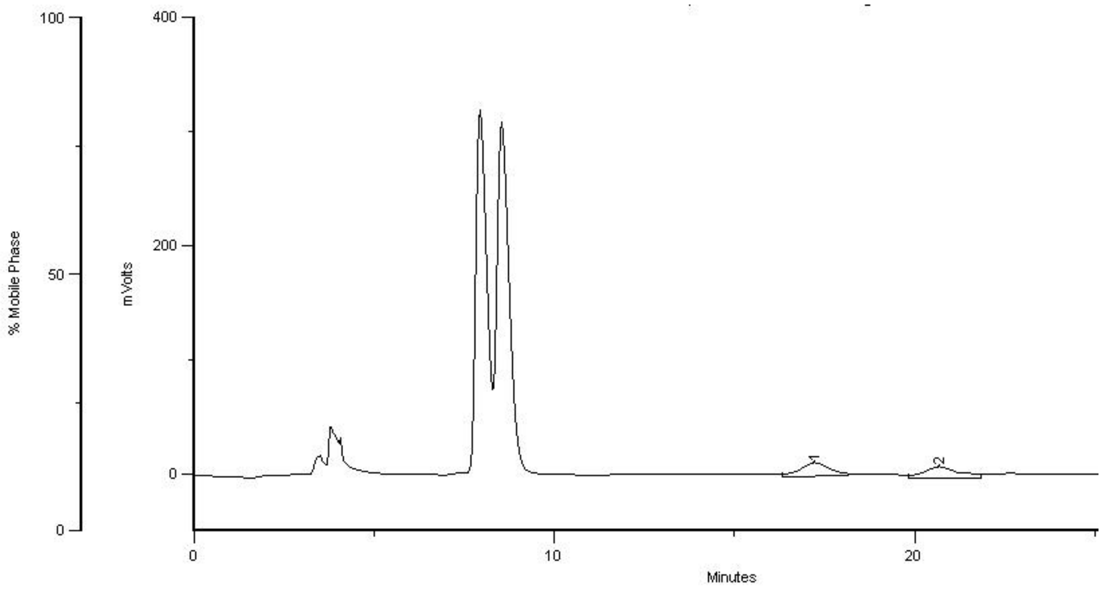

	R. Time	Area %			
1	17.22	50.01			
2	20.68	49.99			

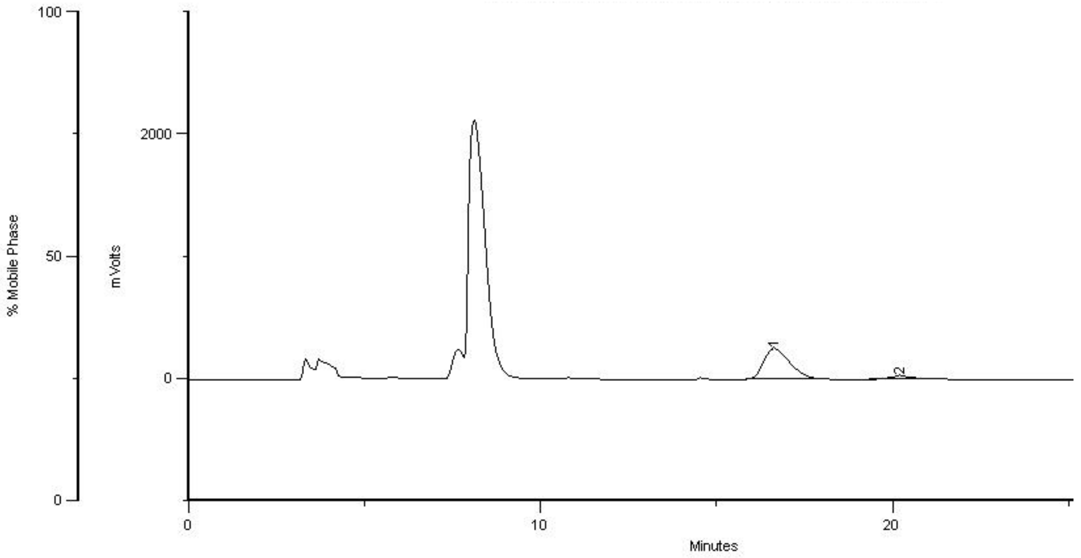

	R. Time	Area %			
1	16.64	89.00			
2	20.20	11.00			

(13)-*Anti*-(3*S*,4*R*)-3-butyl-3-phenyl-4-(trichloromethyl)oxetan-2-one

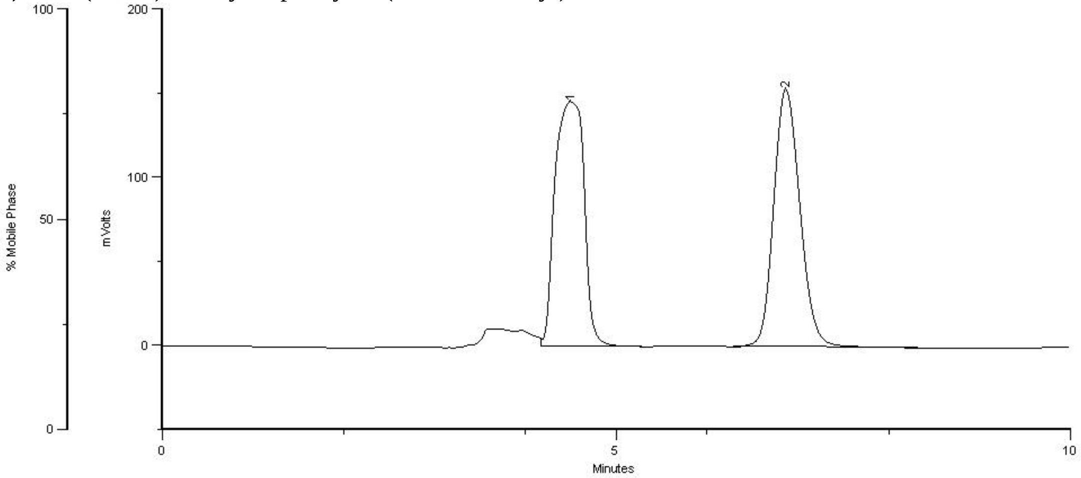

	R. Time	Area %			
1	4.50	51.13			
2	6.86	48.87			

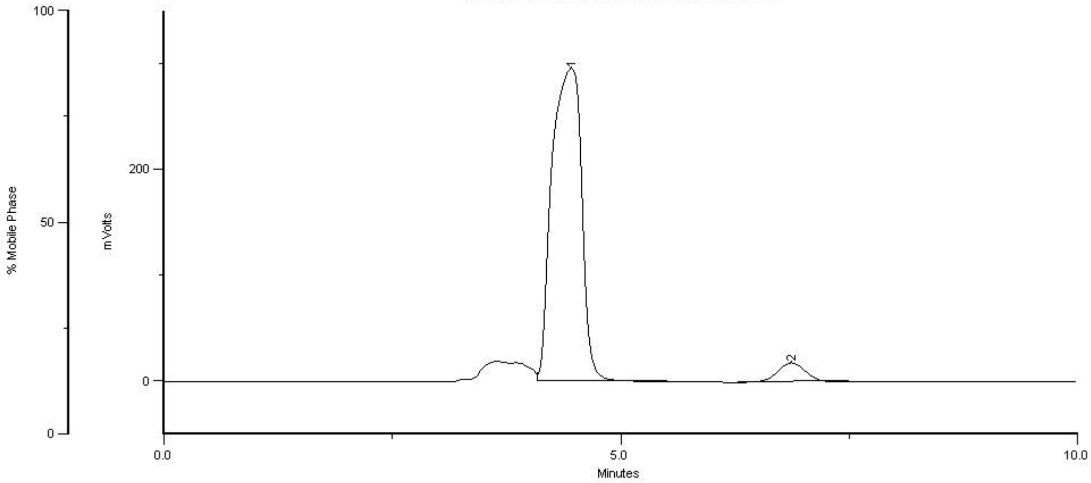

	R. Time	Area %			
1	4.46	94.32			
2	6.87	5.68			

(14)-*Syn*-(3*S*,4*R*)-3-isobutyl-3-phenyl-4-(trichloromethyl)oxetan-2-one

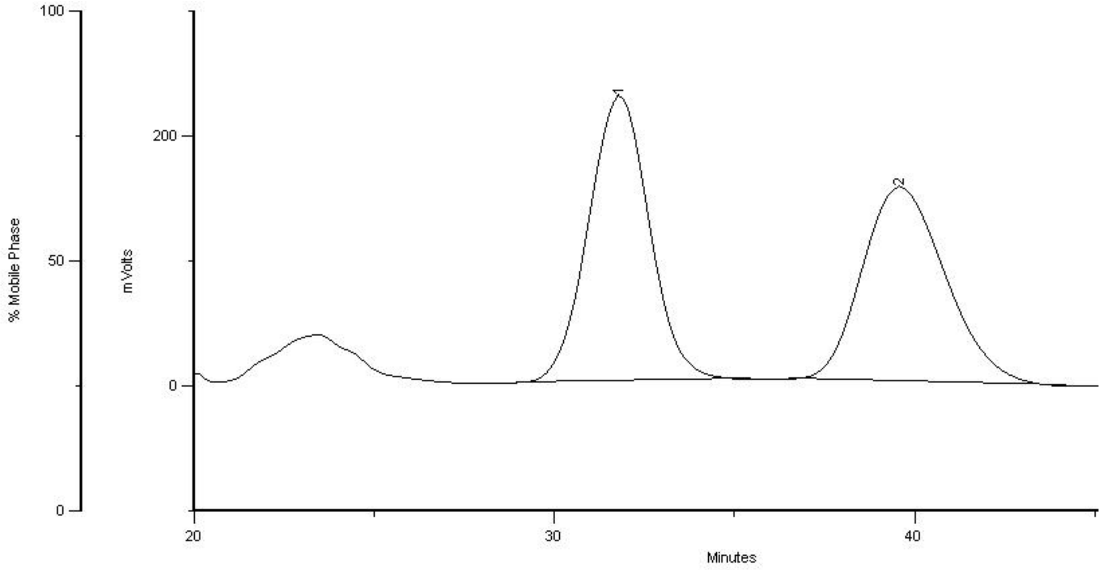

	R. Time	Area %			
1	31.81	51.70			
2	39.59	48.30			

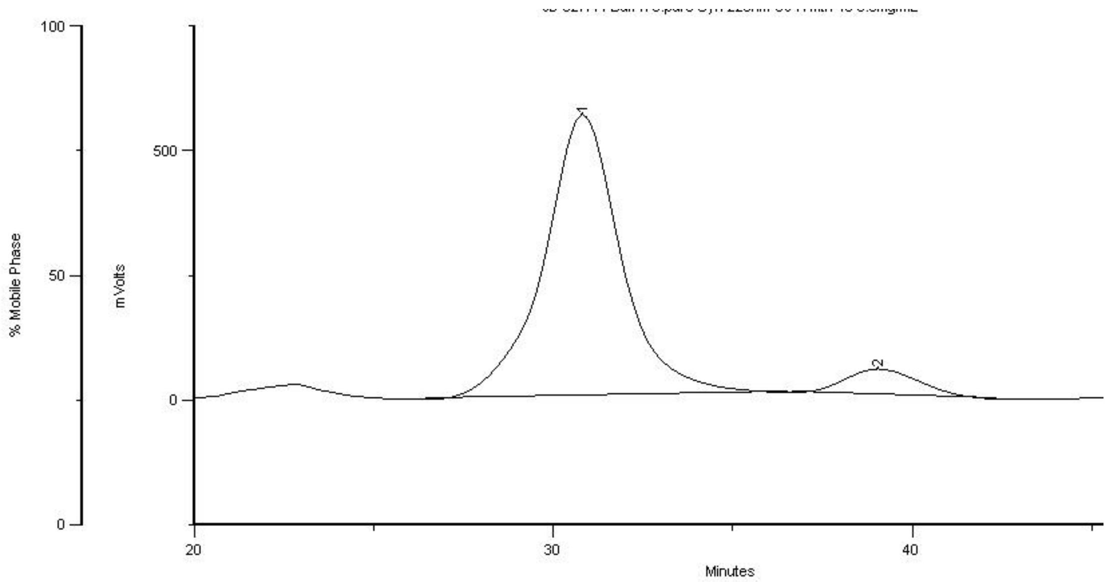

	R. Time	Area %			
1	30.81	91.83			
2	39.10	8.17			

(**16**)-(*S*)-2,2-Dichlorovinyl 2-chloro-3-methyl-2-phenylbutanoate

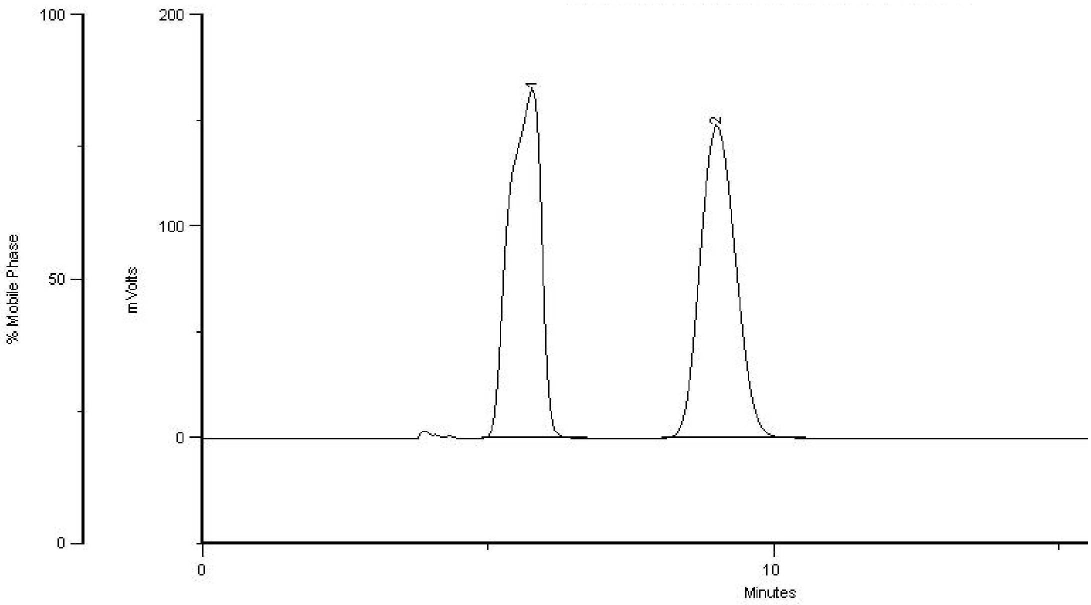

	R. Time	Area %			
1	5.78	49.89			
2	9.01	50.11			

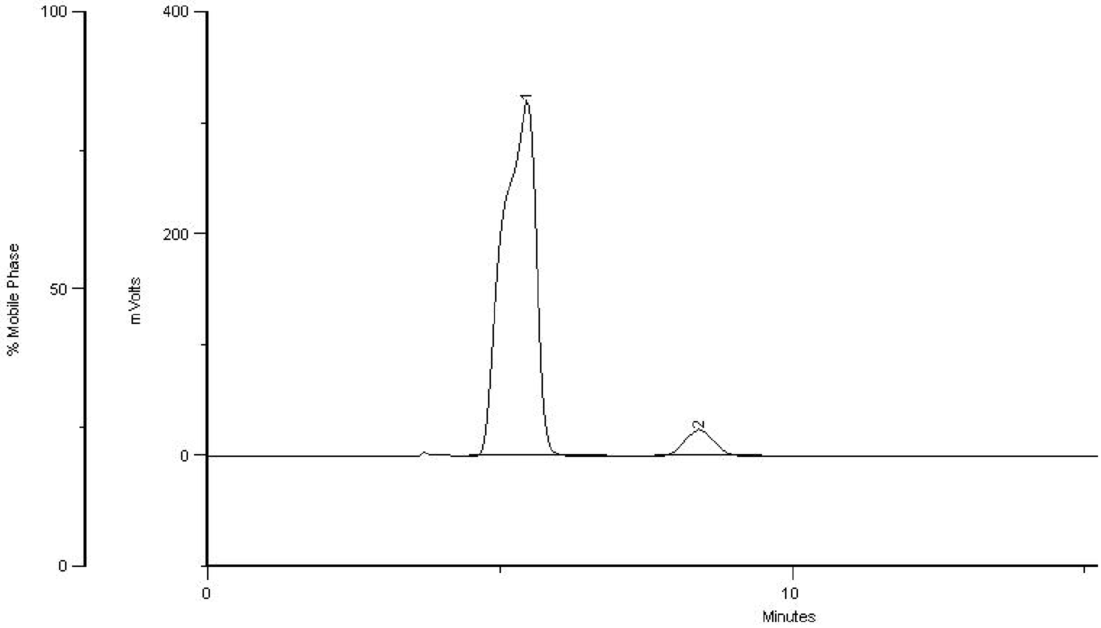

	R. Time	Area %			
1	5.46	93.52			
2	8.41	6.48			

(17)-(*S*)-2,2-Dichlorovinyl 2-chloro-2-(2-tolyl)propanoate

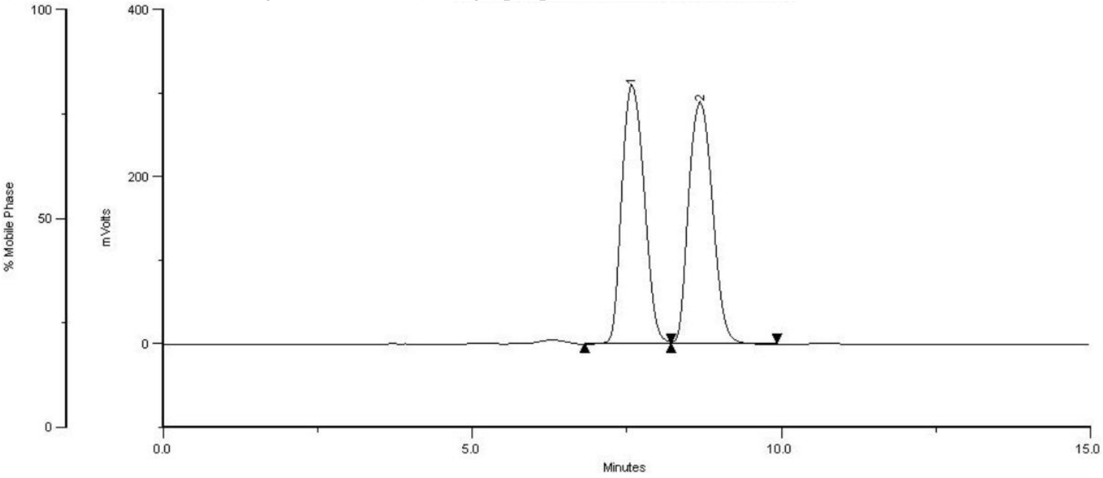

	R. Time	Area %				
1	7.58	50.01				
2	8.68	49.99				

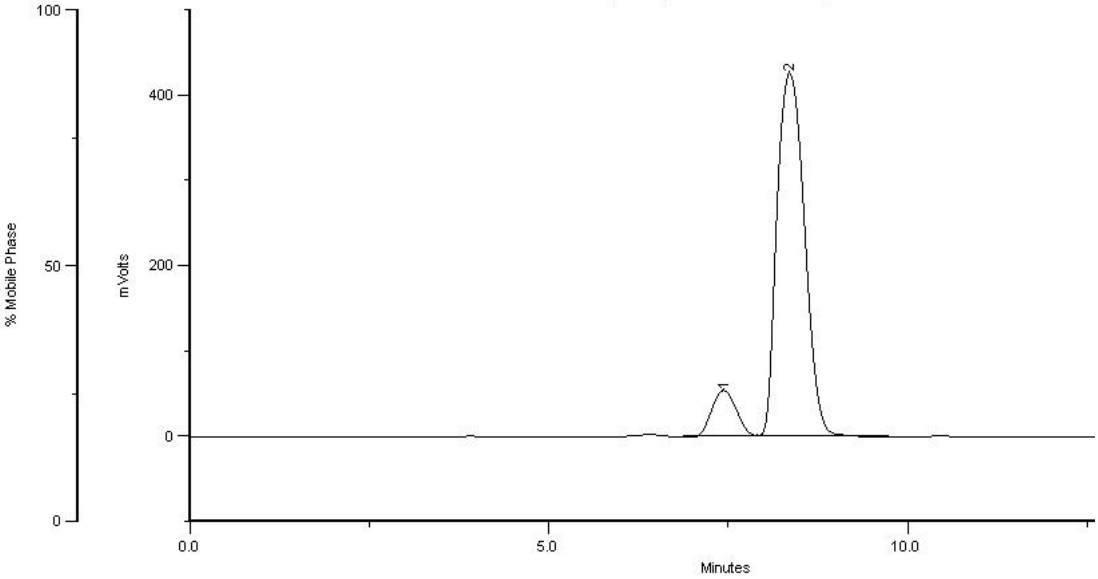

	R. Time	Area %			
1	7.43	10.33			
2	8.36	89.67			

(18)-(*S*)-2,2-Dichlorovinyl 2-chloro-2-(2-tolyl)butanoate

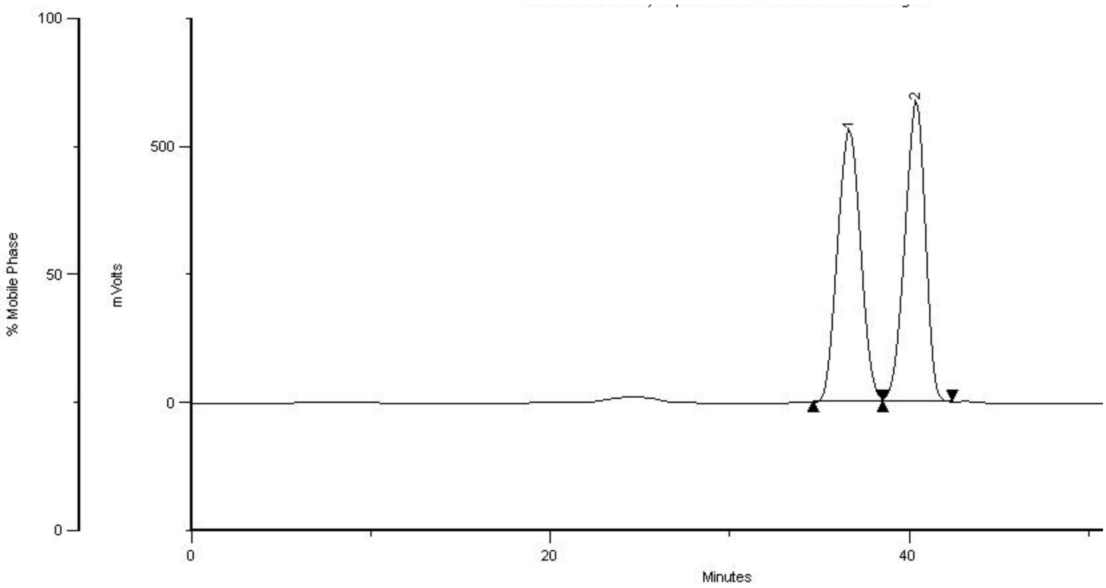

	R. Time	Area %			
1	36.66	50.68			
2	40.39	49.32			

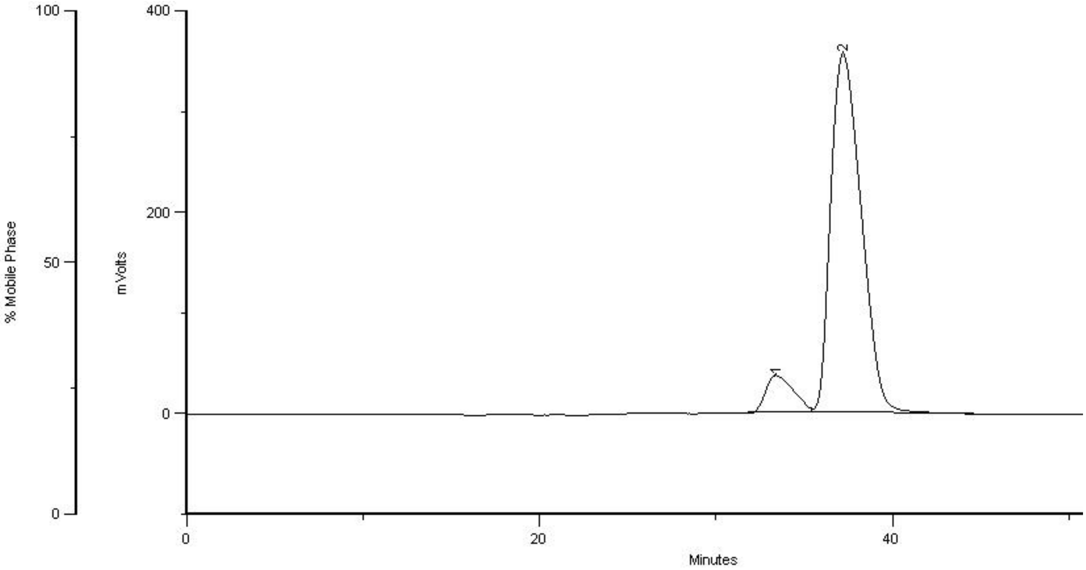

	R. Time	Area %			
1	33.40	8.87			
2	37.20	91.13			

(19)-(*S*)-2,2-Dichlorovinyl 2-chloro-2-(naphthalene-1-yl)propanoate

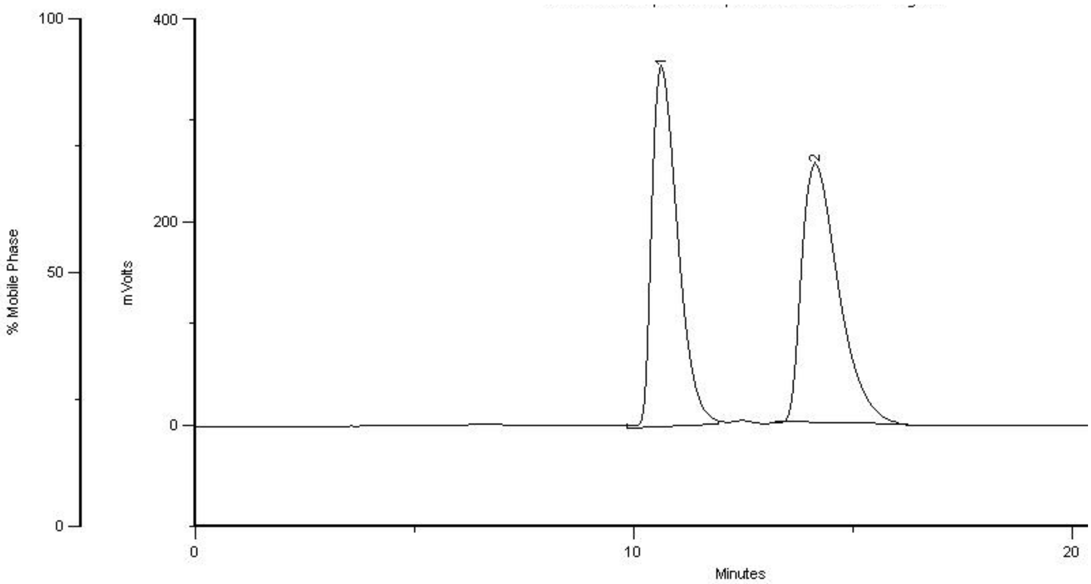

	R. Time	Area %			
1	10.63	49.35			
2	14.14	50.65			

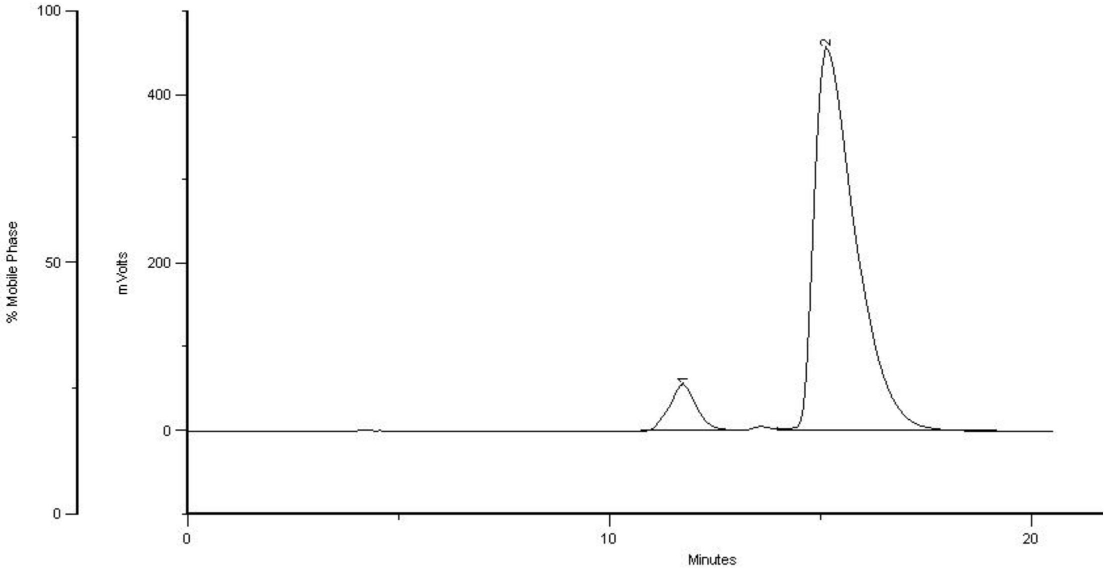

	R. Time	Area %			
1	11.74	7.54			
2	15.15	92.46			

(20)-(*S*)-2,2-Dichlorovinyl 2-chloro-2-(2-chlorophenyl)butanoate

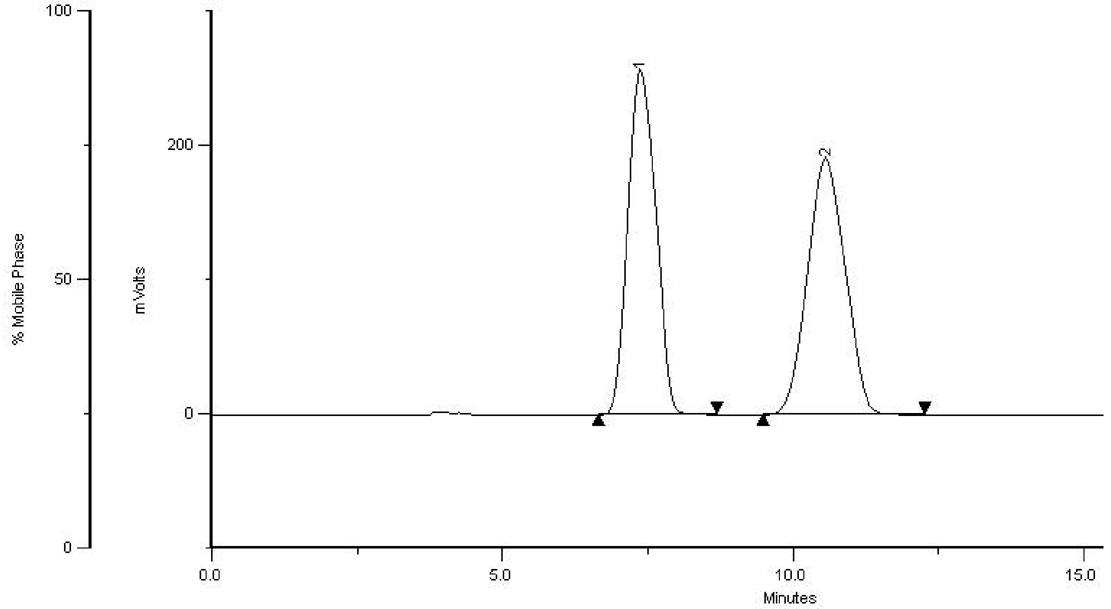

	R. Time	Area %			
1	7.37	49.96			
2	10.56	50.04			

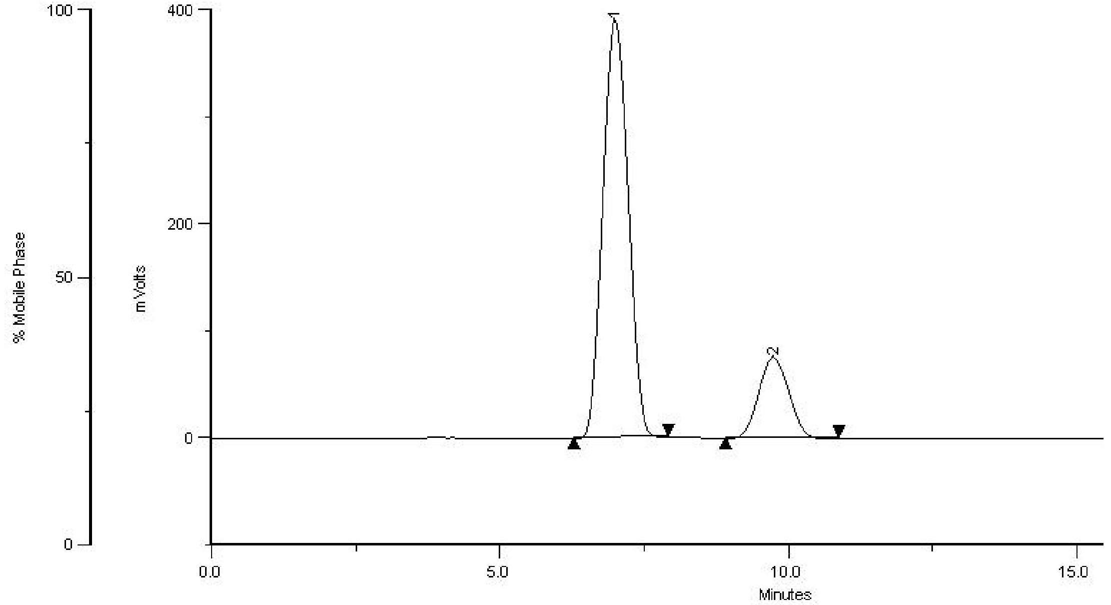

	R. Time	Area %			
1	7.00	81.14			
2	9.74	18.86			

(21)-(*S*)-2,2-Dichlorovinyl 2-chloro-2-(naphthalene-1-yl)hexanoate

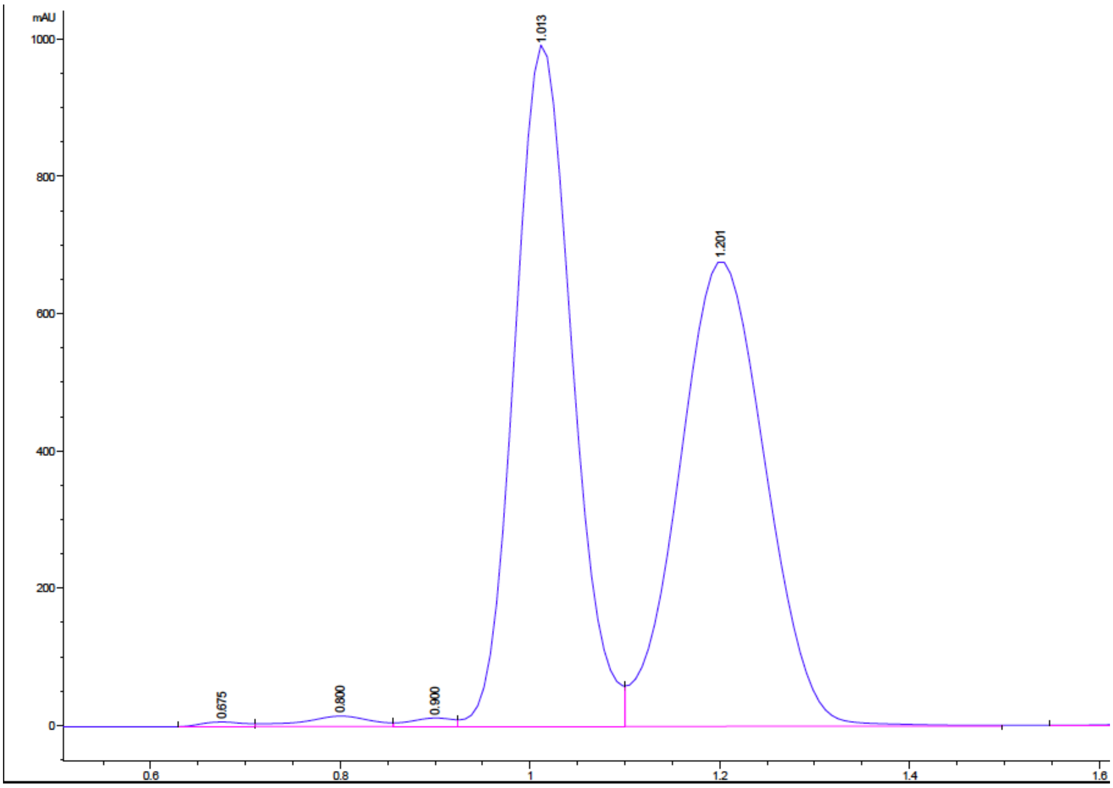

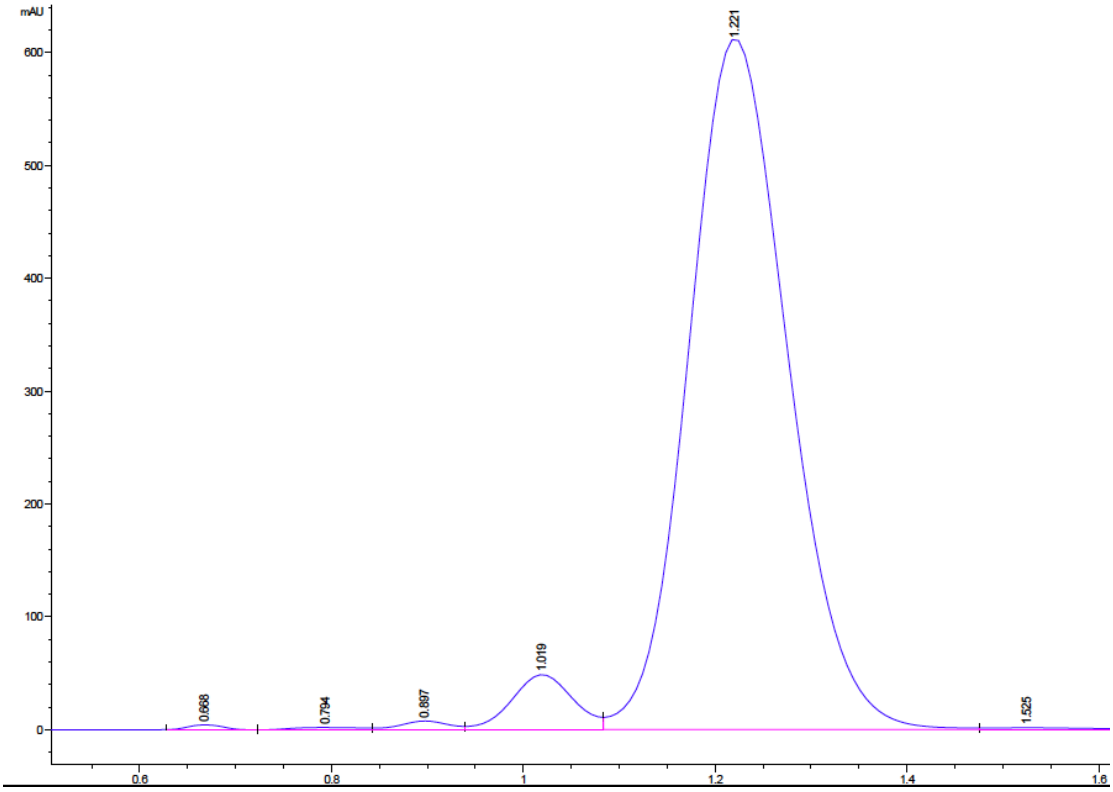

(**28**)-3-Ethyl-3-(2-methoxyphenyl)-4-(trichloromethyl)oxetan-2-one

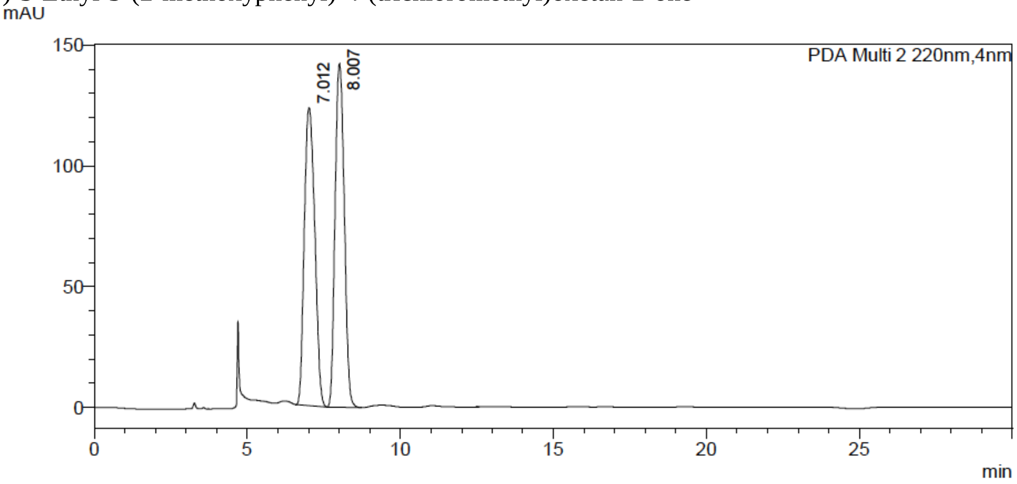

<Peak Table>

PDA Ch2 220nm

Peak#	Ret. Time	Area%
1	7.012	49.651
2	8.007	50.349
Total		100.000

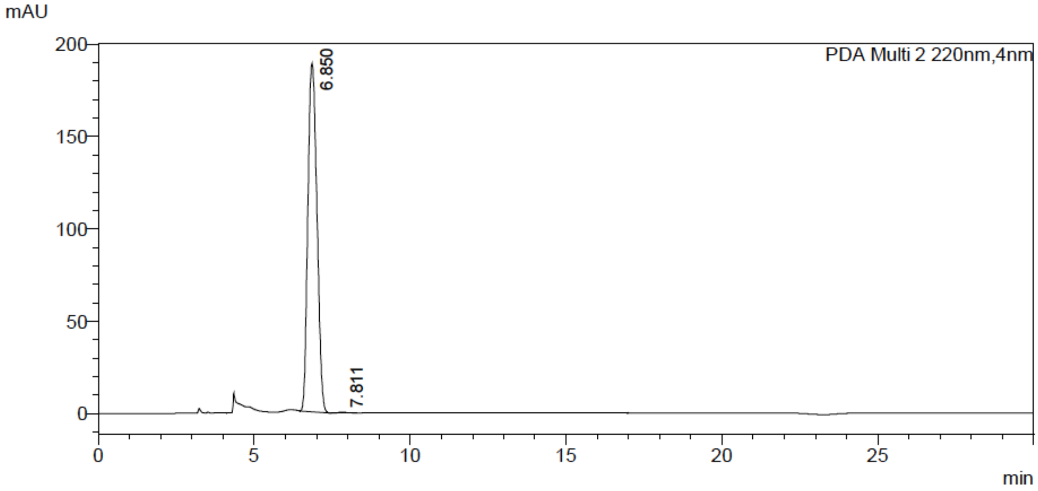

<Peak Table>

PDA Ch2 220nm

Peak#	Ret. Time	Area%
1	6.850	99.646
2	7.811	0.354
Total		100.000

NMR Spectrum

(S1)-2-(naphthalen-2-yl)propanoyl chloride

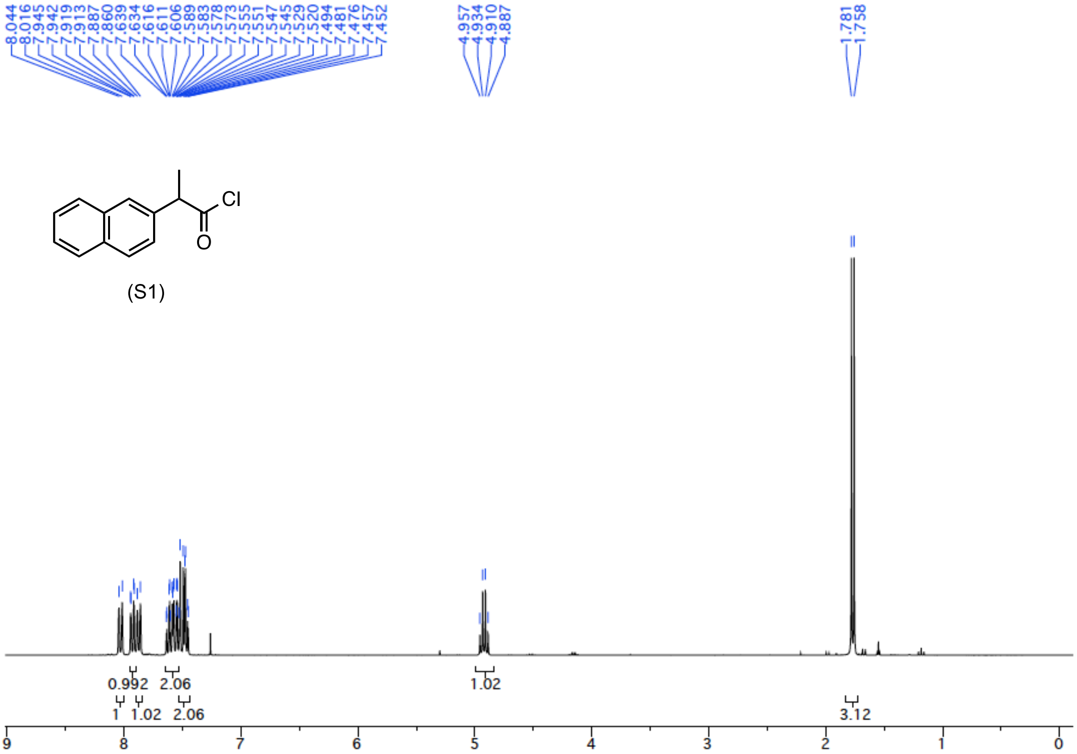

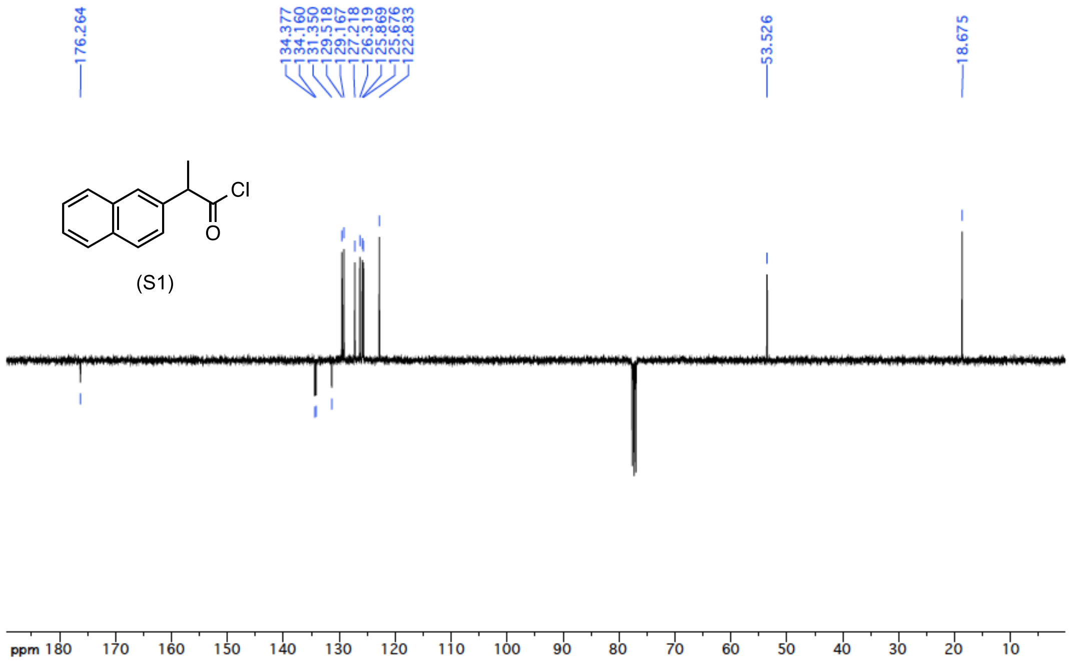

(S2) 2-(naphthalen-2-yl)hexanoyl chloride

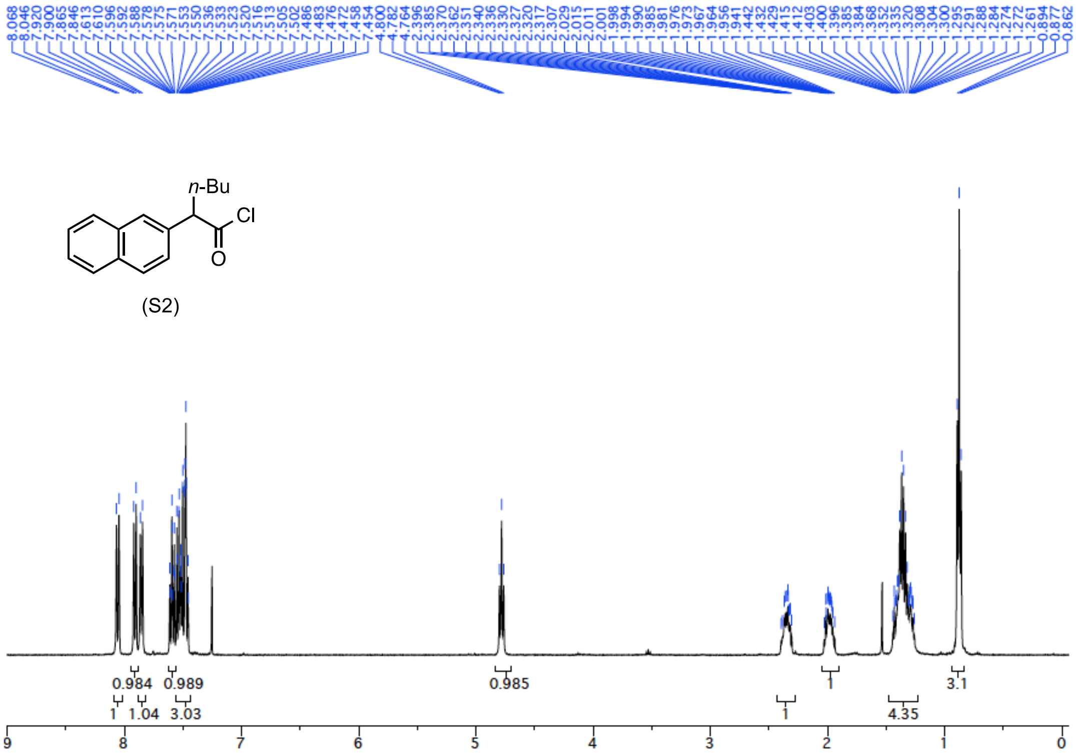

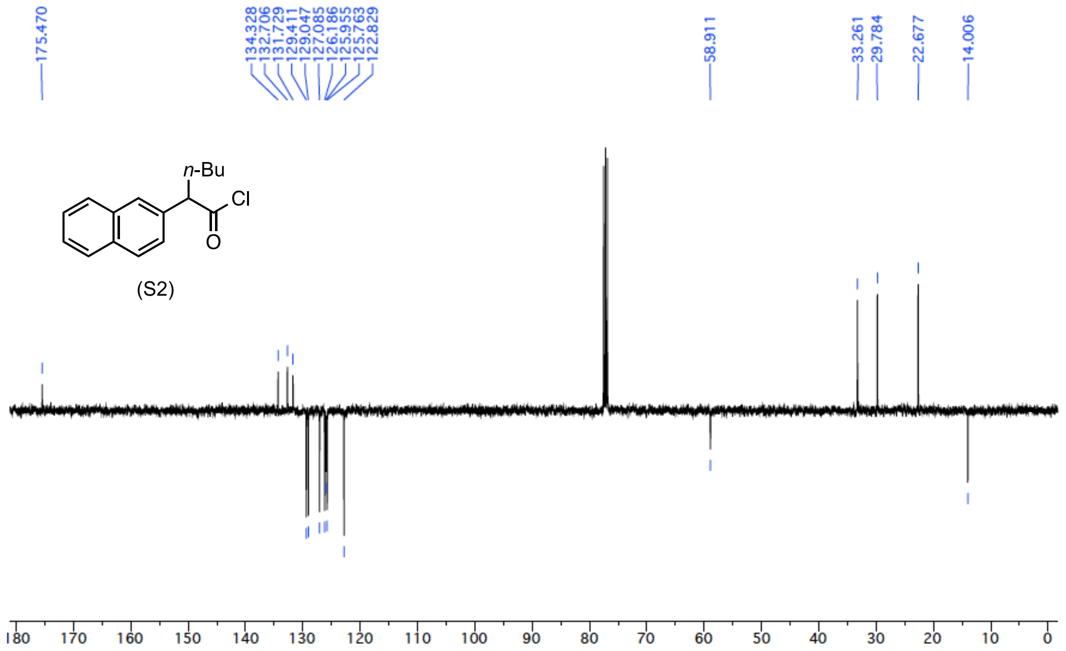

(S3)–Ethyl-2-tolylketene

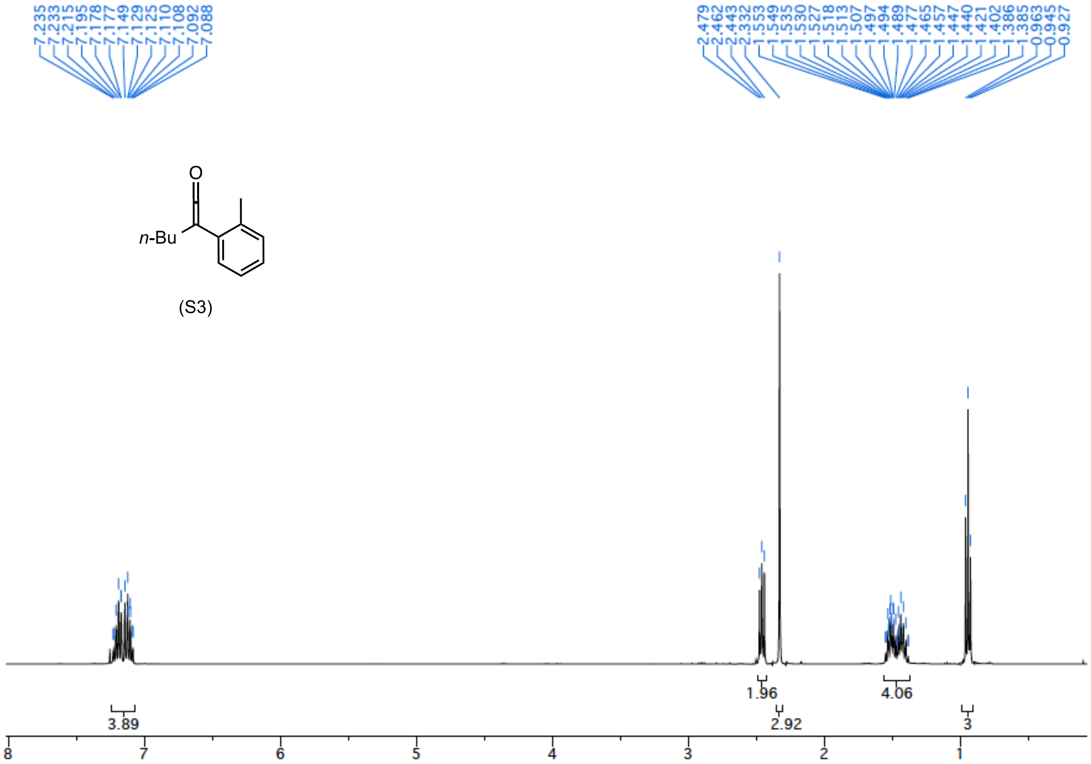

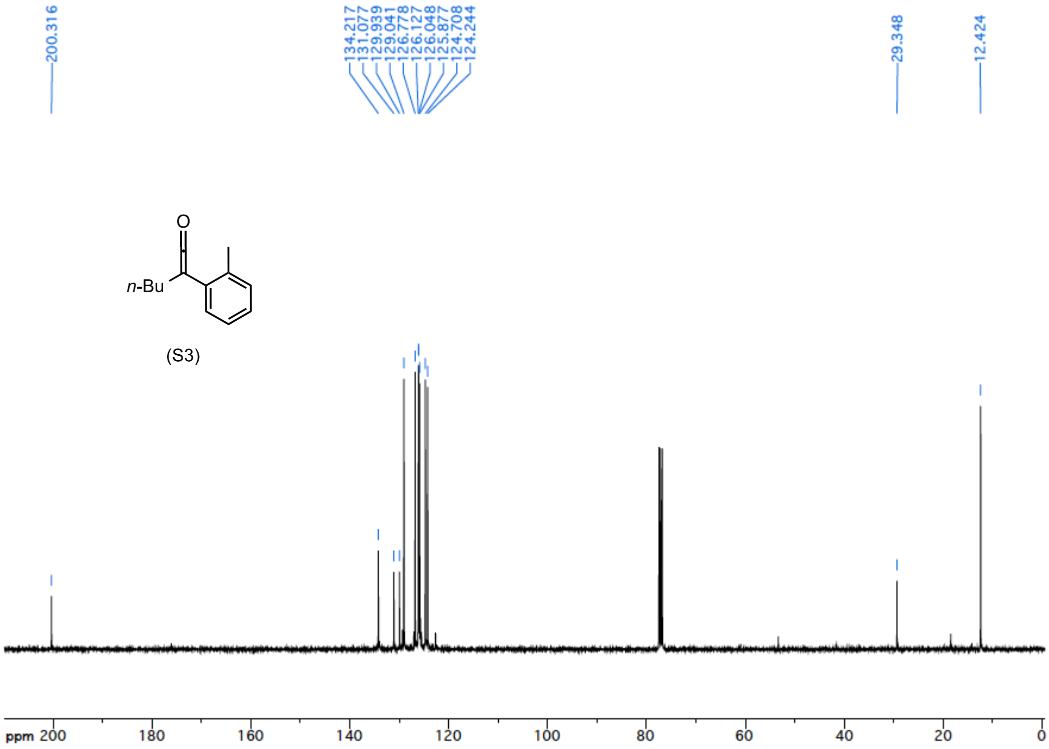

(S4)-Methyl-2-napthyl ketene

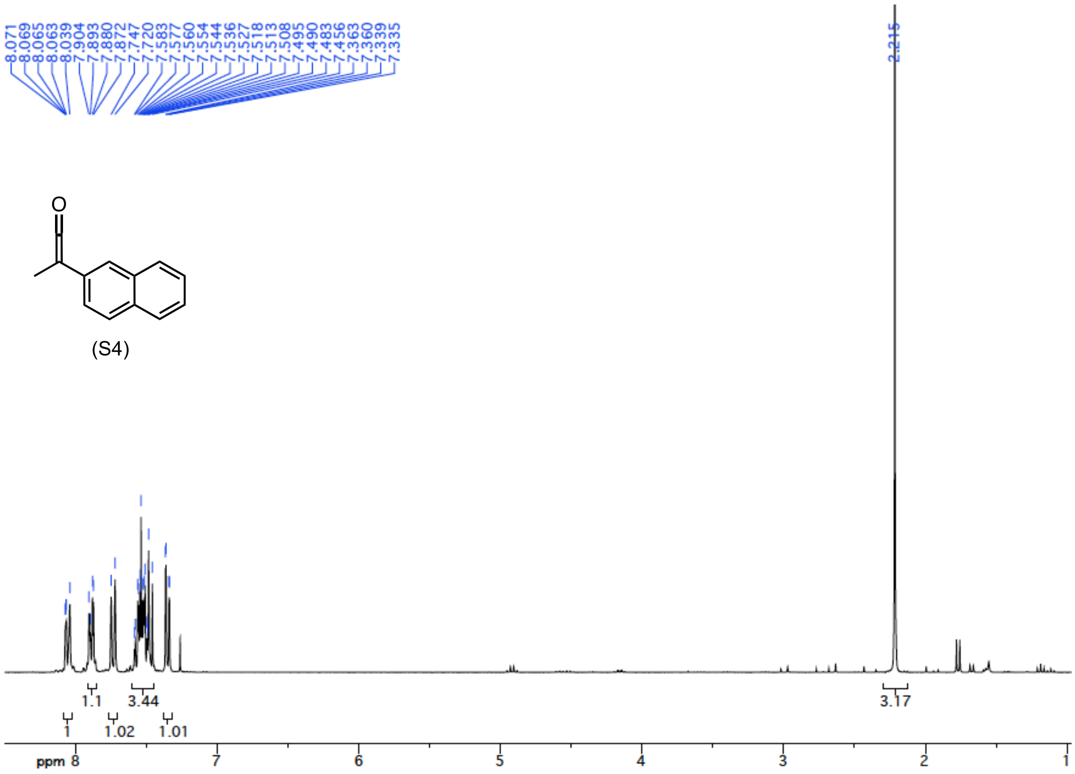

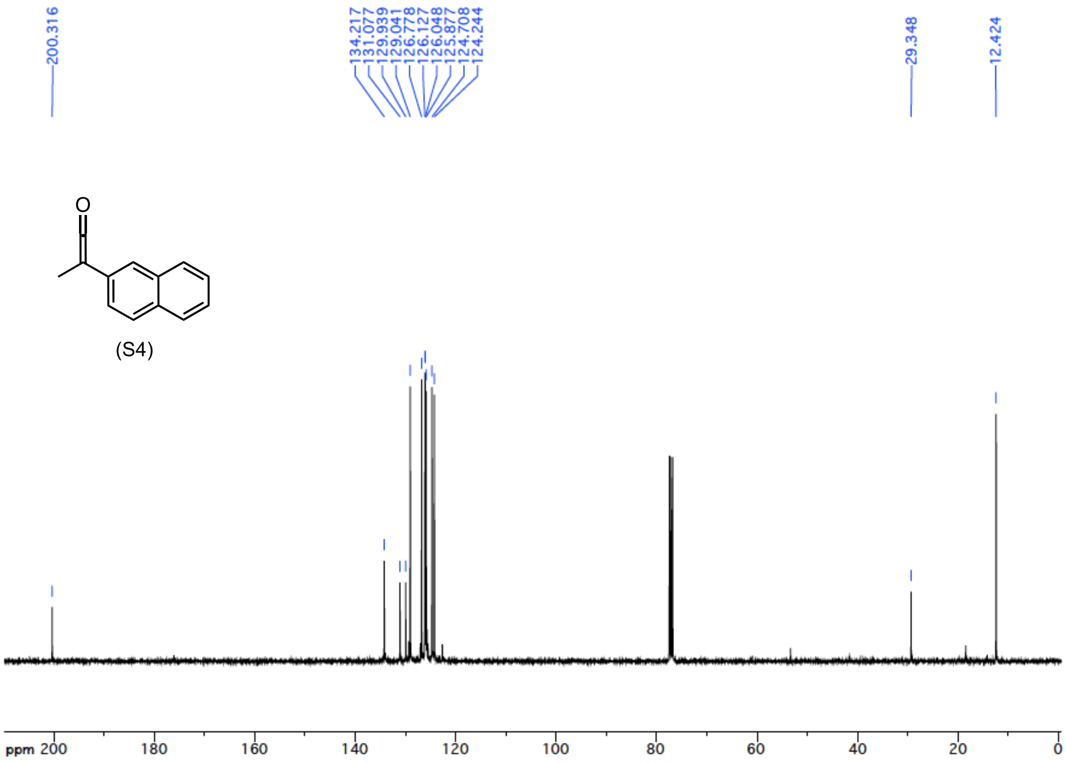

(S5)-Butyl-2-napthyl ketene

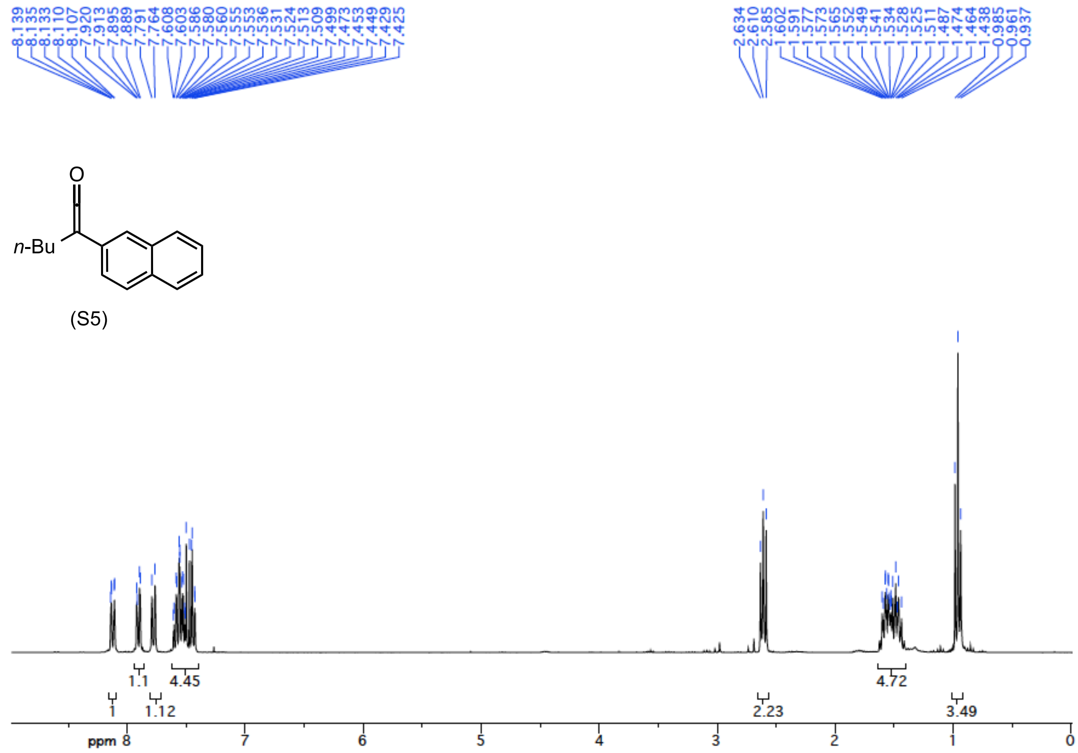

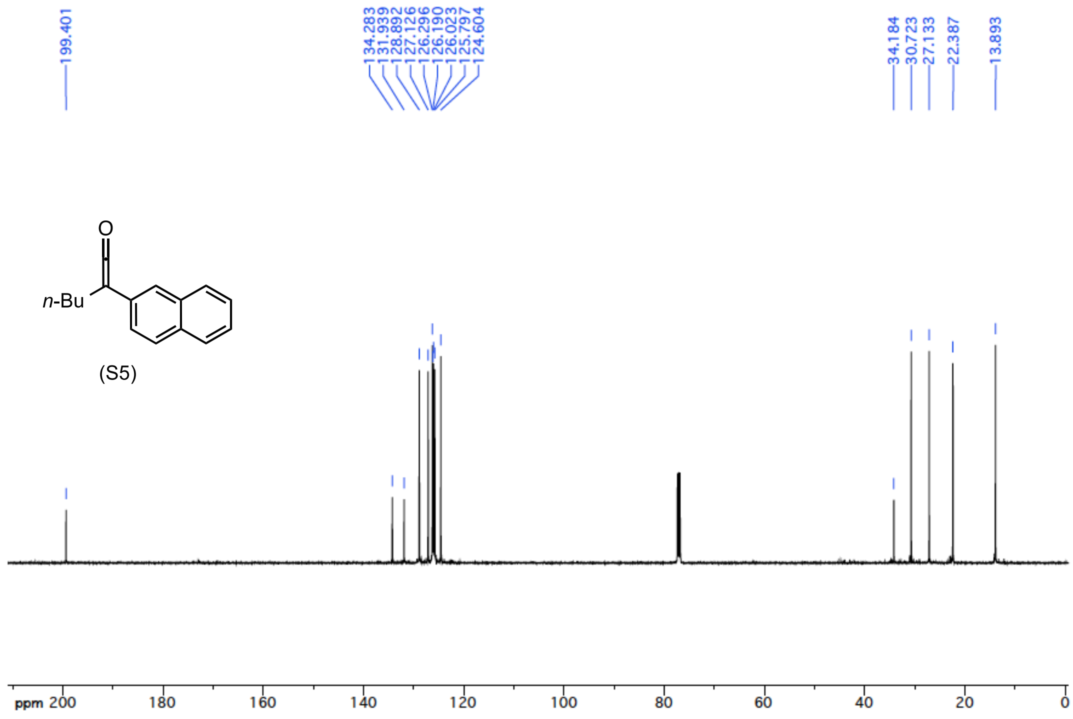

(2)-*Anti*-(3*S*,4*R*)-3-ethyl-3-phenyl-4-(trichloromethyl)oxetan-2-one

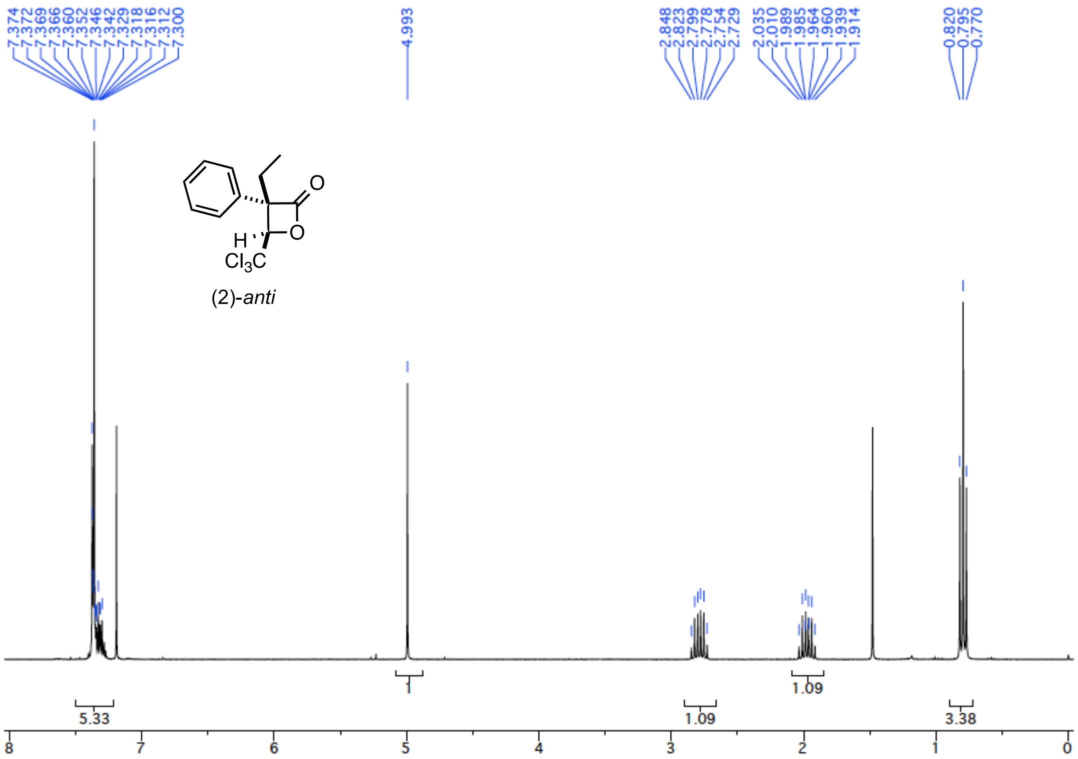

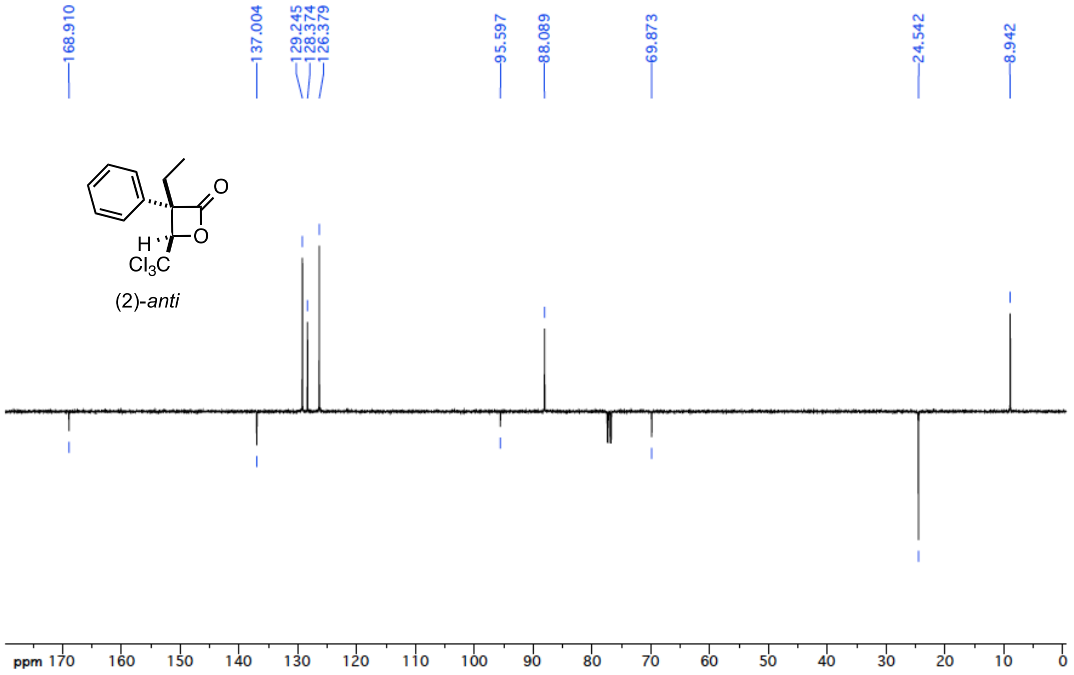

(3)-*Syn*-(3*S*,4*R*)-3-ethyl-3-phenyl-4-(trichloromethyl)oxetan-2-one

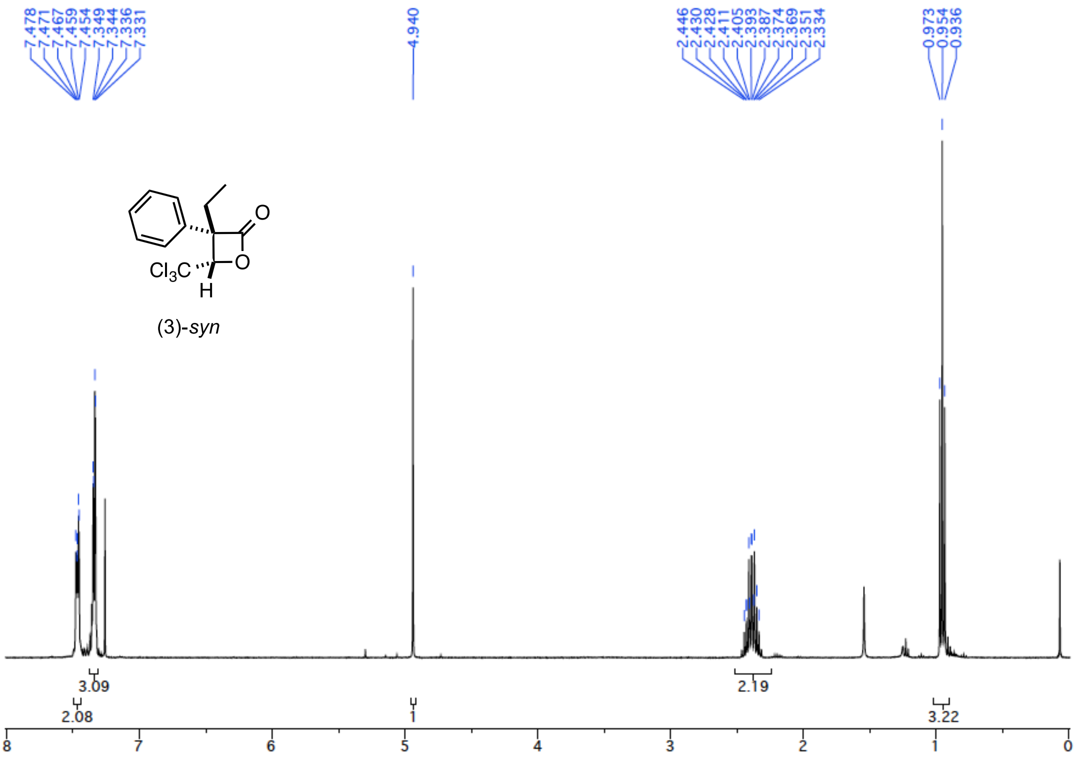

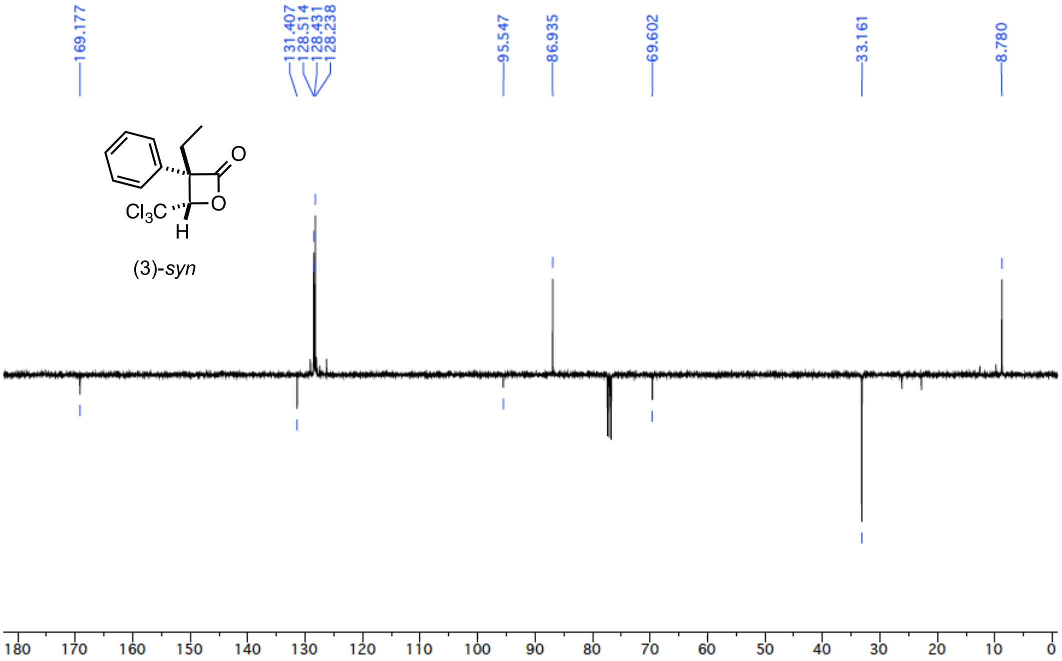

(5)-(*S*)-2,2-Dichlorovinyl 2-chloro-2-(naphthalene-1-yl)butanoate

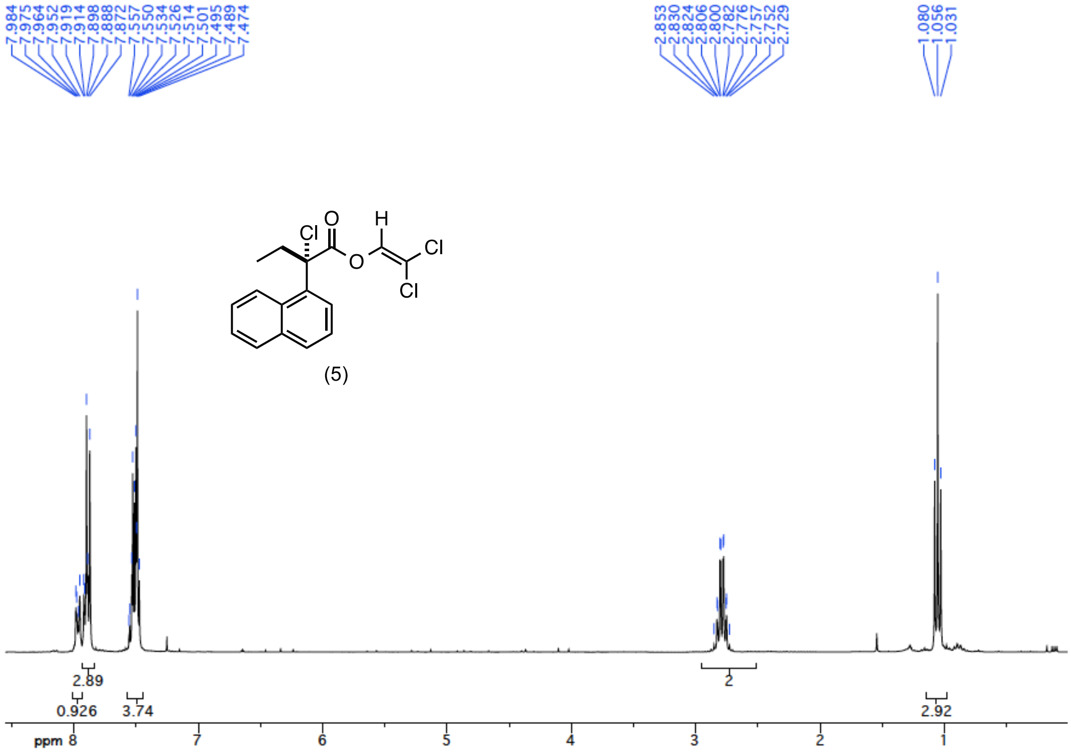

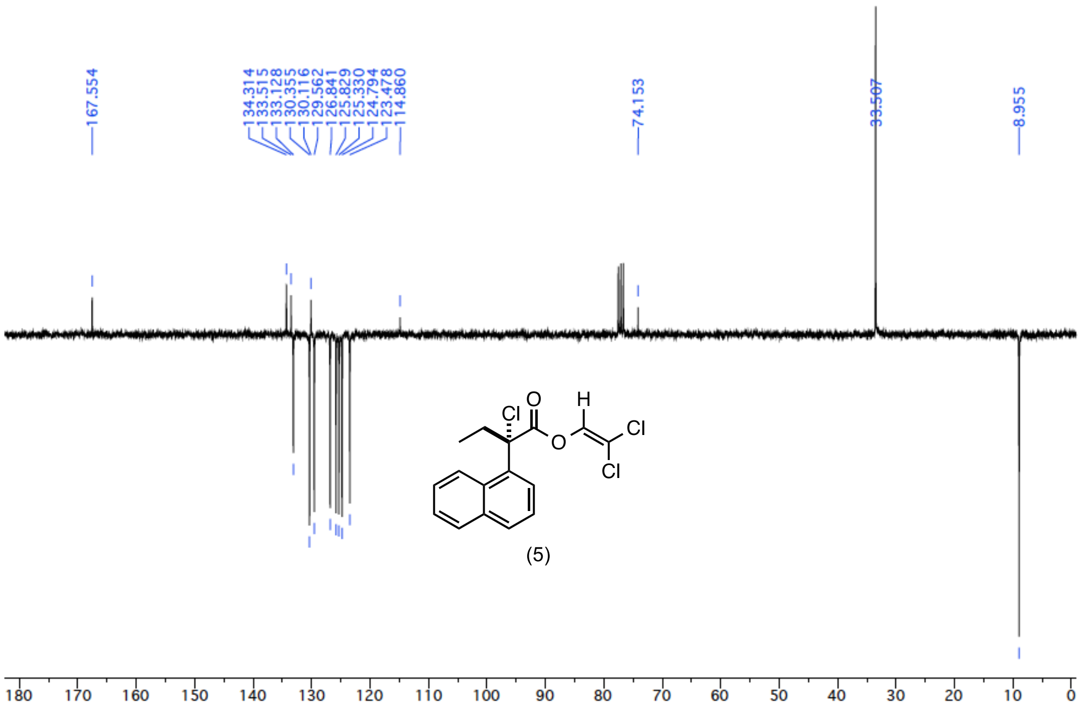

(6)-(*S*)-2-chloro-2-(naphthalen-1-yl)-*N*-((*S*)-1-phenylethyl)butanamide

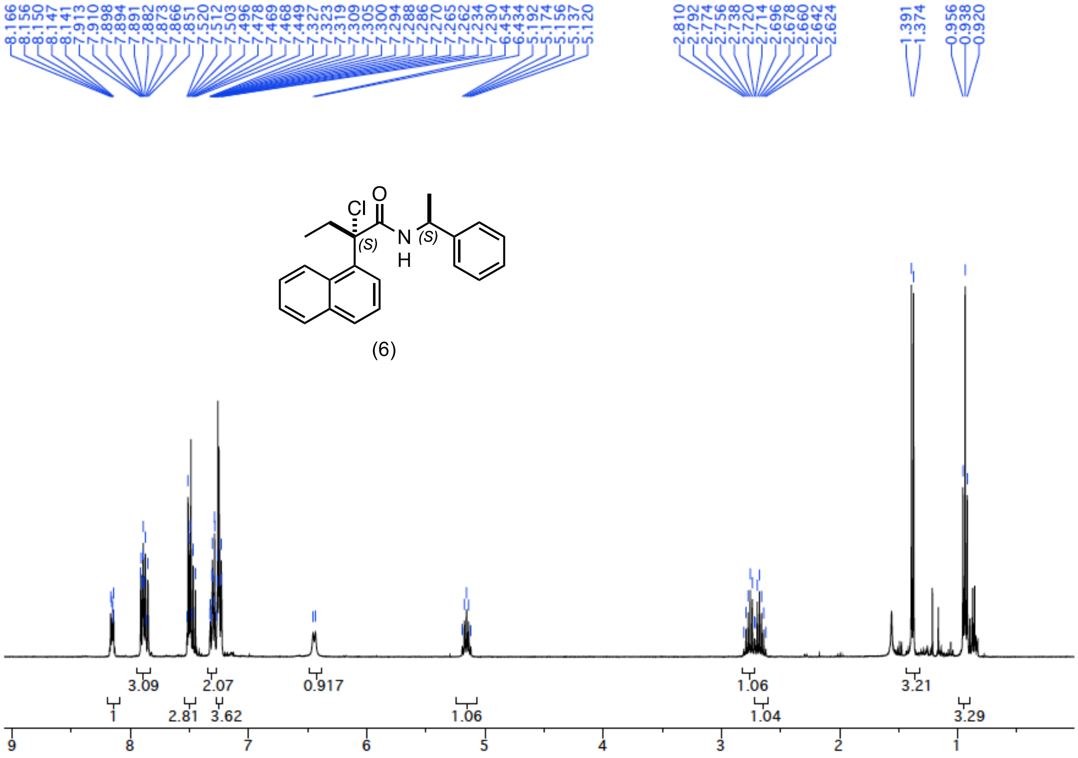

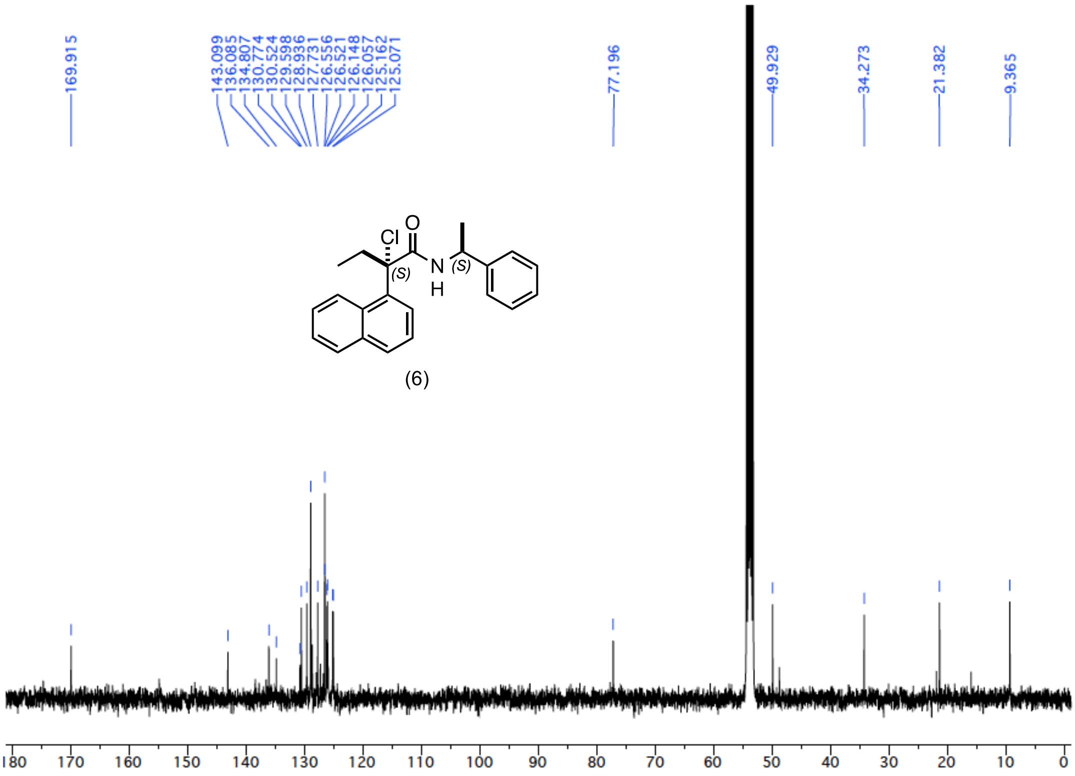

(8)-*Anti*-(3*S*,4*R*)-3-(4-methoxyphenyl)-3-ethyl-4-(trichloromethyl)oxetan-2-one

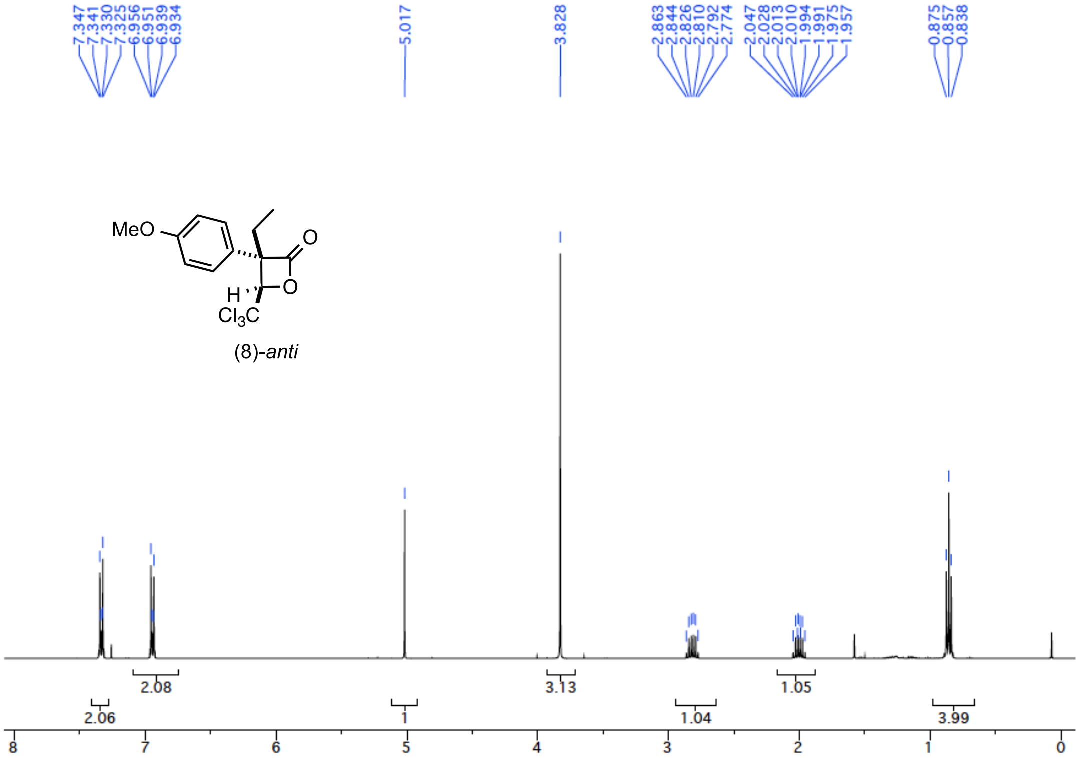

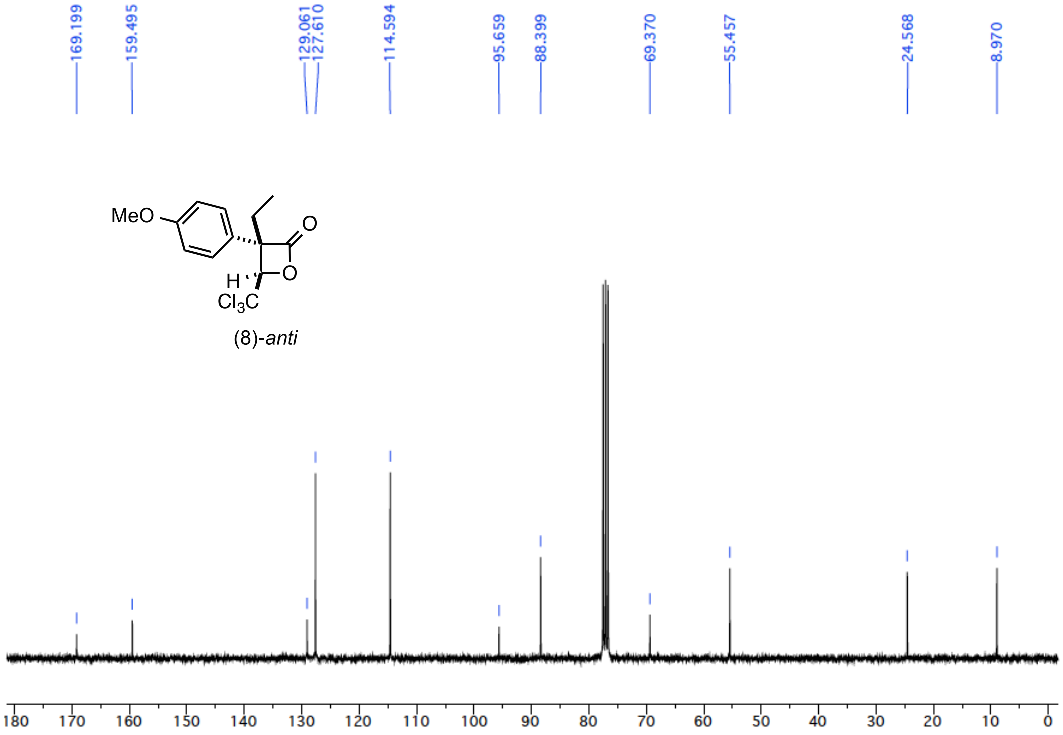

(**8**)-*Syn*-(3*S*,4*S*)-3-(4-methoxyphenyl)-3-ethyl-4-(trichloromethyl)oxetan-2-one

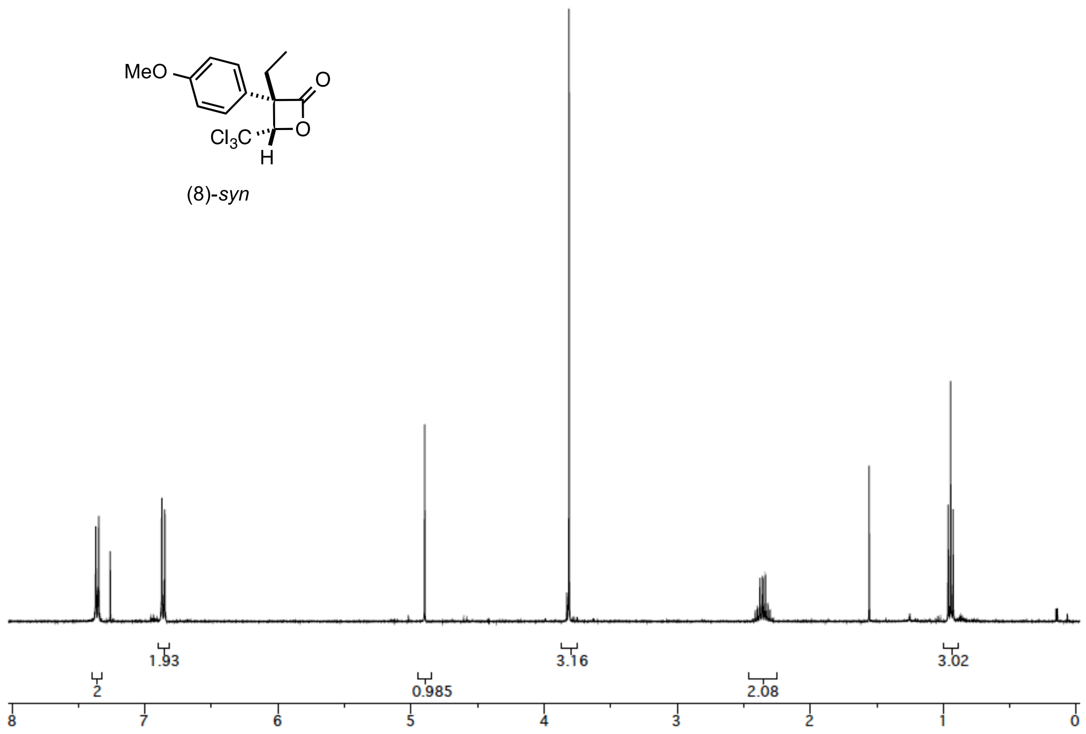

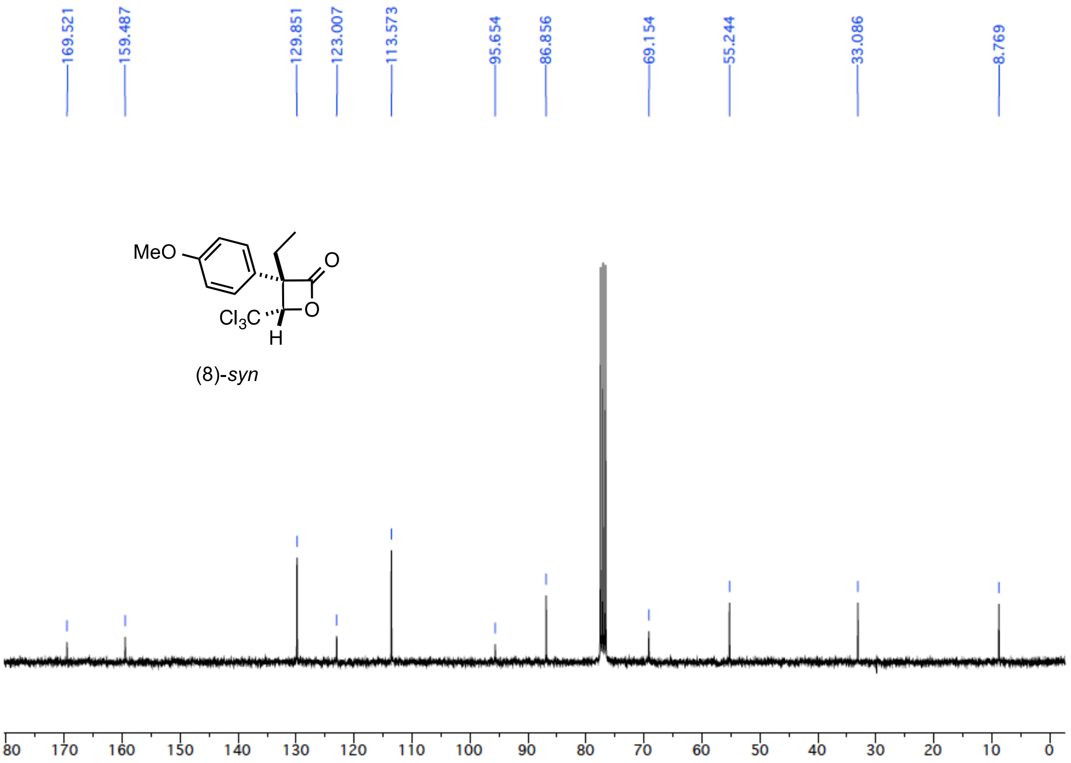

(9)-*Anti*-(3*S*,4*R*)-3-(4-fluorophenyl)-3-ethyl-4-(trichloromethyl)oxetan-2-one

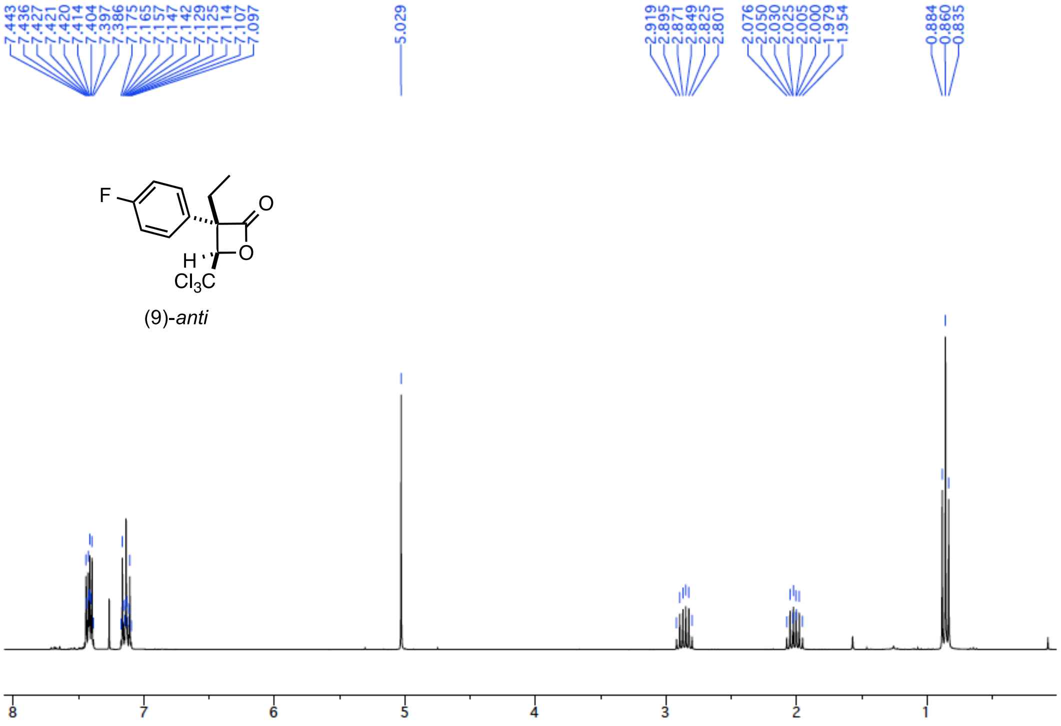

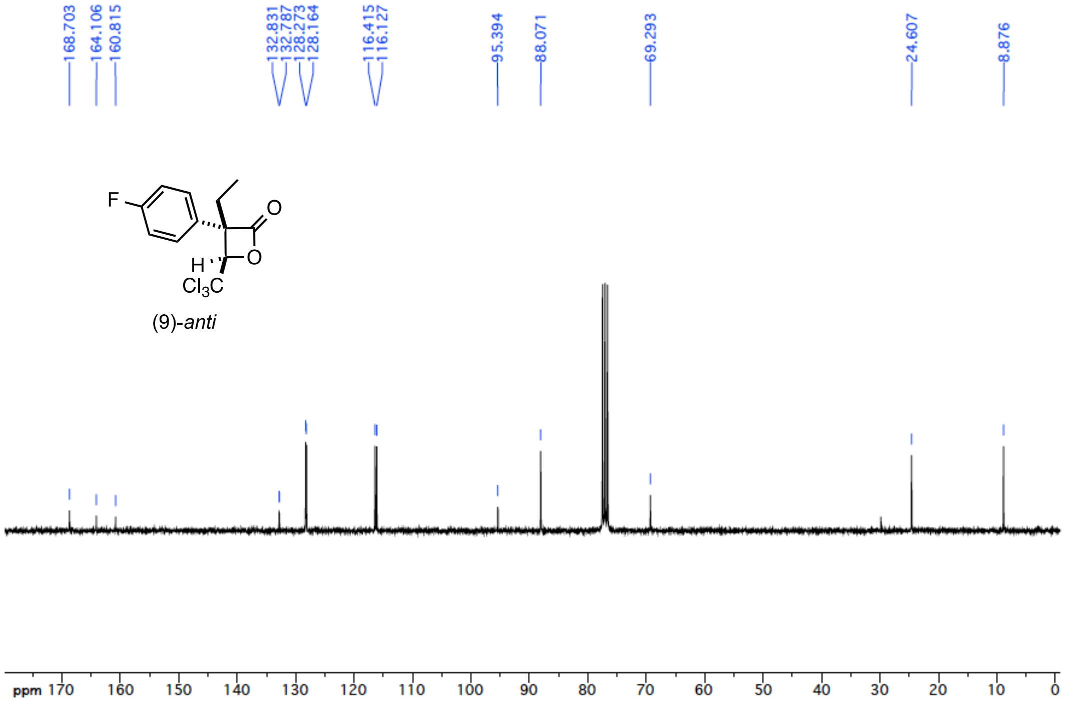

(9)-*Syn*-(3*S*,4*S*)-3-(4-fluorophenyl)-3-ethyl-4-(trichloromethyl)oxetan-2-one

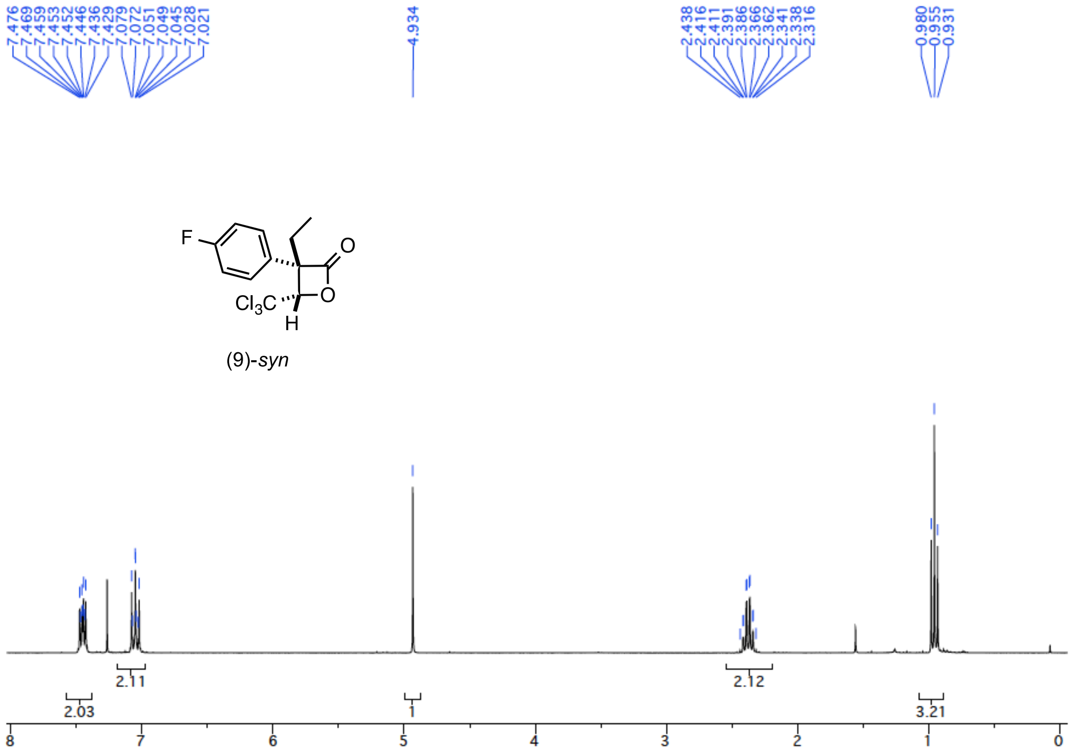

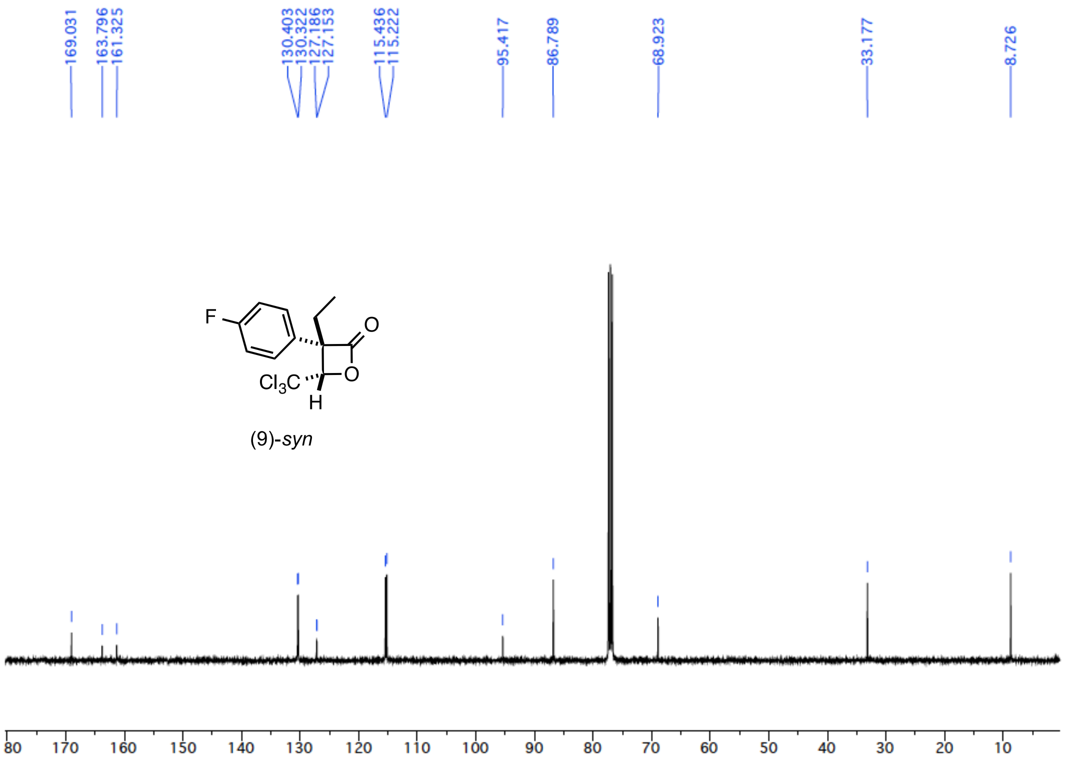

(10)-*Anti*-(3*S*,4*R*)-3-(4-chlorophenyl)-3-ethyl-4-(trichloromethyl)oxetan-2-one

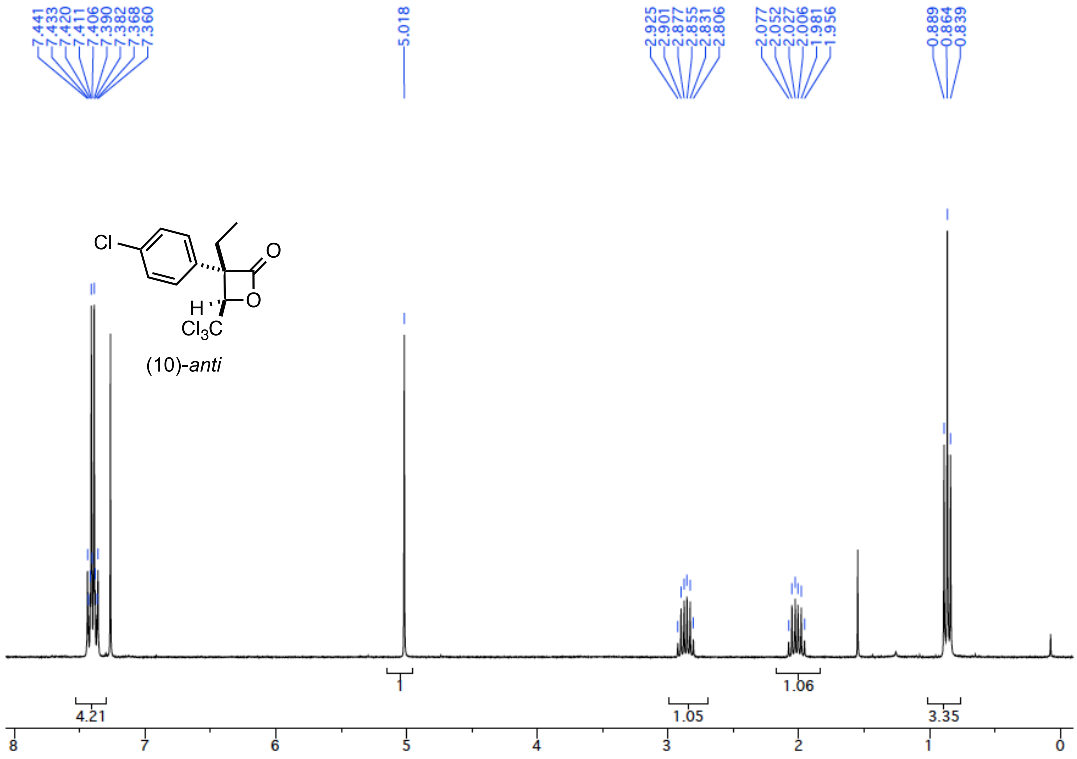

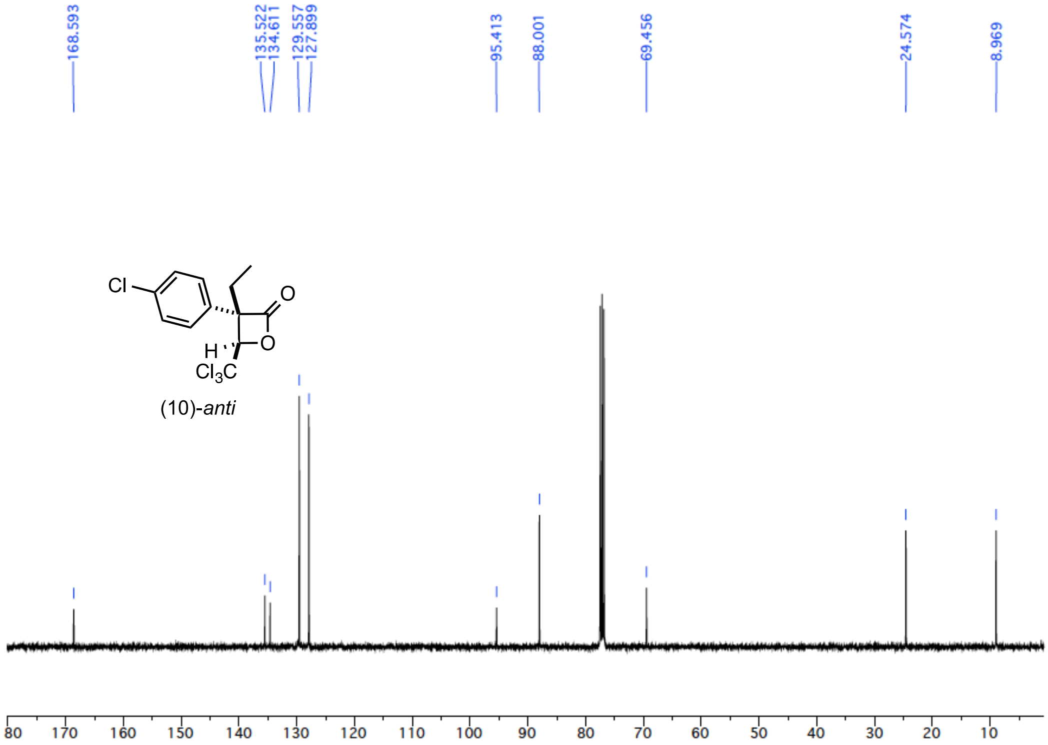

(10)-*Syn*-(3*S*,4*S*)-3-(4-chlorophenyl)-3-ethyl-4-(trichloromethyl)oxetan-2-one

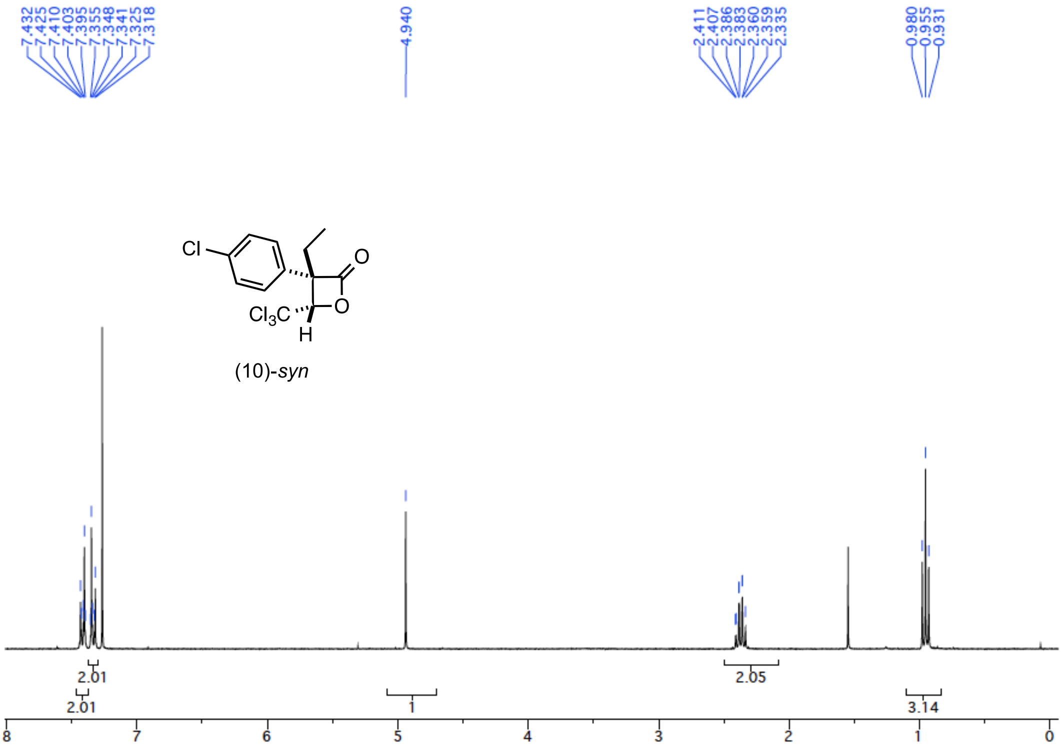

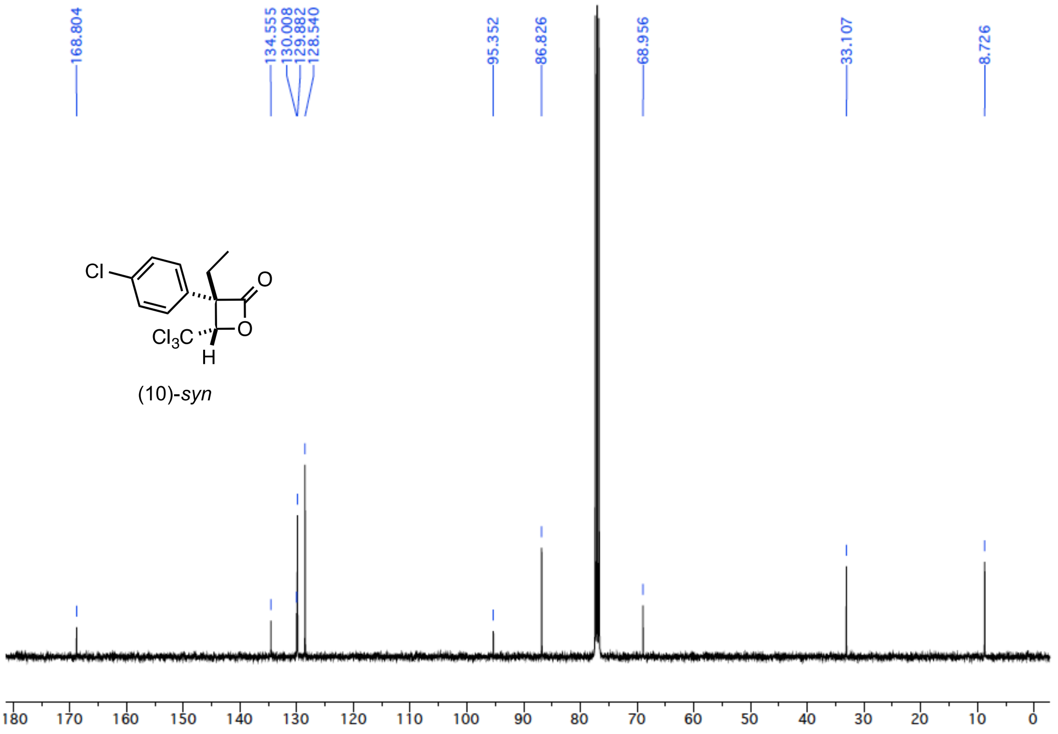

(11)-*Anti*-(3*S*,4*R*)-3-(4-bromophenyl)-3-ethyl-4-(trichloromethyl)oxetan-2-one

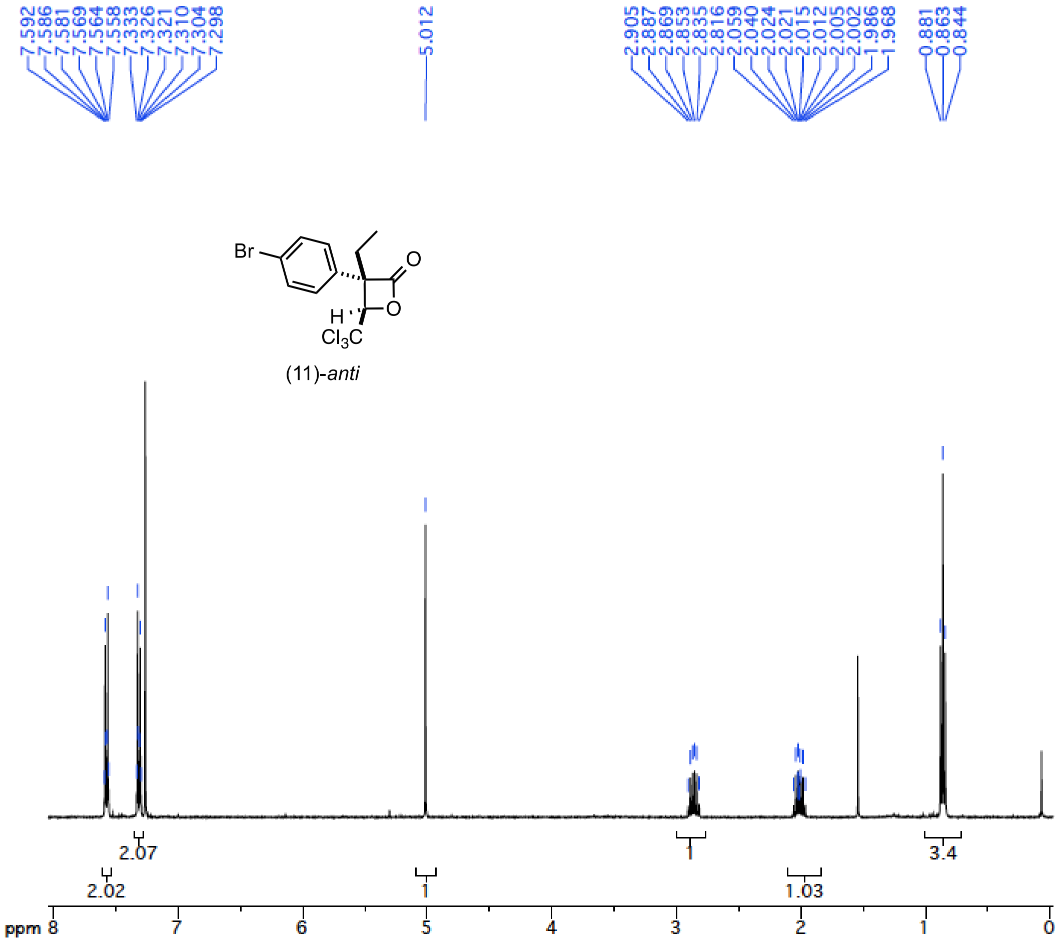

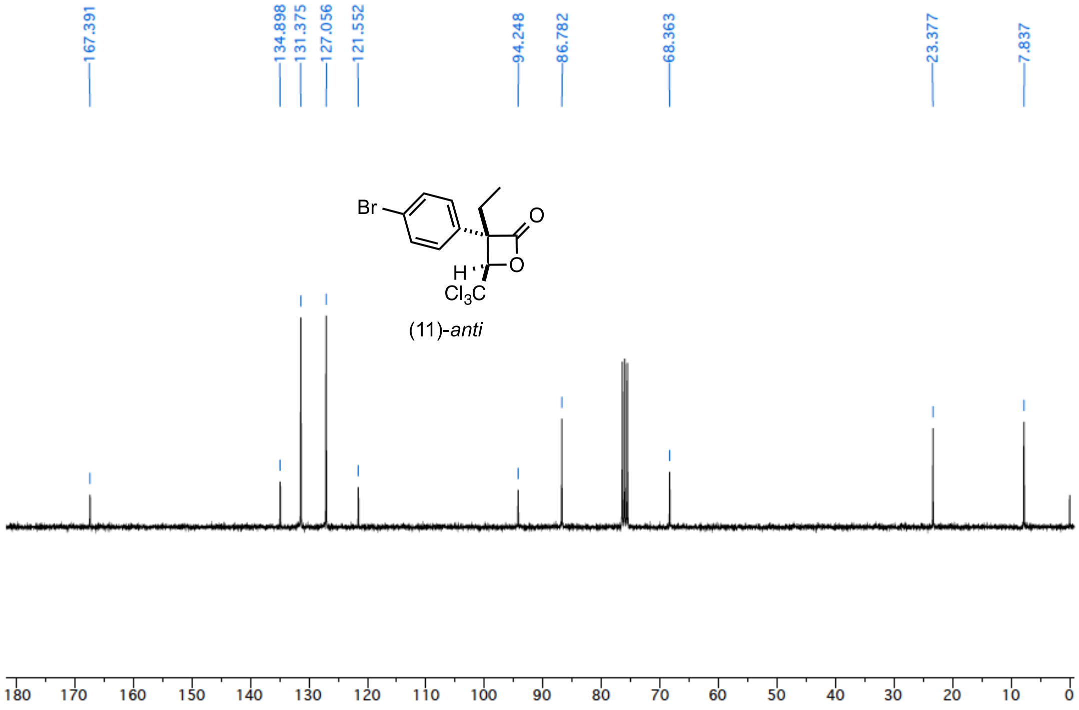

(11)-*Syn*-(3*S*,4*S*)-3-(4-bromophenyl)-3-ethyl-4-(trichloromethyl)oxetan-2-one

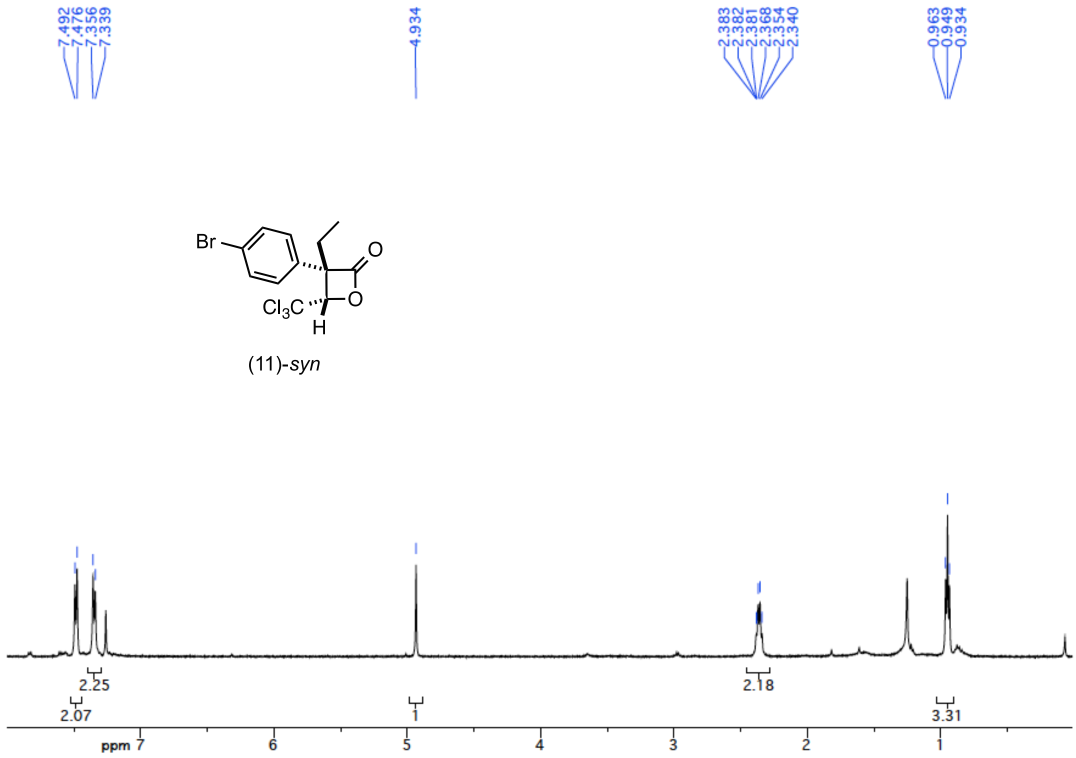

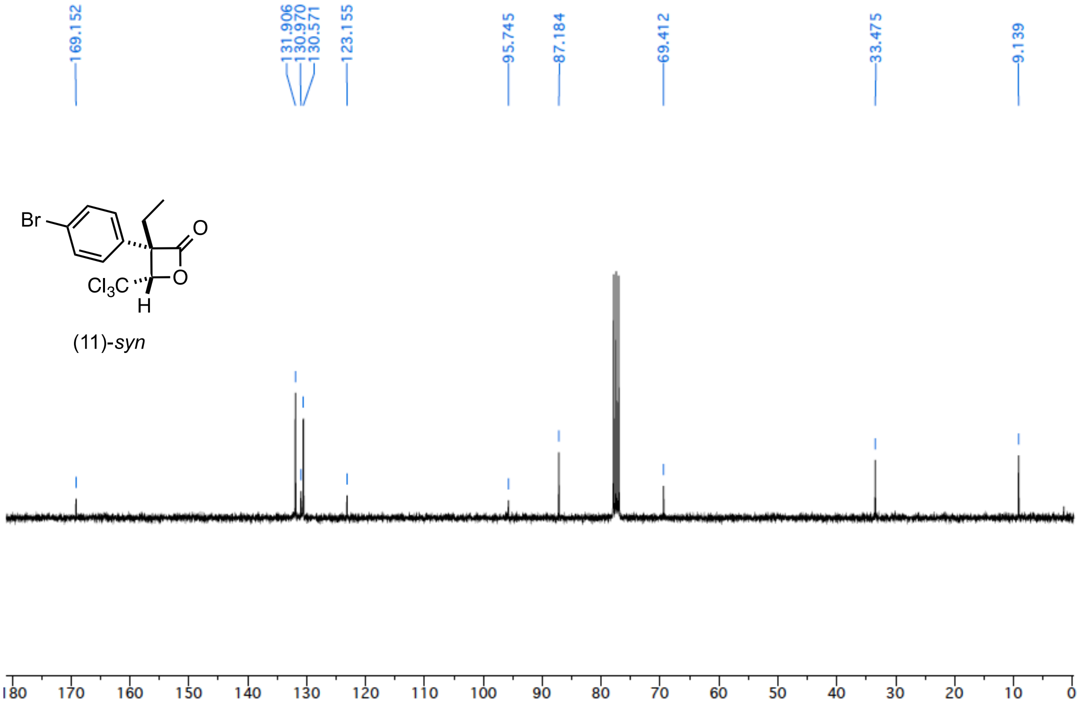

(12)-*Anti*-(3*S*,4*R*)-3-methyl-3-phenyl-4-(trichloromethyl)oxetan-2-one

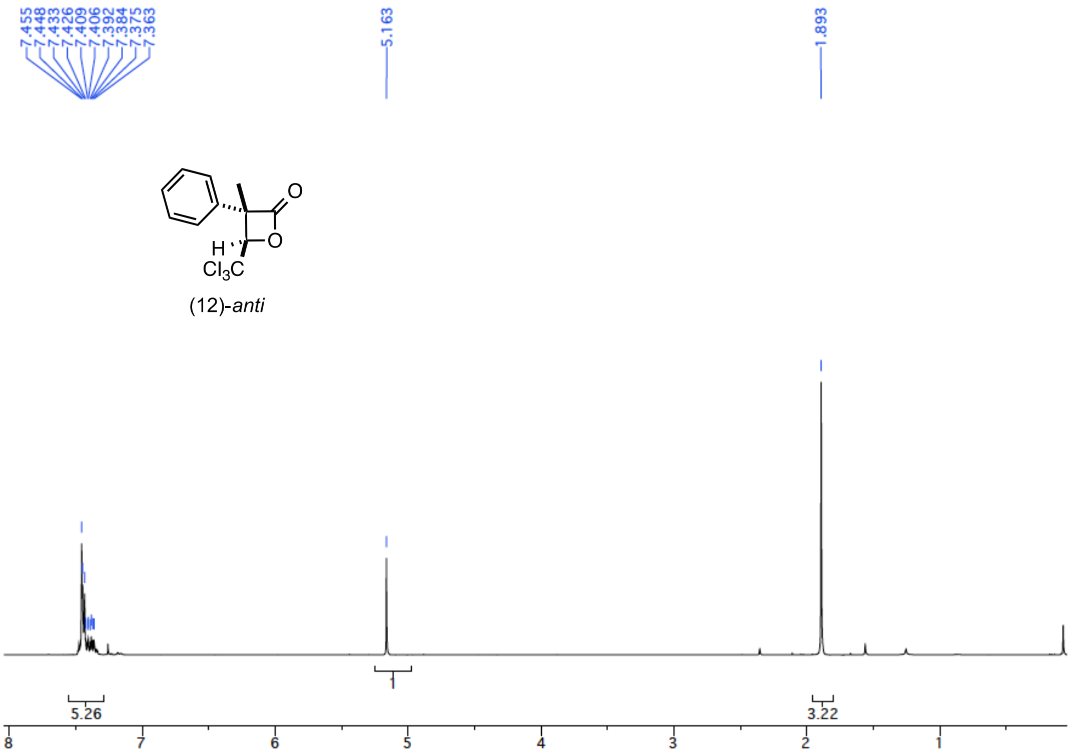

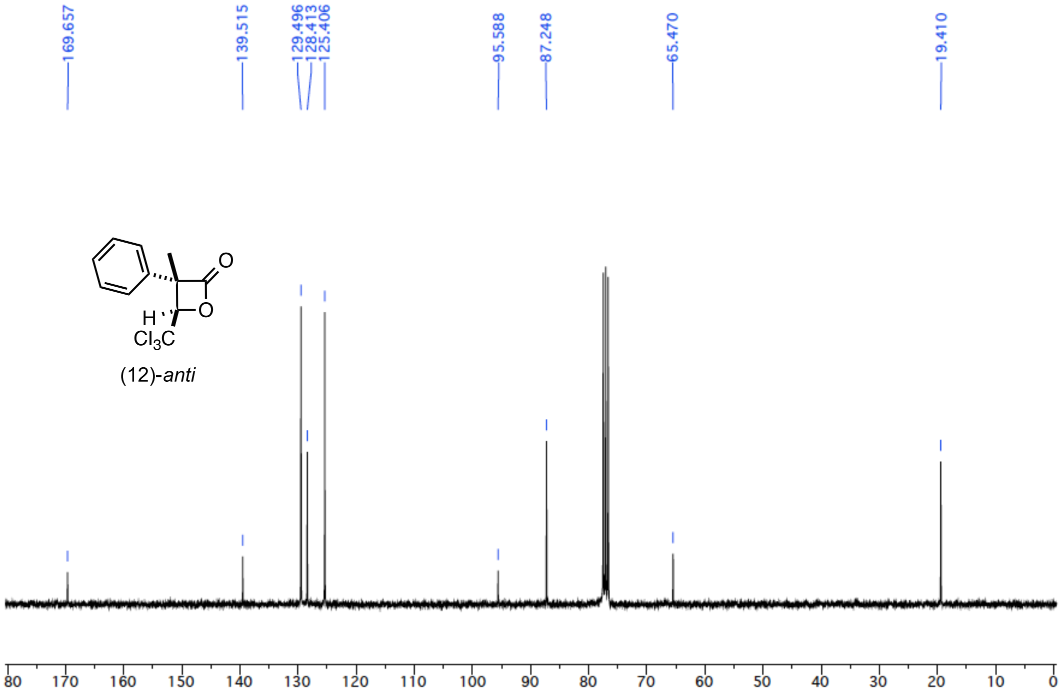

(13)-*Anti*-(3*S*,4*R*)-3-butyl-3-phenyl-4-(trichloromethyl)oxetan-2-one

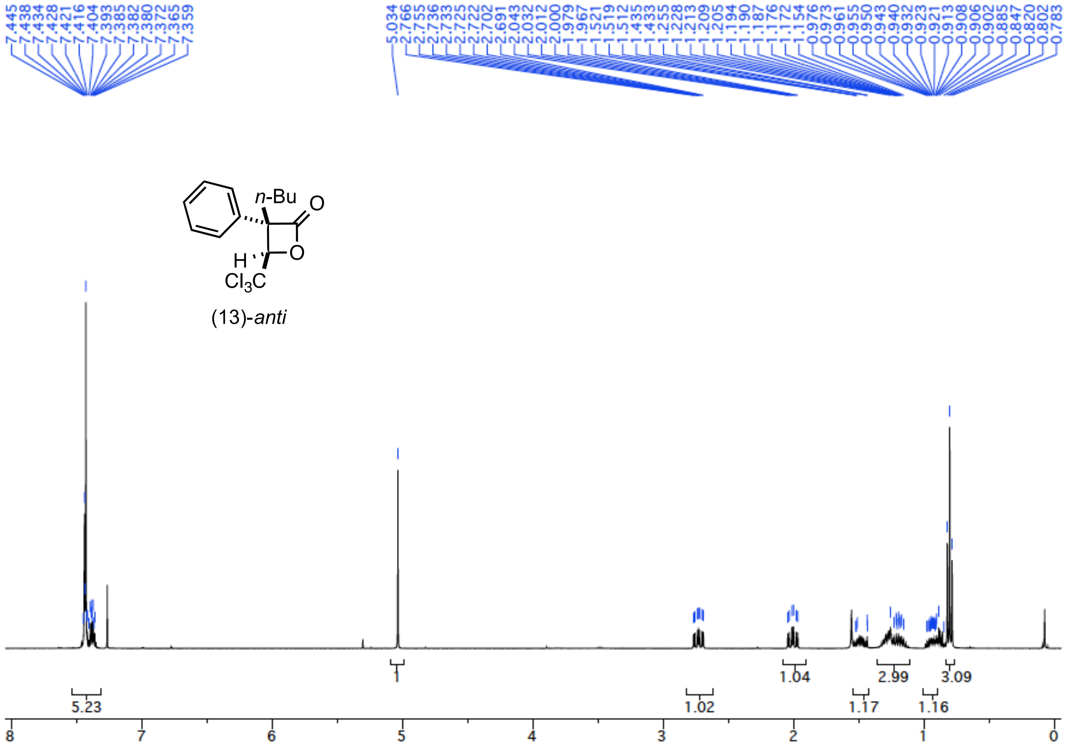

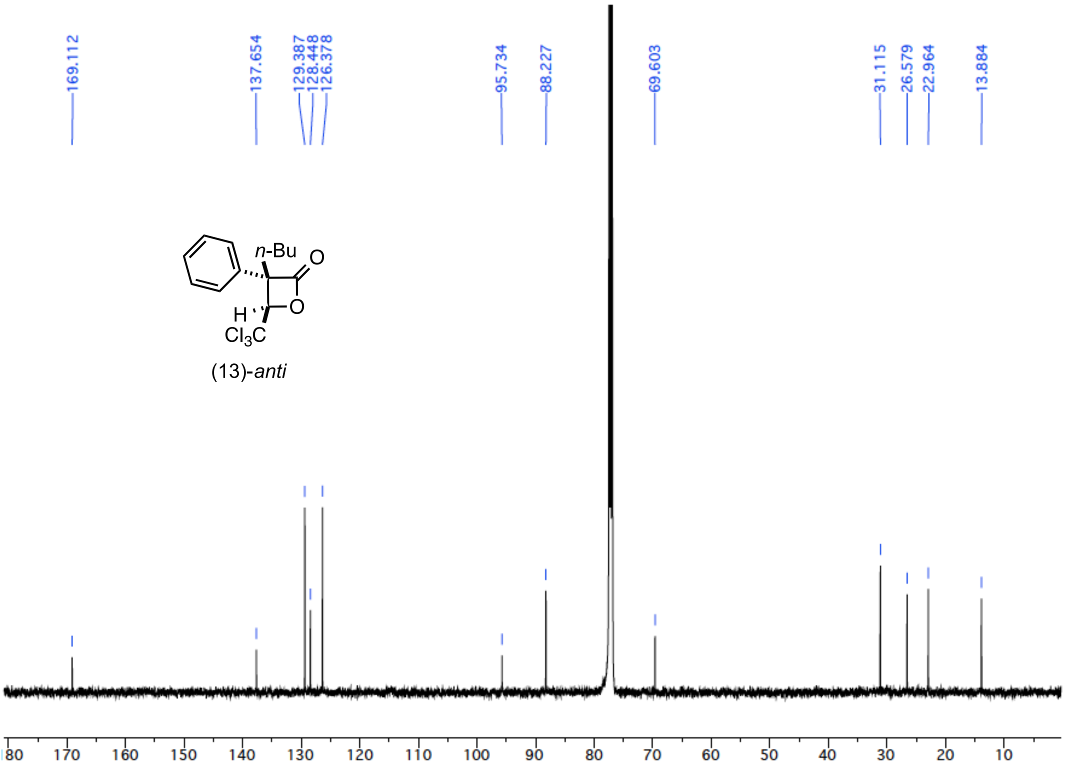

(14)-*Anti*-(3*S*,4*R*)-3-isobutyl-3-phenyl-4-(trichloromethyl)oxetan-2-one

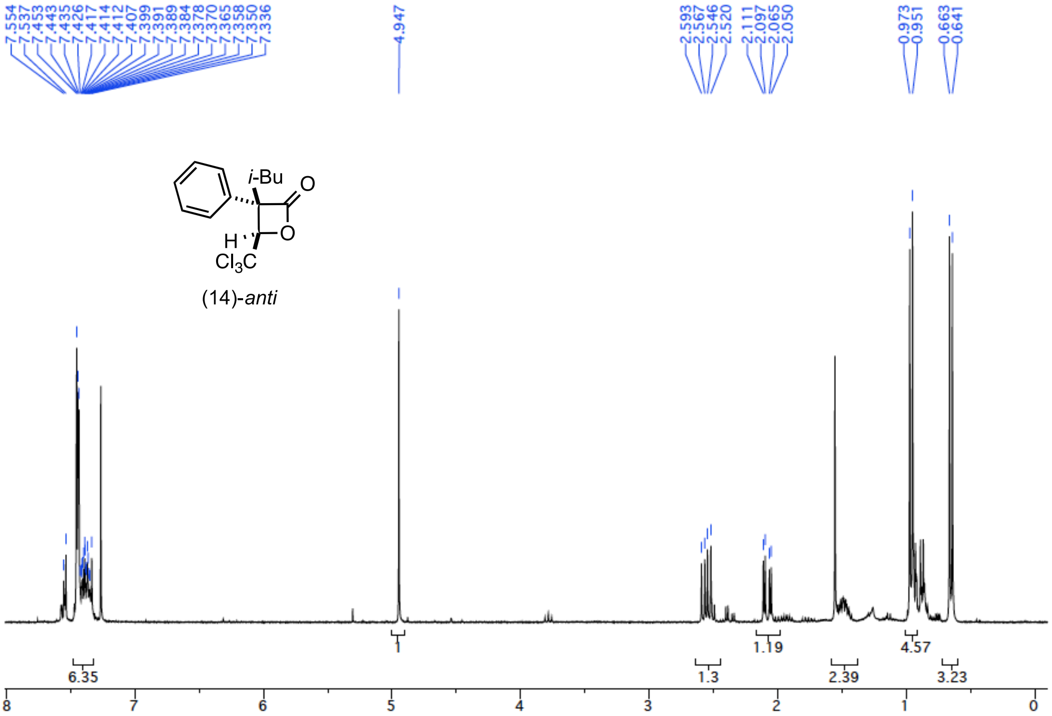

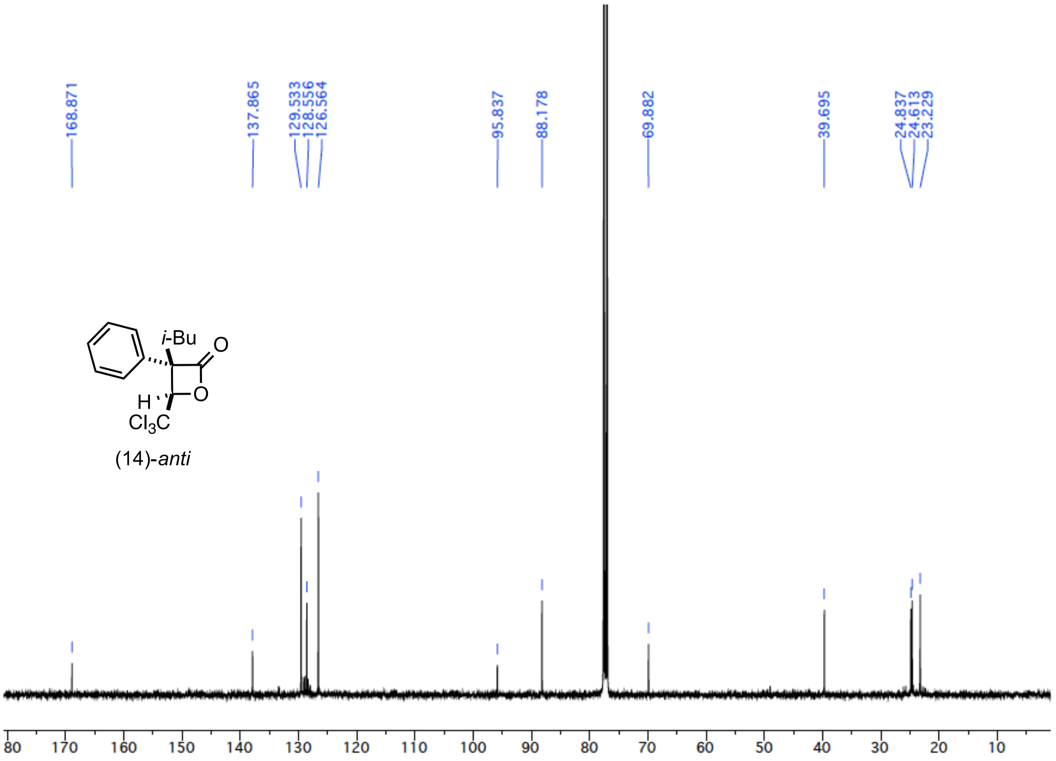

(14)-*Syn*-(3*S*,4*R*)-3-isobutyl-3-phenyl-4-(trichloromethyl)oxetan-2-one

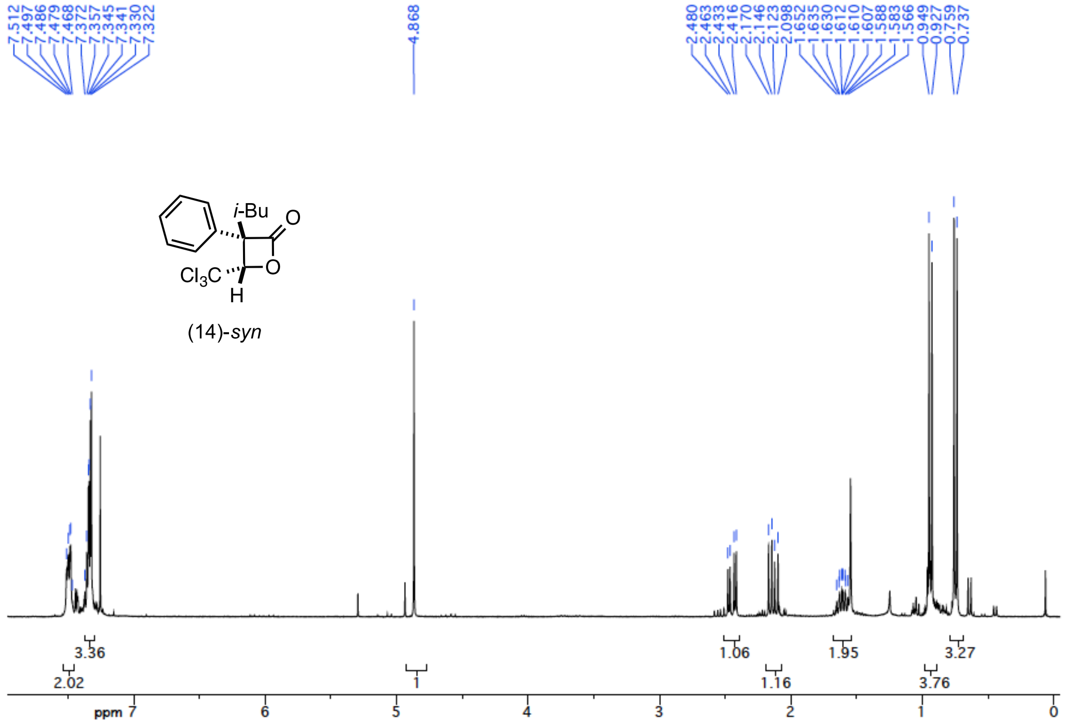

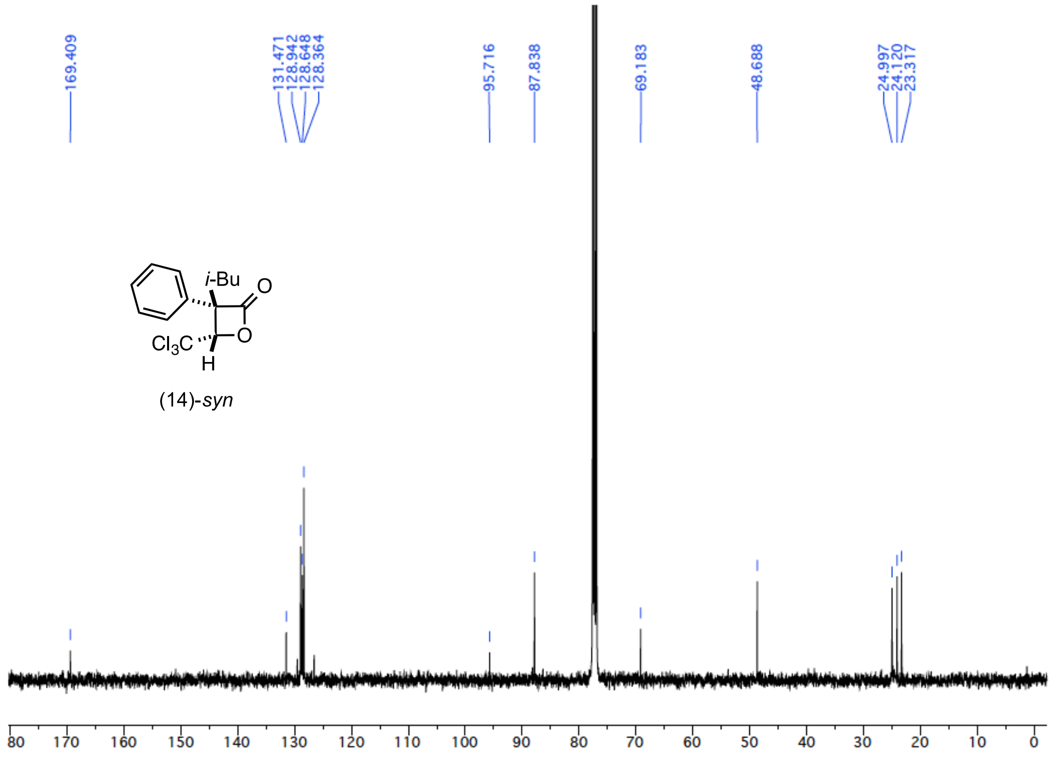

(**16**)-(*S*)-2,2-Dichlorovinyl 2-chloro-3-methyl-2-phenylbutanoate

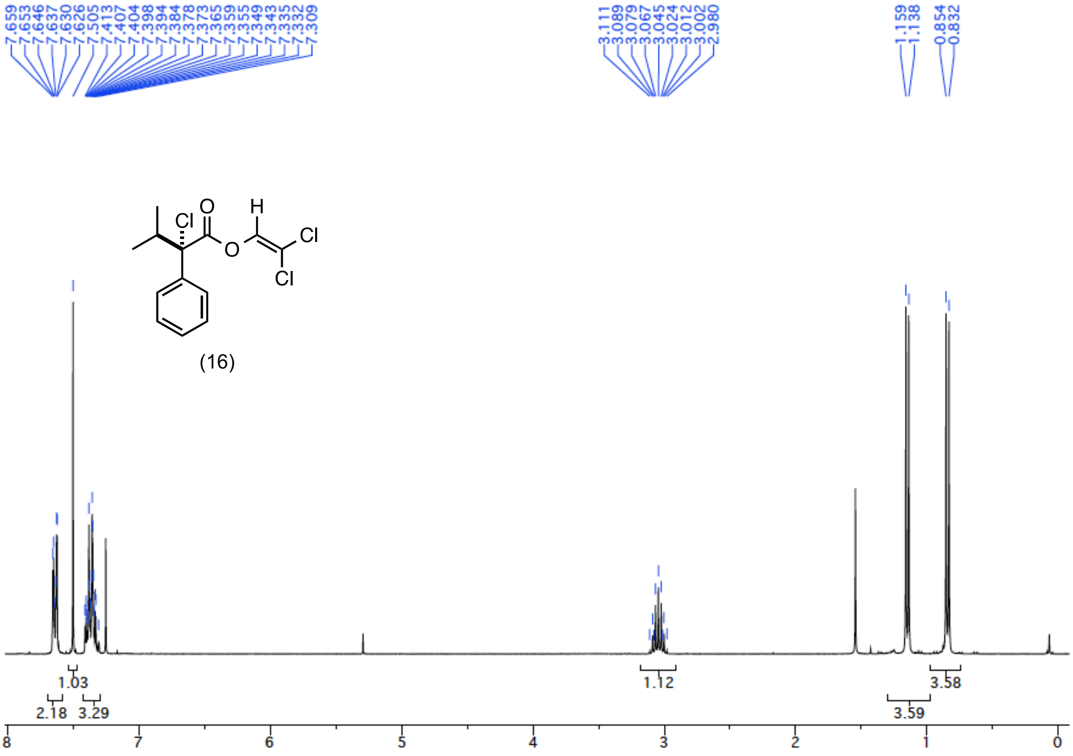

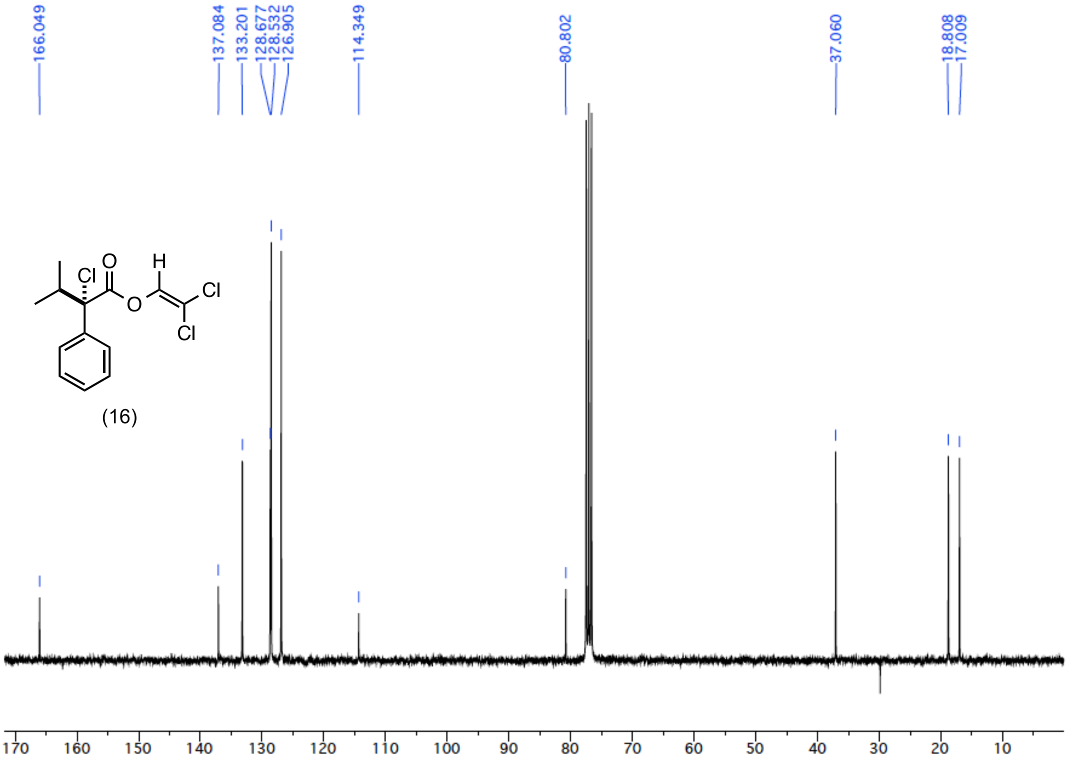

(17)-(*S*)-2,2-Dichlorovinyl 2-chloro-2-(2-tolyl)propanoate

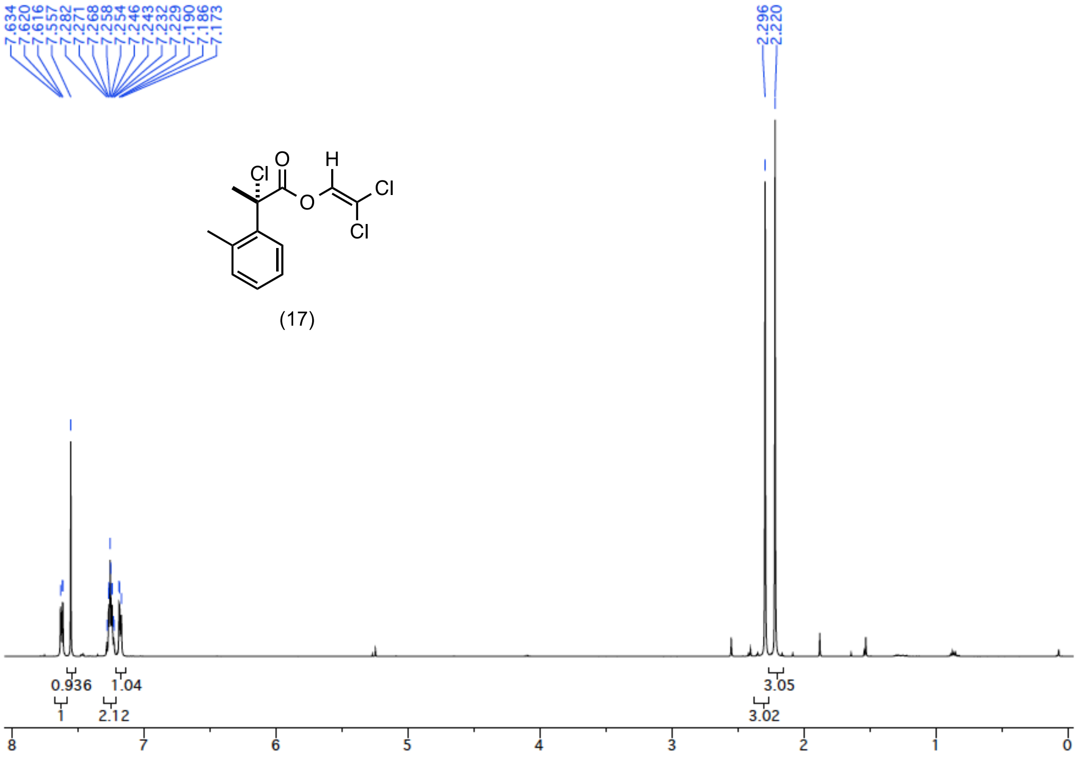

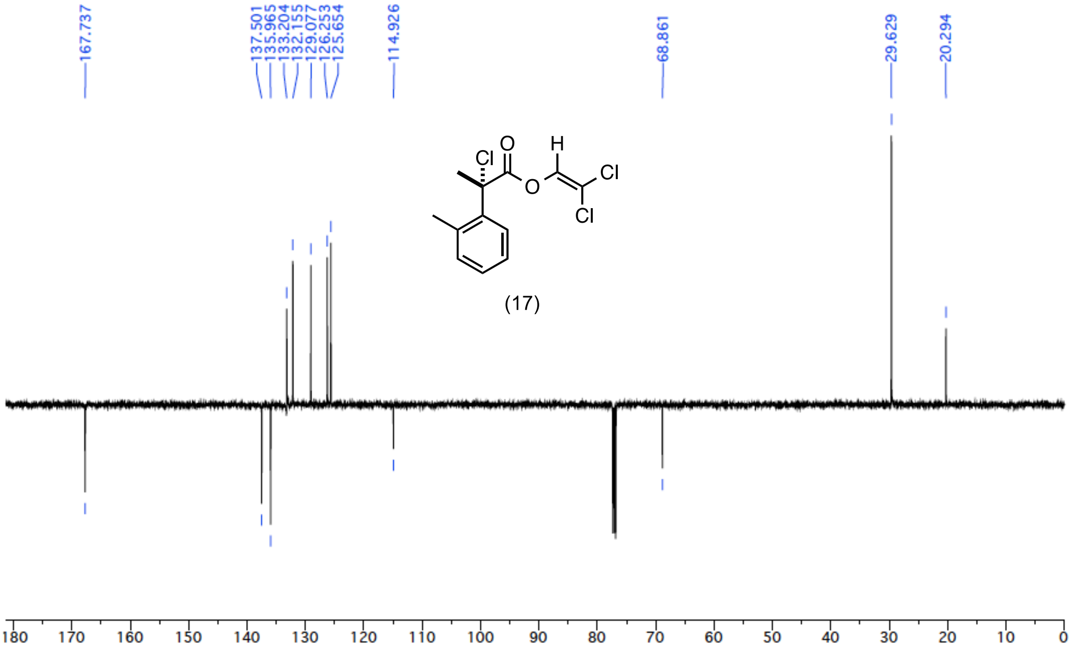

(18)-(*S*)-2,2-Dichlorovinyl 2-chloro-2-(2-tolyl)butanoate

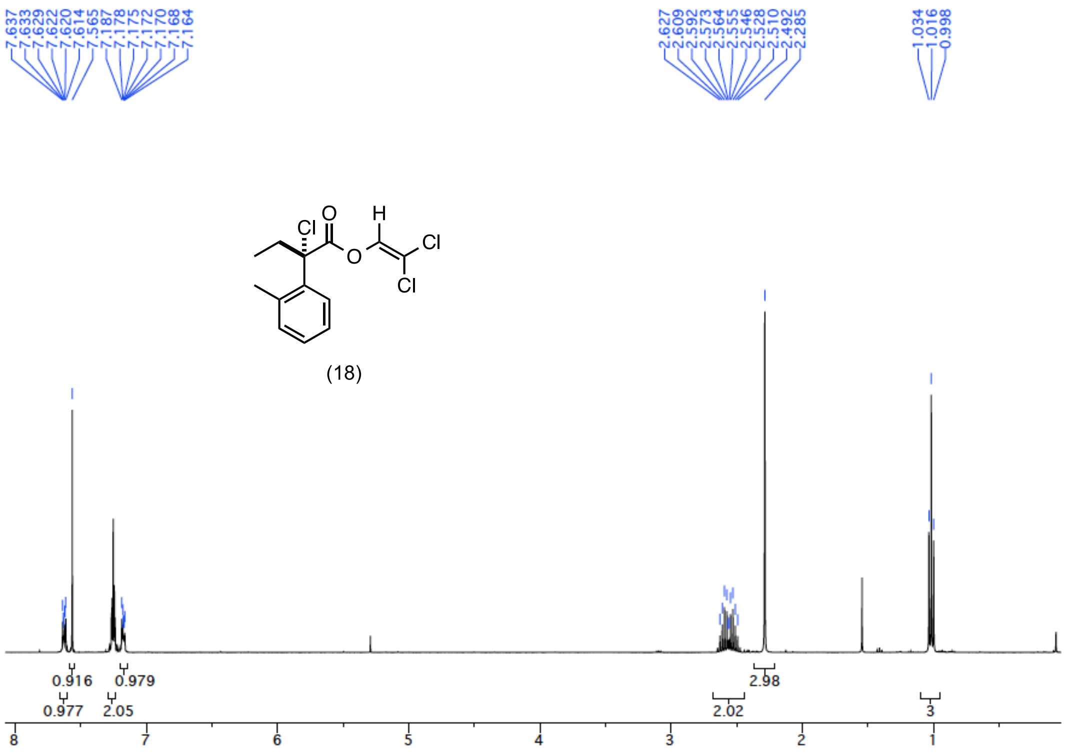

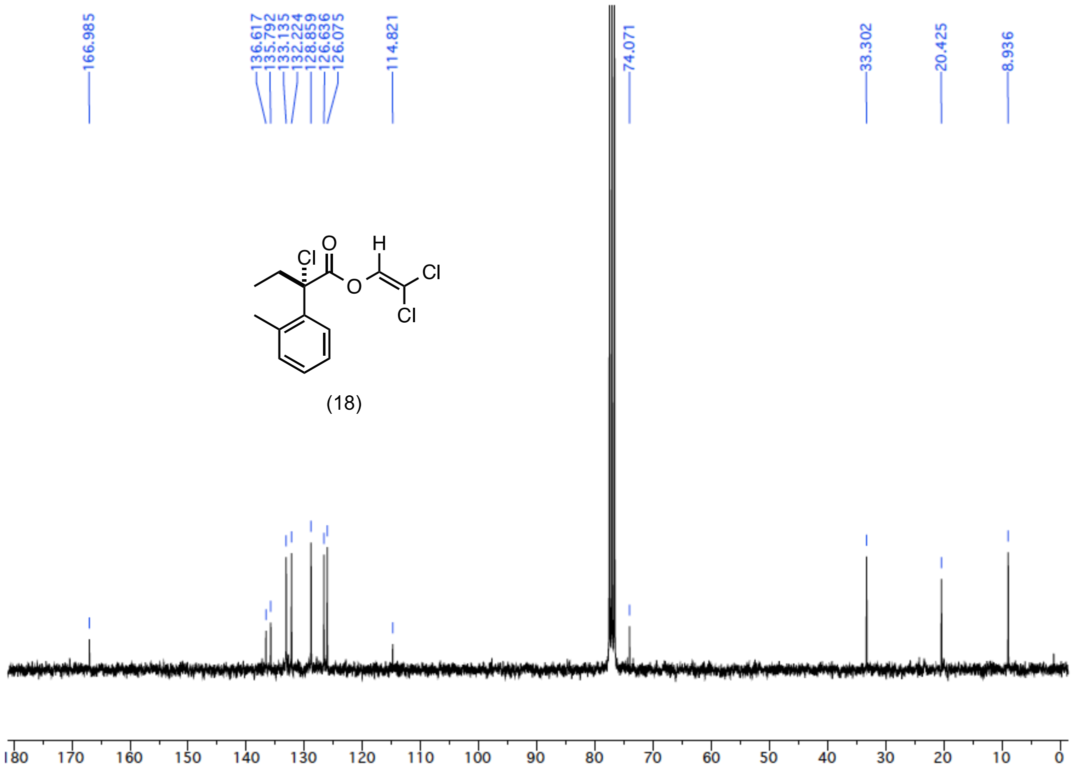

(19)-(*S*)-2,2-Dichlorovinyl 2-chloro-2-(naphthalene-1-yl)propanoate

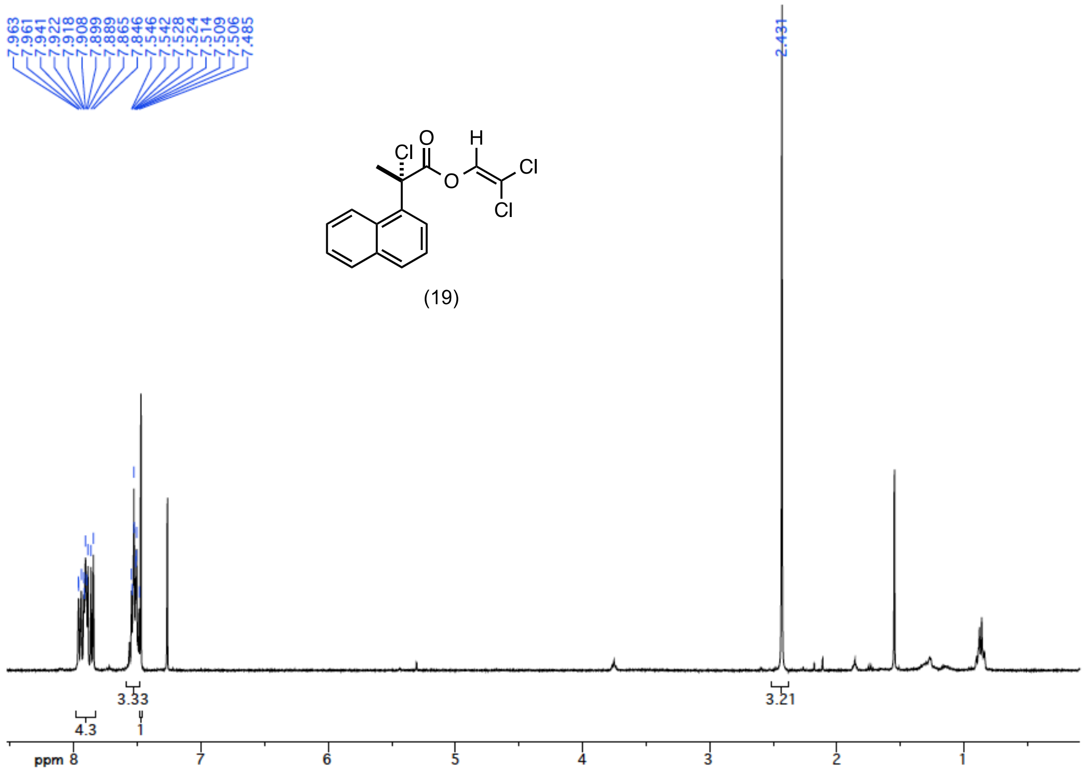

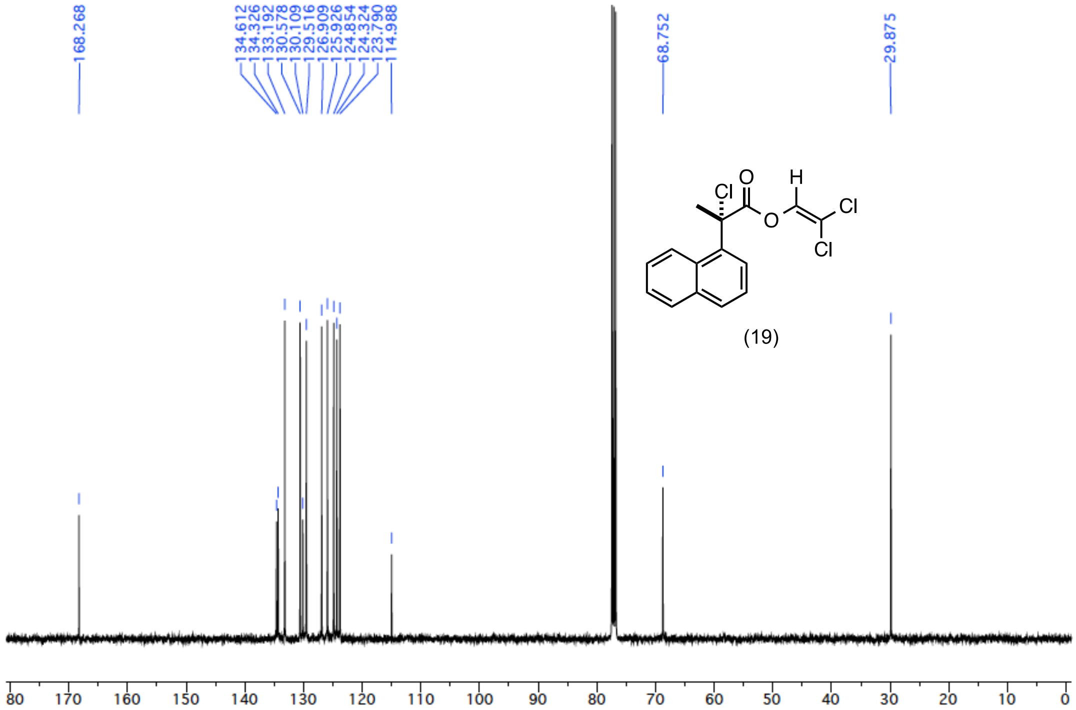

(20)-(*S*)-2,2-Dichlorovinyl 2-chloro-2-(2-chlorophenyl)butanoate

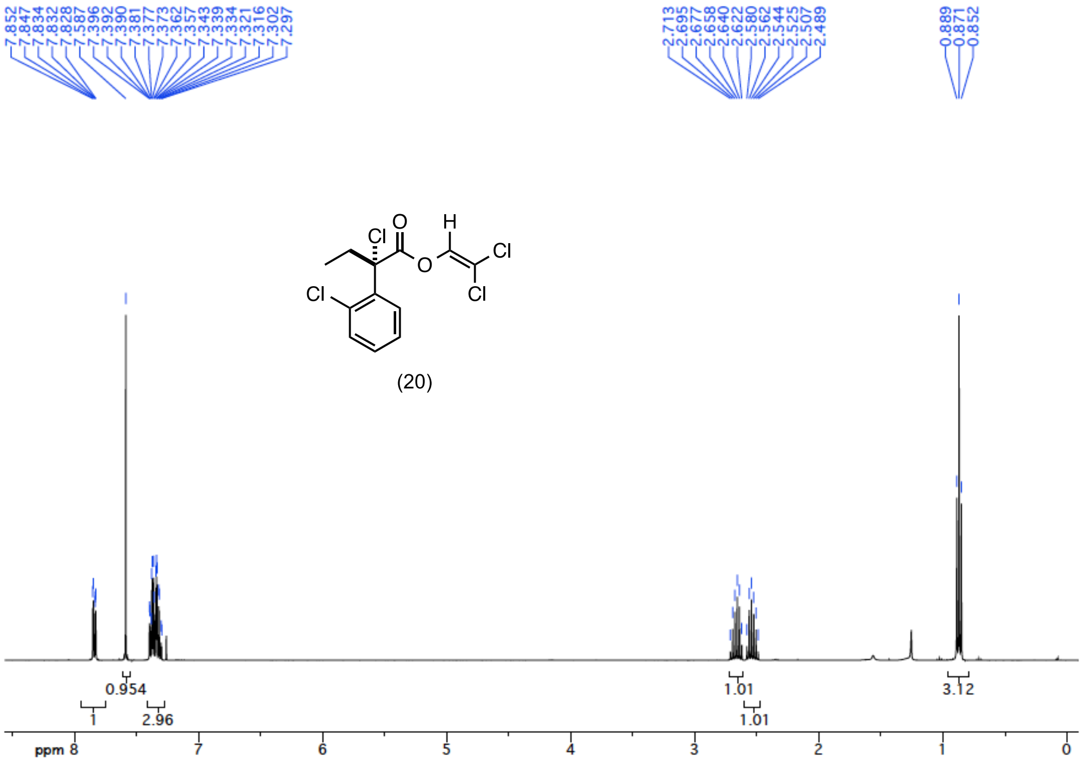

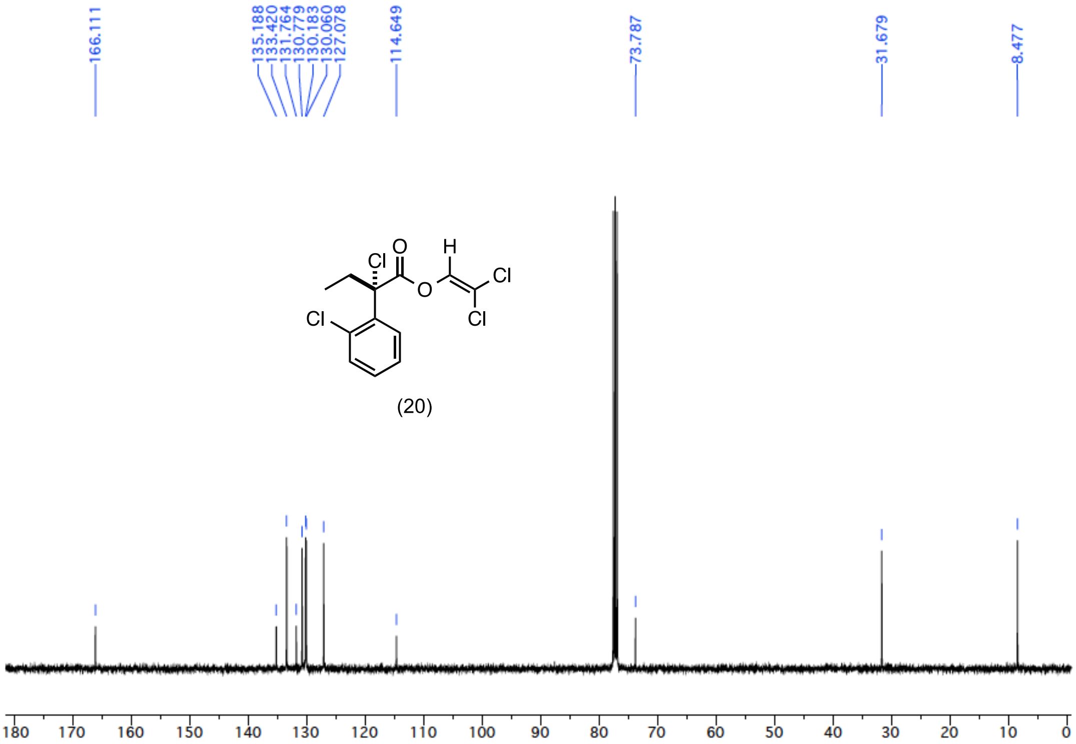

(21)-(*S*)-2,2-Dichlorovinyl 2-chloro-2-(naphthalene-1-yl)hexanoate

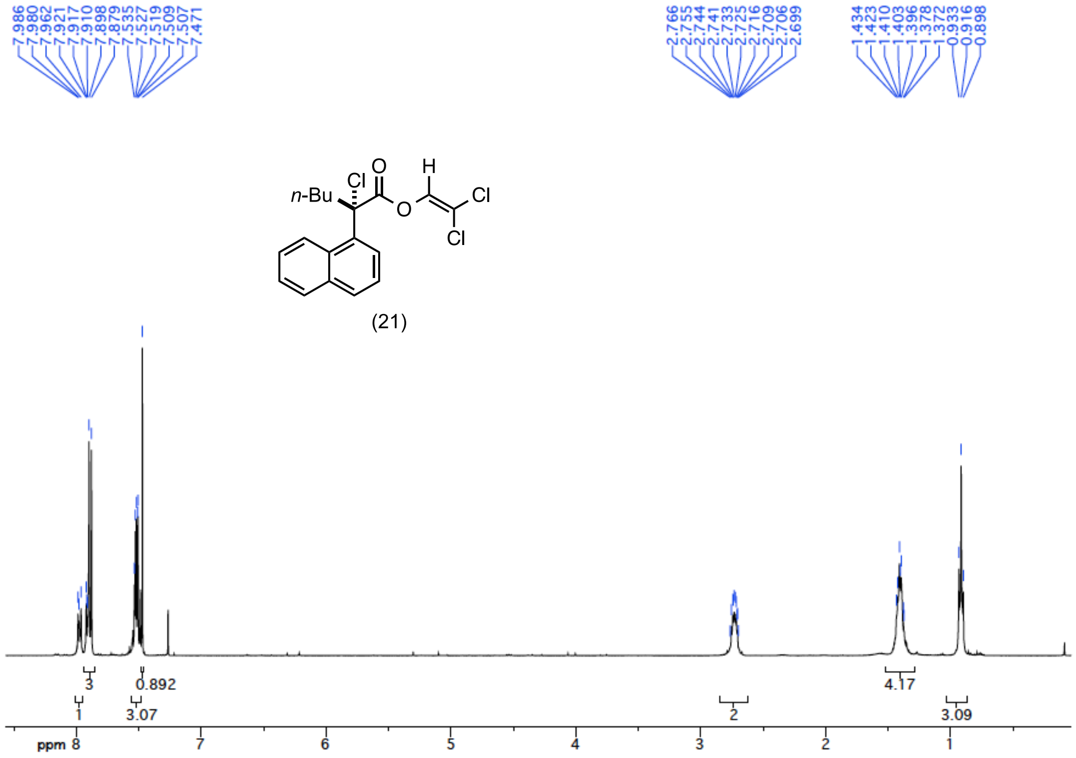

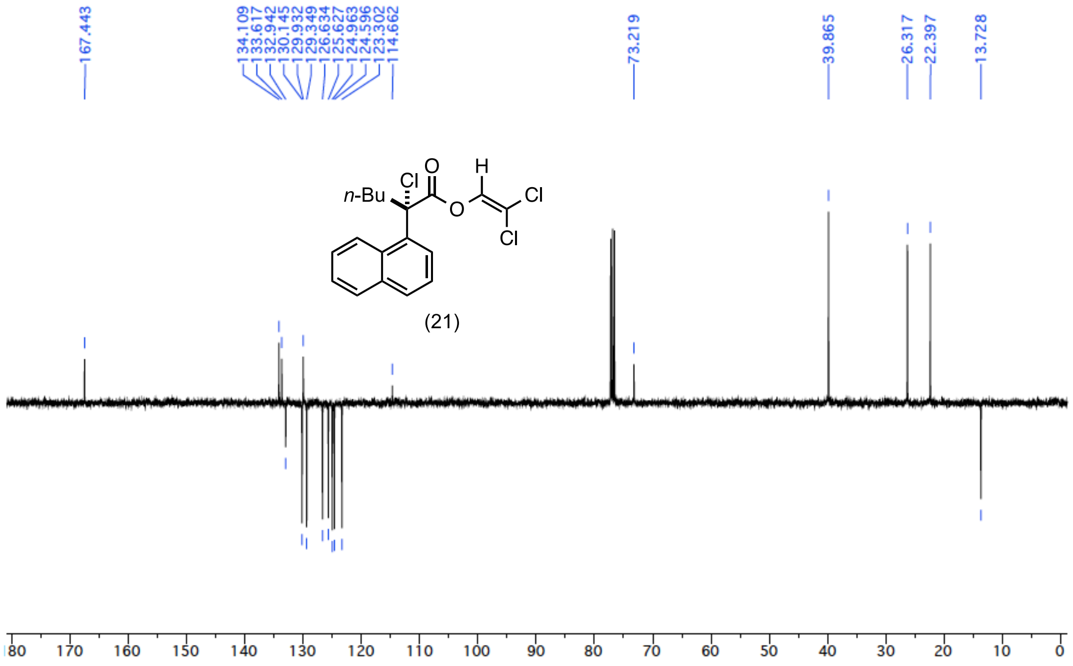

(**28**)-3-Ethyl-3-(2-methoxyphenyl)-4-(trichloromethyl)oxetan-2-one

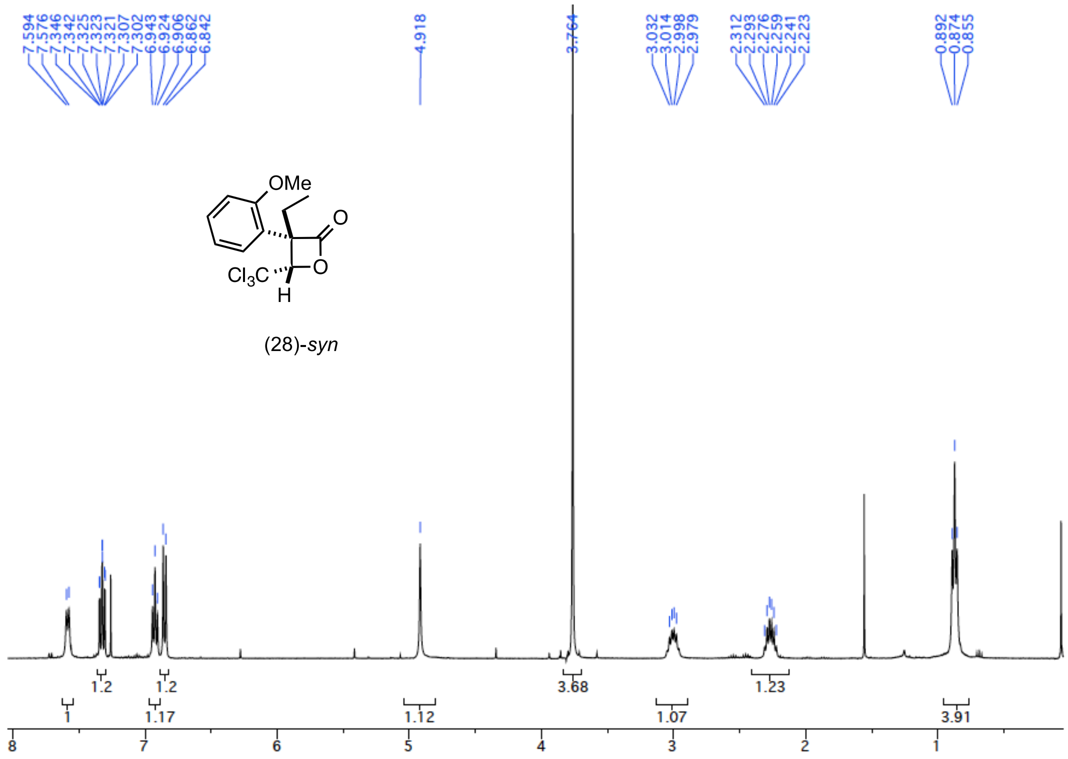

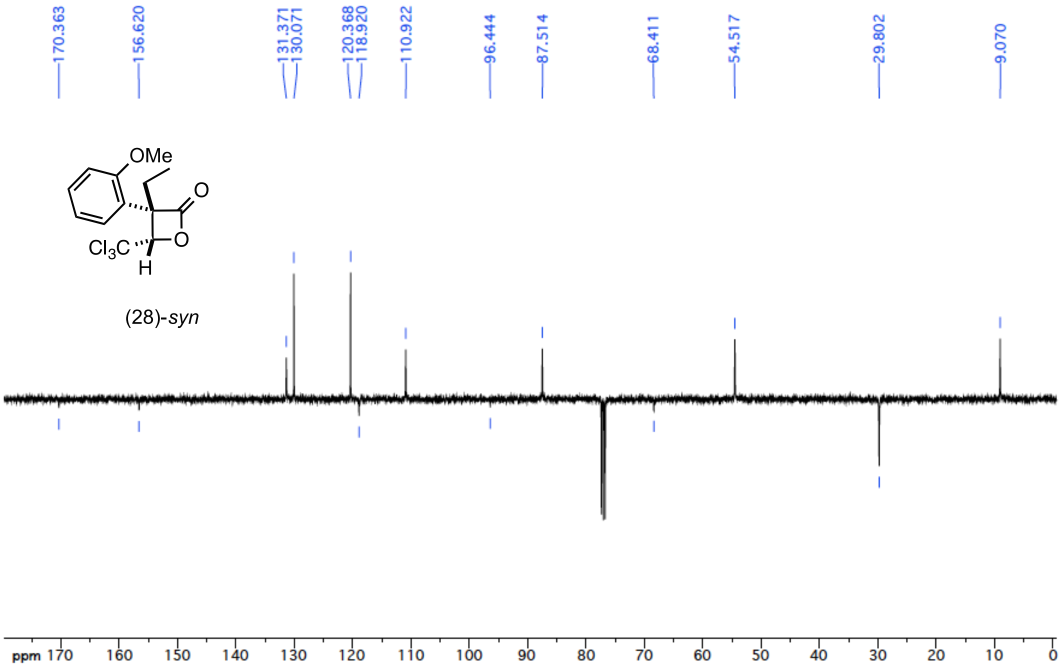

(**S9**)-2,2-Dichlorovinyl 2-chloro-2-(2-mhoxyphenyl)butanoate.

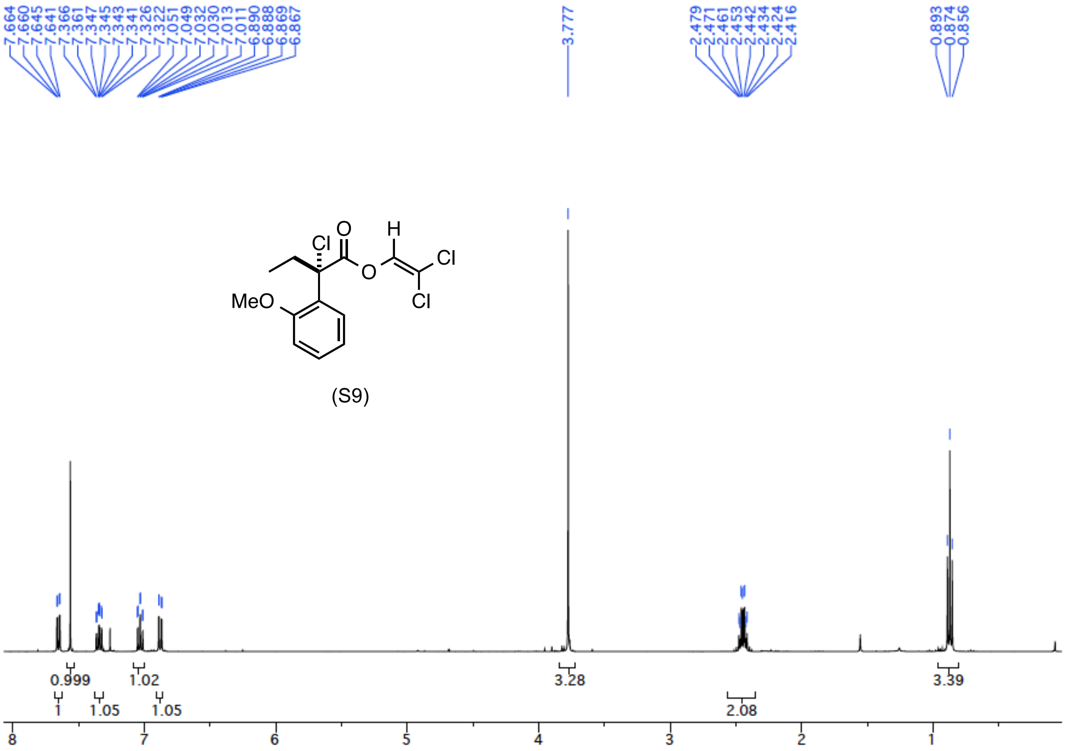

References

1. Concellón, C.; Duguet, N.; Smith, A. D., *Adv. Synth. Catal.* **2009**, *351*, 3001-3009.
2. Mermerian, A. H.; Fu, G. C., *Angew. Chem., Int. Ed.* **2005**, *44*, 949-952.
3. Baigrie, L. M.; Seiklay, H. R.; Tidwell, T. T., *J. Am. Chem. Soc.* **1985**, *107*, 5391-5396.
4. Frainnet, E.; Causse, J., *Bull. Soc. Chim. Fr.* **1968**, 3034.
5. Hodous, B. L.; Fu, G. C., *J. Am. Chem. Soc.* **2002**, *124*, 1578-1579.
6. Lv, H.; Zhang, Y.-R.; Huang, X.-L.; Ye, S., *Adv. Synth. Catal.* **2008**, *350*, 2715-2718.
7. Wiskur, S. L.; Fu, G. C., *J. Am. Chem. Soc.* **2005**, *127*, 6176-6177.
8. Dai, X.; Nakai, T.; Romero, J. A.; Fu, G. C., *Angew. Chem., Int. Ed* **2007**, *46*, 4367-4369.
9. Dochnahl, M.; Fu, G. C., *Angew. Chem., Int. Ed.* **2009**, *48*, 2391-2393.
10. Allen, A. D.; Baigrie, L. M.; Gong, L.; Tidwell, T. T., *Can. J. Chem.* **1991**, *69*, 138-145.
11. Bateman, L.; Breeden, S. W.; O'Leary, P., *Tetrahedron: Asymmetry* **2008**, *19*, 391-396.
12. Enders, D.; Han, J., *Tetrahedron: Asymmetry* **2008**, *19*, 1367-1371.
13. Zhang, Y.-R.; He, L.; Wu, X.; Shao, P.-L.; Ye, S., *Org. Lett.* **2007**, *10*, 277-280.
14. http://classic.chem.msu.su/gran/firefly/index.html
15. S. Grimme, S. Ehrlich, L. Goerigk, *J. Comp. Chem.* **2011**, 32, 1456-1465.
16. a) P.C. Hariharan and J.A. Pople, *Theoret. Chimica Acta*, **1973**, *28*, 213-222. b) M.M. Francl, W.J. Petro, W.J. Hehre, J.S. Binkley, M.S. Gordon, D.J. DeFrees and J.A. Pople, *J. Chem. Phys.*, **1982**, *77*, 3654.
17. F. Weigend, R. Ahlrichs, *Phys. Chem. Chem. Phys.* **2005**, 3297-3305
